# Genome-wide association study for yield-related traits in faba bean (*Vicia faba* L.)

**DOI:** 10.3389/fpls.2024.1328690

**Published:** 2024-03-13

**Authors:** Natalia Gutierrez, Marie Pégard, Ignacio Solis, Dejan Sokolovic, David Lloyd, Catherine Howarth, Ana M. Torres

**Affiliations:** ^1^ Área de Mejora Vegetal y Biotecnología, IFAPA Centro “Alameda del Obispo”, Córdoba, Spain; ^2^ INRA, Centre Nouvelle-Aquitaine-Poitiers, UR4 (URP3F), Lusignan, France; ^3^ Agrovegetal S.A., Sevilla, Spain; ^4^ Institute for Forage Crops, Kruševac, Serbia; ^5^ Institute of Biological, Environmental and Rural Sciences (IBERS), Aberystwyth University, Aberystwyth, United Kingdom

**Keywords:** yield, heritability, population structure, linkage disequilibrium, GWAS, MTA markers, candidate genes, faba bean

## Abstract

Yield is the most complex trait to improve crop production, and identifying the genetic determinants for high yield is a major issue in breeding new varieties. In faba bean (*Vicia faba* L.), quantitative trait loci (QTLs) have previously been detected in studies of biparental mapping populations, but the genes controlling the main trait components remain largely unknown. In this study, we investigated for the first time the genetic control of six faba bean yield-related traits: shattering (SH), pods per plant (PP), seeds per pod (SP), seeds per plant (SPL), 100-seed weight (HSW), and plot yield (PY), using a genome-wide association study (GWAS) on a worldwide collection of 352 homozygous faba bean accessions with the aim of identifying markers associated with them. Phenotyping was carried out in field trials at three locations (Spain, United Kingdom, and Serbia) over 2 years. The faba bean panel was genotyped with the Affymetrix faba bean SNP-chip yielding 22,867 SNP markers. The GWAS analysis identified 112 marker–trait associations (MTAs) in 97 candidate genes, distributed over the six faba bean chromosomes. Eight MTAs were detected in at least two environments, and five were associated with multiple traits. The next step will be to validate these candidates in different genetic backgrounds to provide resources for marker-assisted breeding of faba bean yield.

## Introduction

Faba bean (*Vicia faba* L.) has been cultivated since the beginning of agriculture (Cubero, 1973) and, at present, is the fourth most widely grown cool season legume after chickpea, pea, and lentil (FAOSTAT, 2020). Its nutrient-rich seeds are used as a protein source for both human consumption and as a feed grain. It is well adapted to a wide range of climatic areas and has one of the highest yield potentials (Cernay et al., 2016) and protein content (average ~29%) of the grain legumes (Warsame et al., 2018).

Legumes play a crucial role in ensuring worldwide food security, promoting agroecosystem resilience, and facilitating sustainable agriculture. The key benefits of legumes include the biological fixation of atmospheric nitrogen as a result of a symbiotic relationship with soil bacteria known as rhizobia. Among grain legumes, faba bean is the most efficient nitrogen fixer with values ranging between 50 and 200 kg N ha^−1^ (Schwenke et al., 1998; Carranca et al., 1999; Lopez-Bellido et al., 2006). This unique ability reduces the dependence of farmers on extensive use of fertilizers and protects soil and groundwater quality. Furthermore, implemented in crop rotations, faba bean functions as a break crop, decreasing the occurrence of weeds, pests and diseases and enhancing soil physical conditions. This leads to a higher yield of subsequent crop(s) while reducing the amount of fertilizers and biocides required (Köpke and Nemecek, 2010; Cernay et al., 2016).

Despite these nutritional and multiple environmental services, faba bean represents a minor part of European agricultural systems that have developed a high dependence on imports of grain legumes. This is partly due to a lack of economically competitive grain legume crops that can match cereals in terms of farmer profits. For the faba bean to become an economically more attractive crop and thus increase the area under cultivation, major advances in both yield and yield stability need to be achieved. Currently, faba bean yield is considered to be highly unreliable due to a significant level of genotype × environment interaction (Cernay et al., 2016). A greater understanding of the main biological and environmental factors affecting plant growth and the identification of the main yield components are critical to improve adaptation and yield in this crop. While biotic and abiotic stresses have received considerable attention in faba bean (Adhikari et al., 2021; Khazaei et al., 2021), plant architecture and yield-related traits are still poorly understood in this crop. Faba bean has lagged behind cereals in the genetic improvement of yield, due to a combination of less investment and limited genomic resources available (Adhikari et al., 2021). Recent advancements in genomic tools, such as a reference genome sequence (Jayakodi et al., 2023), enable a genomics-based breeding platform for assisting conventional breeding and accelerate the release of high-yielding and stable faba bean cultivars. Quantitative trait loci (QTLs) associated with yield-related traits have been identified previously, but the results were limited due to the use of anonymous markers and low-density maps (Ramsay et al., 1995; Avila et al., 2005). Stable QTLs for three reproductive traits were identified on chromosomes (Chr.) II and V (pod length) and VI (number of ovules per pod and number of seeds per pod), by evaluating the recombinant inbred line (RIL) population Vf6 × Vf27 (Cruz-Izquierdo et al., 2012) during two seasons. More recently, 11 plant architecture and yield-related traits were recorded in the same population across four seasons (Ávila et al., 2017), confirming the QTLs for pod length, number of ovules per pod, and number of seeds per pod and further identifying stable QTLs for hundred-seed weight on Chr. V and VI. Despite these valuable advances, the genetic architecture and determinants of faba bean yield remain uncertain.

The resolution and accuracy with which QTL mapping can identify the direct causal gene(s) controlling a trait are limited by the large confidence intervals and the relatively low number of recombination events existing in biparental mapping populations (Beji et al., 2020). With the recent development of powerful faba bean SNP array platforms (Khazaei et al., 2021), genome-wide association studies (GWAS) are now an additional tool to dissect polygenic traits by utilizing the genetic diversity and historical recombination events present in wide germplasm collections. GWAS aims at identifying markers strongly associated with quantitative traits by using the linkage disequilibrium (LD) between candidate genes and markers. Compared with family-based QTL mapping, GWAS significantly increases mapping resolution and enables minor effect genes to be detected (Abdurakhmonov et al., 2008). In recent years, GWAS studies have been reported in a range of legume crops such as soybean (Hwang et al., 2014), pigeon pea (Varshney et al., 2017), common bean (Raggi et al., 2019), chickpea (Varshney et al., 2019), red clover (Zanotto et al., 2023), alfalfa (Pégard et al., 2023), and the model legume *Medicago truncatula* (Bonhomme et al., 2014). In faba bean, only a few GWAS studies have been reported so far which have identified candidate genes associated with frost tolerance (Sallam et al., 2016); resistance to *Ascochyta fabae* (Faridi et al., 2021); tolerance to herbicides (Abou-Khater et al., 2022); drought, heat, and freezing tolerance (Ali et al., 2016; Maalouf et al., 2022; Gutiérrez et al., 2023); agronomic traits (Skovbjerg et al., 2023); and seed size (Jayakodi et al., 2023).

The aim of this work was to identify for the first time genomic regions controlling yield-related traits using a panel of 400 faba bean lines grown in a range of environments and using the recently available 60K Axiom Vfaba_v2 SNP array (O’Sullivan et al., 2019; Khazaei et al., 2021). The traits analyzed were pod shattering (SH), seeds per pod (SP), pods per plant (PP), seeds per plant (SPL), 100 seed weight (HSW), and plot yield (PY). The results obtained contribute to the discovery of new genomic regions associated with grain yield characters and the identification of candidate genes associated with the SNPs to accelerate future molecular marker-assisted breeding in this crop.

## Materials and methods

### Plant material

The faba bean EUCLEG collection consists of 400 accessions from Africa (25 accessions); North, Central, and South America (10); Asia (59); and Europe (185). Moreover, it also consists of 121 accessions with unknown origin. Europe, with 22 countries, is the most represented continent in the panel, followed by Asia, America, and Africa (13, 4, and 5 countries, respectively). Spain accounts for the highest number of accessions (68). The panel includes 81 breeding and advanced materials, 47 varieties, 7 parental inbred lines from different mapping populations, and 265 accessions from different germplasm banks (**Supplementary Table S1**). The selection was made in collaboration with public institutes including ICARDA, IFAPA, IFVCNS, INIA, and INRA; the universities of Ghent and Göttingen; and the following gene banks: ESP004, ESP046, FRA043, SWE054, SYR002, and NordGen. Private sector contributions consisted of lines/varieties from the companies Agrovegetal, NPZ, Batlle, and Fitó. Since the faba bean is a partially allogamous species, prior to genotyping, all the lines from Spain had been selfed at IFAPA, Córdoba, in the field for at least four generations using insect-proof cages with the remaining accessions selfed for two generations.

### Phenotypic data analysis

#### Phenotypic traits and experimental design

The faba bean collection was phenotyped in three geographic locations: 1) Escacena del Campo, Huelva (Spain; 2019 and 2020) by the company Agrovegetal; 2) Aberystwyth (United Kingdom (UK); 2019) by IBERS; and 3) Krusevac (Serbia; 2020) by IKBKS. The growing season in southern Spain was from November to June, while in the rest of the countries, it was from April to September. The location descriptors are as follows: Escacena del Campo (37°30′N, 6°22′W), Aberystwyth (52°41′N, 4°06′W), and Krusevac (43°58′N, 21°20′E).

In each location and year, the accessions were arranged in the field following an augmented design MAD type 2 (Lin and Poushinsky, 1983). The experimental trials included 440 plots (3 m² each), distributed in 22 rows and 20 columns. Plots consisted of 4 rows of 2 m length with 0.5 m row spacing and seeding distance of 10 cm (80 seeds/plot). Twenty accessions were chosen as checks and distributed in four incomplete blocks with different numbers of repetitions (5 checks repeated six times and 15 twice), and the remaining 380 accessions were unreplicated and randomly distributed within and between blocks.

The plants were measured and scored for six agronomic traits affecting the final yield response. Pods per plant (PP), seeds per pod (SP), and seeds per plant (SPL) were recorded as the mean value from 10 plants in the central rows. Plot yield (PY) was determined as the weight of the seeds from a whole plot in kilograms (kg). The seeds were used to determine 100 seed weight (HSW) in grams (g). Shattering (SH) was determined just before harvesting only at Escacena del Campo, Spain, in 2019 using a 0 to 3 scale as follows: indehiscent (0), fissured with valves slightly open along the ventral suture (1), dehiscent (DH) with non-twisting valves (2), and dehiscent with twisting valves (3). PP and SPL were not scored in the UK and the rest of the traits were recorded in all locations.

### Genomic data analysis

#### SNP genotyping and quality control

For DNA extraction, young leaf tissue was collected from a single plant per accession. Leaf samples were frozen and stored at −80°C until used. Genomic DNA was extracted using a DNeasy Plant Mini Kit (Qiagen Ltd., UK), and DNA quality was assessed as described in Gutiérrez et al. (2023). Pure and good-quality DNA samples with an average concentration of 40 ng/µl were used for genotyping using the Vfaba_v2 Axiom SNP array with 60K SNP (O’Sullivan et al., 2019; Khazaei et al., 2021) from Affymetrix (Thermo Fisher Scientific), University of Reading, UK. After quality control, 26 accessions with poor DNA quality and 22 revealing a low number of SNPs were excluded from the analysis. SNP calling was performed on the remaining 352 samples, and SNP markers with a call rate below 97% were discarded from the final genotyping database. The SNPs were filtered to remove those with a minor allele frequency (MAF)<0.05 and >15% of heterozygotes. The missing values were imputed with the minor allele frequency (Badke et al., 2014), and it represented less than 1% of all genomic data. The allelic genotyping matrix was transformed into a numeric format (0, for the reference allele; 1, for the heterozygous allele; and 2, for the alternative allele).

#### Genomic relationship matrix

The genomic relationship matrix (*G*) was constructed based on VanRaden (VanRaden, 2008) (Equation 1):


(1) $$G=\frac{ZZ'}{2\;\sum^{}p_{i}\left(1-p_{i\;}\right)}\;$$

where the matrix **Z** was calculated as (**M** − **P**). **M** is a matrix of minor allele counts (0, 1, and 2 for the reference, heterozygote, and alternative, respectively) with *m* columns (one for each marker) and *n* rows (one for each accession). **P** is a matrix that contains the minor allele frequency, expressed as a difference from 0.5 and multiplied by 2, such that column *i* of P is 2(p*_i_* − 0.5).

#### Analysis of phenotypic data

For phenotypic analysis, environments were designed as follows: *Spain.2019* (Spain, season 2018/2019), *Spain.2020* (Spain, season 2019/2020), *Spain.Global* (combined data in Spain for two seasons), *UK.2019* (United Kingdom, year 2019), *Serbia.2020* (Serbia, year 2020), and *Global* (all environments combined). The check accessions were used to capture the spatial heterogeneity at the plot level, and all traits were independently adjusted for field microenvironmental heterogeneity using a mixed linear model (MLM) by the function restricted maximum likelihood (REML) with the “breedR” package (Muñoz and Sanchez, 2020). A random effect was fitted using the tensor product of two B-spline bases with a covariance structure for the random knot effects (RKE) to account for spatial variability along the rows and the columns of the field design (Cantet et al., 2005; Cappa and Cantet, 2007; Cappa et al., 2015). The genomic estimated breeding values (GEBVs) for each trait were determined with the genomic best linear unbiased prediction-based model (GBLUP) (Whittaker et al., 2000; Meuwissen et al., 2001; Cantet et al., 2005). The following basic model was considered:


(2) y=μ+Zu+Ws+ε(within the environment)

(3) y=μ+Xβ+Zu+Ws+ε(across environments)

where y is the raw phenotypes; μ the global mean; u the vector of random additive effects following the distribution $N(0,\;G\sigma_{a}^{2}$ ), where $\sigma_{a}^{2}$ is the additive variance and  G the genomic relationship matrix between accessions; s is the vector of random spatial effects containing the parameters of the B-spline tensor product following $N(0,\;S\sigma_{s}^{2}$ ), where $\sigma_{s}^{2}$ is the variance of the RKE for the rows and columns, while S represents the covariance structure in two dimensions; and ε is the vector of residual effects following N (0, $I\sigma_{e}^{2}$ ), where $\sigma_{e}^{2}$ is the residual variance. The design matrices Z and W were identity matrices relating the plot to the random effects. For analyses across environments (*Spain.Global* and *Global*), β (Equation 2) is a fixed effect of the year in the same location or the effect of interaction among locations and X is the design matrix relating the plot to the fixed effect. The method used to obtain the covariance structure S was (Equation 3) the following: bi-splines were anchored at a given number of knots for rows and columns, and a higher number of knots smooths out the surfaces. “*breedR*” optimized the knot numbers by an automated grid search based on the Akaike information criterion (AIC). The microenvironmental individual effect was subtracted from the observed phenotype to obtain a spatially adjusted individual phenotype used to conduct the GWA studies. A genotypic mean of the spatially adjusted phenotypes was calculated for each trait and used for the GWAS. All measurements were tested for deviations from normality by a randomized quantile–quantile (Q–Q) plot.

#### Heritability and correlation

Narrow sense heritability (*h* ^2^) was estimated from the variance components of each model after phenotypic adjustment. For the individual environments (*Spain.2019*, *Spain.2020*, *UK2019*, and *Serbia.2020*), the following formula was used (Equation 4):


(4) $$h^{2}=V_{g}/(V_{g}+V_{sp}+V_{res})$$

(within the environment)

where *V*_g_ is the additive genetic variance component, *V*_sp_ is the spatial variance component, and *V*_res_ is the residual variance component.

To calculate the *h* ^2^ for combined environments (*Spain.Global* and *Global*), the formula used was the following (Equation 5):


(5) $$h^{2}=V_{g}/(V_{g}+V_{spn})+V_{res}/n$$

(across environments)

where *V*_spn_ is the spatial variance component for each environment and *n* is the mean number of replicates (checks) for each accession per environment.

To understand the extent of the relationship between traits and after adjusting phenotypic data, a correlation coefficient analysis was performed using the Pearson method for all the traits across environments. Additionally, the genetic correlation was assessed using a multitrait model on adjusted phenotypes with the “breedR” package (Muñoz and Sanchez, 2020). For each random effect, including genetic and spatial effects, a full covariance matrix is estimated. The “cov2cor” function in the R package “stats” was used to compute the genetic correlation between traits from the additive genetic variance–covariance matrix. This information was based on the research by Calus and Veerkamp (2011). The genetic correlation among traits was extracted and compared with the phenotypic correlation. Descriptive analysis and correlations of the phenotypic data were conducted with the R 4.2.3 software (R Core Team, 2022).

#### Alignment of SNP markers to the *Vicia faba* reference genome

To identify the chromosomal location of SNP markers, their flanking sequences were aligned to the *Vicia faba* reference genome (Jayakodi et al., 2023) using the “map to reference” option implemented in Geneious v.7.1.9. For the genomic position of the SNP markers, the information provided by Skovbjerg et al. (2023) was used. To facilitate data analysis, the extremely large chromosome I (>3 Gbp) was divided at the centromere (position 1,574,527,093 bp) by the faba bean genome consortium to form Chr1S and Chr1L. Functional annotation was done using eggNOG-mapper v.2 with the eukaryotic database (Huerta-Cepas et al., 2019; Cantalapiedra et al., 2021). The associated genes were categorized by the Clusters of Orthologous Group (COG) and plotted using the “ggplot2” R package (Wickham, 2016).

#### Estimation of linkage disequilibrium

To calculate the linkage disequilibrium (LD), only SNP markers with physical position and chromosomal location within the *V. faba* genome were used. So, a genotyping matrix of 19,741 SNP markers was filtered for a MAF of 5% and a numerical imputation with the LD-kNNi method (Money et al., 2015) implemented in TASSEL v5.2.88 (Bradbury et al., 2007). LD was estimated for each chromosome and for the whole genome, by computing the squared allele frequency correlations (*r* ^2^) for each pairwise combination of markers distanced within 1 Mbp in PLINK v.1.9 (Purcell et al., 2007). LD was plotted against the genomic distance between markers in kbp, and a curve was fitted using the LOESS regression model and R. The LD decay was estimated per chromosome and the whole genome as the point where the fitted curve reached half of its maximum value.

#### Population structure and phylogeny

To infer the population structure of the SNP marker panel, a Bayesian-based clustering analysis was performed using fastSTRUCTURE v. 1.0 (Raj et al., 2014). fastSTRUCTURE was run with default settings and 10-fold cross-validation using *K* values ranging from 1 to 10. The most likely number of subpopulations (*K*) was identified by plotting the marginal likelihood of each model as a function of *K* and determining when the graph begins to plateau. The choice of *K* was further supported by applying a discriminant analysis of principal components (DAPC)-based procedure for clustering using the “fviz_pca” function in the “factoextra” R package (Kassambara and Mundt, 2020). The resulting admixture proportions were graphically displayed using the distruct.py script provided by fastSTRUCTURE.

A phylogenetic tree was constructed with the neighbor-joining method applying a bootstrap test with 1,000 replications, using MEGA 11 (Tamura et al., 2021). The R package “ggtree” was then used to visualize a circular phylogenetic tree (Xu et al., 2021, 2022).

#### Genome-wide association analysis

The GWAS analyses were performed using the multi-locus mixed model method (MLMM) (Segura et al., 2012), which accounts for the genetic structure of the faba bean collection within the genomic relationship matrix (*G*), using the R package “mlmm.gwas” (Bonnafous et al., 2019). The MLMM method uses a stepwise mixed-model regression approach with forward inclusion of the SNPs as co-factors and a backward elimination. The variance components of the model were re-estimated at each step (maximum 10 steps) and then used to calculate *p*-values for the association of each SNP with the trait in the study. MLMM implements two model selection methods to determine the optimal mixed model from the set of stepwise models calculated: the extended Bayesian information criterion and the Bonferroni criterion. The Bonferroni method is considered the most stringent for selecting a threshold *p*-value (Kaler and Purcell, 2019) and may result in a loss of power and of true positives. For this reason, in this study, we have considered both the eBIC with a lambda value of 0.60 and the Bonferroni test (0.05 divided by the number of SNPs) as significant cutoffs. Associated markers were visualized with a *p*-value distribution (expected vs. observed on a −log10 scale) with a Manhattan plot and a Q–Q plot. The percentage of phenotypic variation explained by each QTL was obtained by subtracting the *R* ^2^ of a linear model with all the QTL as fixed effects and the genomic relationship matrix (*G*) as random effect to the *R* ^2^ of the same model but without the focused QTL.

The genomic regions (72 bp) harboring associated SNPs for each trait in different environments were represented on the *V. faba* physical map using the Pretzel platform (Keeble-Gagnère et al., 2019) (http://pulses.plantinformatics.io/mapview).

#### Potential candidate gene identification

The genome sequence (Jayakodi et al., 2023) of the associated SNPs was blasted against the NCBI *Medicago truncatula* reference genome (MtrunA17r5.0-ANR) to annotate the potential candidate genes underlying the causal variants. Gene locations were determined using the Genome Data Viewer (GDV).

## Results

### SNP calling

Genotyping of the EUCLEG collection with the Vfaba 60K Axiom array revealed a total of 34,354 SNP markers (57% of the total 59,871 SNPs present on the array) with a call rate above 97%. Following the Axiom Best Practices Genotyping Workflow, these SNPs were classified into three quality classes according to their clustering performance: polymorphic high resolution (PHR, 71%), monomorphic high resolution (MHR, 17%), and no minor homozygous (NMH, 12%). PHR refers to polymorphic SNPs exhibiting all three highly resolved clusters (two homozygous and one heterozygous), MHR to not informative SNPs with only one of the homozygous clusters, and NMH to SNPs with good resolution lacking one of the homozygous clusters. After the quality control, 48 accessions were removed for further analysis, 26 due to poor-quality DNA, and 22 for revealing a low number of SNP markers (7,656 SNPs). The average reference allele frequency was 44%, and for the alternative allele, it was 56%. The number of missing values in the genotyping matrix was low (0.89%). After quality control, the final matrix consisted of 352 accessions genotyped for 22,867 high-quality SNPs (MAF above 5% and without missing data) and was kept for further GWAS analysis.

### Genomic distribution of SNP markers

**Figure 1:**
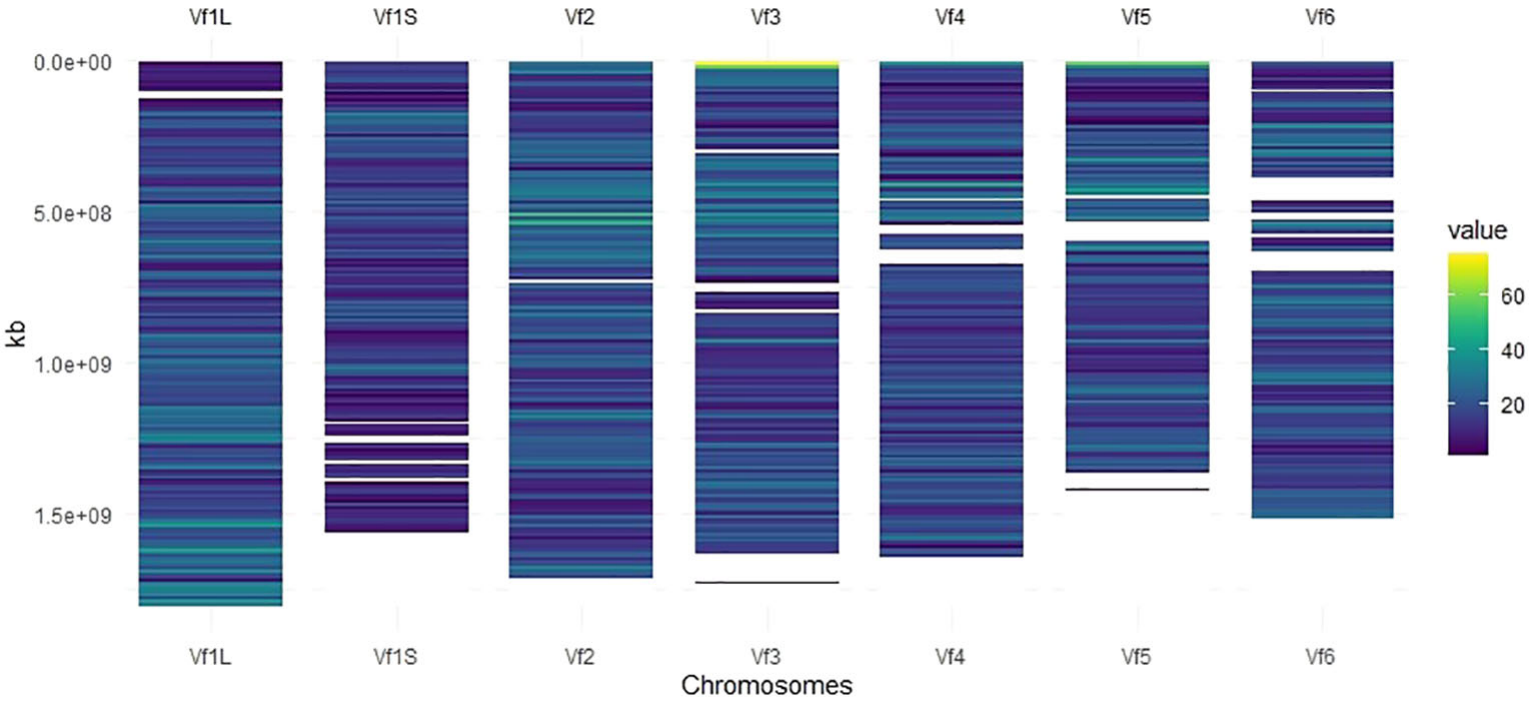
SNP density map across each faba bean chromosome representing the number of SNPs after quality control within a 10-Mbp window size. The color blue corresponds to the lowest density, while the color yellow corresponds to the highest density.

Markers with known chromosomal position, present in a window of 10 Mb, were used to develop the high-density SNP-based map (**Figure 1**). Of the total number of SNP markers (22,867), 93% (21,271) were well-distributed across the six chromosomes after assembling against the faba bean reference genome (Jayakodi et al., 2023), and 19,741 of them (86%) were mapped to a genomic location (**Table 1**). The 1,596 SNP markers not assigned to chromosomes (named as Chr0) together with the ones without genomic positions (1,530 SNPs) were, however, included in the GWAS analysis. The number of SNPs on each chromosome ranged from 3,847 on Chr1L to 2,296 on Chr1S (**Table 1**), and the total genetic coverage was 11.4 Gbp. The highest average density of SNPs was 20 SNPs per 10 Mbp in Chr1L and Chr2 and the lowest was 14 SNPs per 10 Mbp in Chr1S. Chr3 was the one with the maximum local density of SNPs (75 SNPs per 10 Mbp) and the maximum gap between them (95,593.1 kbp). The higher average distance between two adjacent SNPs was 738.5 kbp in Chr1S and the lowest was 506.4 kbp in Chr1L.

**Table 1:** SNP distribution and coverage per individual chromosome of 352 faba bean accessions against the *Vicia faba* reference genome.

Chromosome	SNP number	Length (bp)	SNP density^a^	No. of max. SNPs^b^	SNP distance^c^	Max. gap^d^
**Chr1S**	2,124	1,568,008,521	14	43	738.5	28,576.9
**Chr1L**	3,559	1,804,809,984	20	35	506.4	31,207.6
**Chr2**	3,377	1,716,744,951	20	56	508.5	22,732.4
**Chr3**	3,069	1,733,023,265	18	75	564.8	95,593.1
**Chr4**	2,754	1,645,775,113	17	48	597.4	60,679.1
**Chr5**	2,403	1,429,386,368	17	58	594.8	68,652.8
**Chr6**	2,455	1,519,787,660	16	41	618.9	86,906.2
**Total**	19,741	11,417,535,862				

a Average number of SNPs per 10 Mbp.

b Maximum number of SNPs found on a 10-Mbp window.

c Average spacing between neighboring SNPs in kbp.

d Maximum distance between SNPs in kbp.

### Estimation of linkage disequilibrium

**Figure 2:**
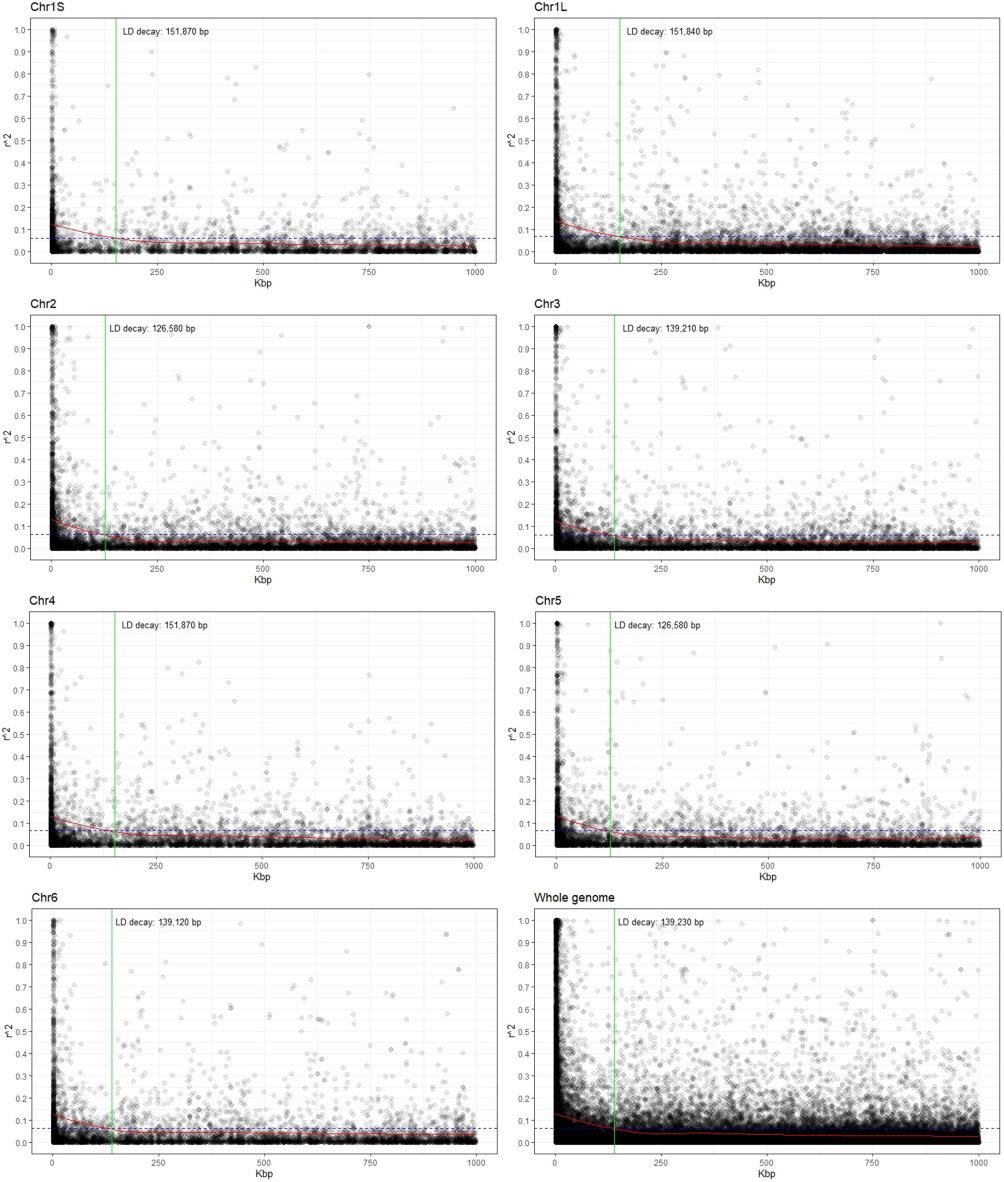
Scatter plot showing the linkage disequilibrium (LD) decay for each chromosome as well as for the whole genome. The values on the *Y*-axis represent the squared correlations of allele frequencies (*r* ^2^) between markers with a maximum distance of 1 Mbp. The *X*-axis shows the genomic distance in kbp. The intersection (green line) between the LOESS curve (red) and the threshold (half of the average value at the minimal distance; dashed blue line) indicates the extent of LD decay in base pairs (bp).

Estimates of the linkage disequilibrium (using *r* ^2^) for each chromosome as well as for the whole genome are presented in **Figure 2**. LD values showed an inverse relationship with distance, and the LD decay, estimated as the distance for which *r* ^2^ decreases to half of its maximum level (0.131), was 139.2 kbp for the whole genome. Considering the chromosomes individually, the *r* ^2^ values ranged from 0.125 in Chr3 to 0.140 for Chr1L and decreased to half at reaching 139.2 kbp and 151.8 kbp, respectively (**Figure 2**; **Supplementary Table S2**).

### Population structure

**Figure 3:**
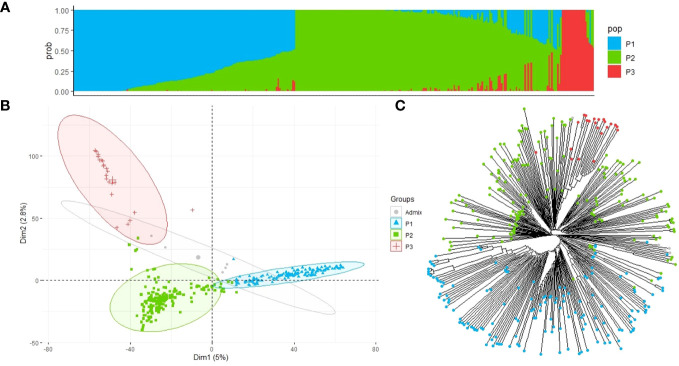
Population structure and principal component analysis of the 352 faba bean accessions. **(A)** Admixture vertical plot at *K* = 3; the vertical bars represent an individual accession, and each color corresponds to its assignment to one of the clusters based on its ancestry proportion. **(B)** Discriminant analysis of principal components (DAPC) for 352 faba bean genotypes revealing three clear groups. Each group is represented by circles with different colors and shapes. **(C)** Neighbor-joining tree of the faba bean accessions. The tips are highlighted with different colors according to the population groups.

We analyzed the population structure of the EUCLEG collection using two approaches: fastSTRUCTURE and DAPC. The marginal likelihood of the fastSTRUCTURE output from *K* = 2 to *K* = 10 was represented, and a delta-*K* peak at *K* = 3 was determined (**Figure 3A**). The accessions were divided into three groups (P1, P2, and P3) with clear differences in geographic origin. Based on the results of the fastSTRUCTURE analysis, accessions with membership probabilities ≥0.50 were considered to belong to the same group. The DAPC supported the choice of *K* = 3 using the first two PCs. PC1 distinguished the population P1 from P2 and P3, whereas PC2 further distinguished P2 and P3 populations (**Figure 3B**).

Group P1 contained 148 accessions, of which 38% had an unknown geographic origin and 44% were associated with North European countries, with Finland (22), Sweden (15), and France (11) being the most highly represented. Population P2 included 177 accessions of which 46% belonged to Mediterranean countries and 26% had an unknown origin. Spain was the country with the highest number of accessions (61), followed by Egypt (9), Ethiopia (7), and Syria (6). P3 was the smallest group, with 22 accessions associated with countries from Asia, with China (15) being the predominant country, followed by Japan (1), Nepal (1), and Thailand (1), and the rest of the accessions (4) were of unknown geographic origin (**Supplementary Table S1**). Five of the 352 accessions were found to be admixed and were not assigned to a specific group.

According to the fastSTRUCTURE approach, the neighbor-joining tree generated with 352 faba bean accessions and 22,867 SNP markers further suggested the three main clades (**Figure 3C**) although populations P2 and P3 did not show such a clear division.

### Phenotypic variation, heritability, and correlation

**Figure 4:**
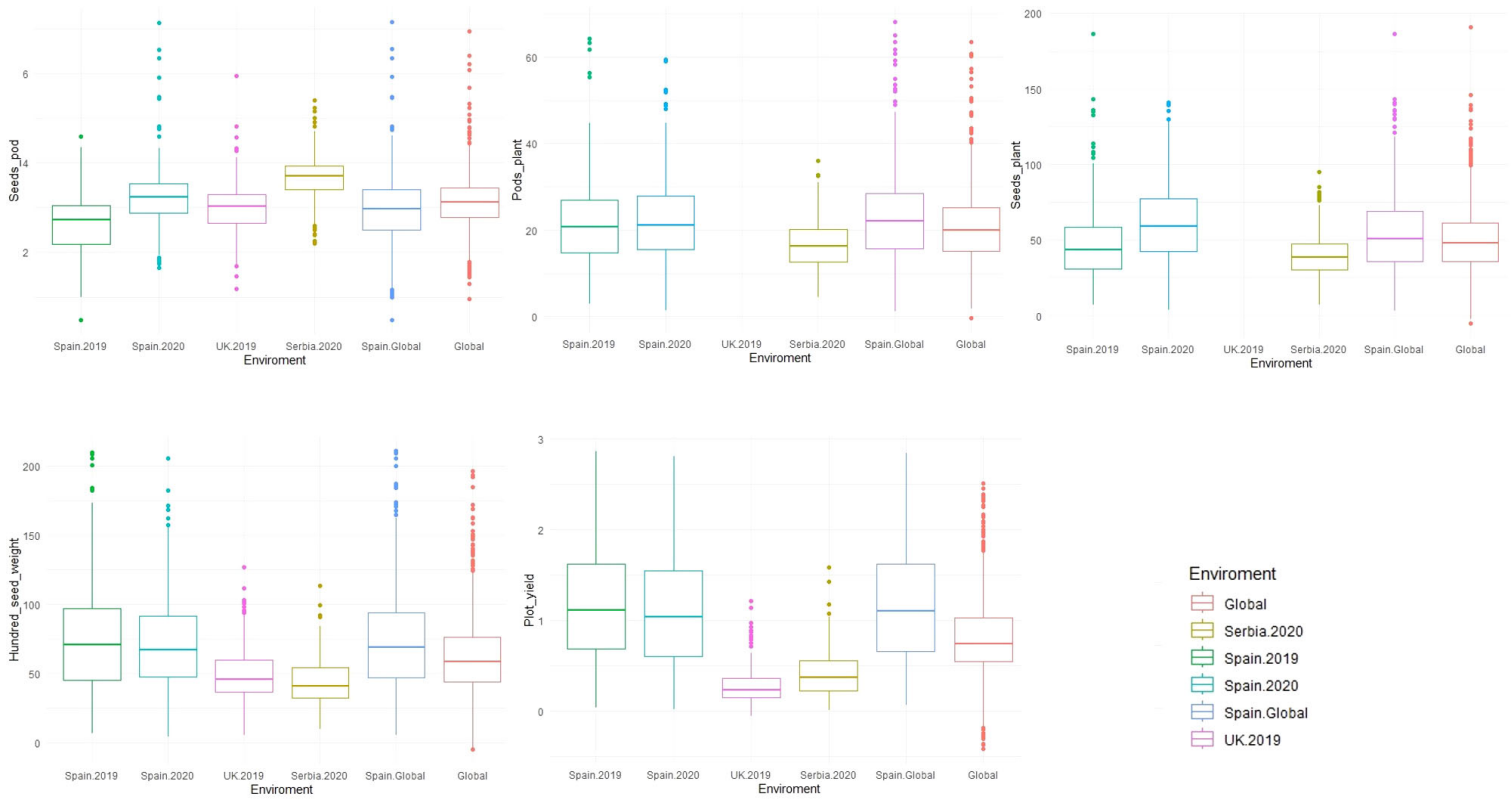
Boxplot of the phenotypic values for five yield-related traits measured in 352 faba bean accessions across different environments (*Spain.2019*, *Spain.2020*, *UK.2019*, *Serbia.2020*, *Spain.Global*, and *Global*). The traits were seeds per pod (SP), pods per plant (PP), seeds per plant (SPL), hundred-seed weight (HSW), and plot yield (PY).

Descriptive analysis, heritability, and correlations of the phenotypic data were estimated after phenotypic adjustment. Except for SH, scored only at *Spain.2019*, all traits showed a high variability across the six environments (**Supplementary Figure S1**) and followed a normal distribution with a positive skewness (except for SH where the deviation of the data was toward indehiscent plants). SP showed the highest phenotypic mean in *Serbia.2020* (3.69 ± 0.50), followed by *Spain.2020* (3.22 ± 0.66). The highest phenotypic mean for PP was in *Spain.Global* [*Spain.2019* (22.70 ± 9.94) and *Spain.2020* (22.97 ± 10.13)], while SPL had a higher mean value in *Spain.2020* (61.08 ± 25.59). HSW showed the highest mean value in *Spain.2019* (74.97 ± 37.97) and *Spain.2020* (71.37 ± 32.77), while the lowest value was in *Serbia.2020* (43.52 ± 16.12). PY revealed the highest mean value in *Spain.Global* [*Spain.2019* (1.18 ± 0.60) and *Spain.2020* (1.15 ± 0.63)], while in Serbia (0.42 ± 0.25) and the UK (0.27 ± 0.19), values were three and four times lower, respectively (**Figure 4**; **Table 2**).

**Table 2:** Descriptive statistics, heritability, and proportion of phenotypic variance explained in each environment after adjusting trait phenotypic values as described in the methods.

Trait	Environments		Mean	SD	Min	Max	Range	*h* ^2^	*varG*	*varS*	*varE*
Shattering (SH)	Spain.2019		1.01	1.31	−0.09	3.08	3.16	0.76	2.583	0.0025	0.8097
Seeds_pod (SP)	Spain.2019		2.56	0.76	0.48	4.59	4.11	0.60	0.6011	0.0013	0.3981
	Spain.2020		3.22	0.66	1.65	7.12	5.48	0.81	0.8039	0.0007	0.1826
	UK.2019		2.97	0.54	1.17	5.93	4.76	0.91	0.6892	0.0004	0.0667
	Serbia.2020		3.69	0.50	2.20	5.40	3.20	0.93	0.6975	0.0007	0.0552
	Spain.Global	Spain.2019	2.56	0.76	0.49	4.60	4.11	0.76	0.5187	0.0117	0.3321
		Spain.2020	3.22	0.66	1.65	7.13	5.48			0.0021	
	Global	Spain.2019	3.09	0.75	0.95	5.07	4.12	0.90	0.4652	0.0003	0.2240
		Spain.2020	3.09	0.65	1.54	6.94	5.39			0.0005	
		UK.2019	3.09	0.54	1.30	6.08	4.78			0.0005	
		Serbia.2020	3.08	0.50	1.60	4.79	3.20			0.0003	
Pods_plant (PP)	Spain.2019		21.57	9.58	2.91	64.29	61.38	0.65	111.2	0.1169	60.460
	Spain.2020		22.44	9.89	1.43	59.33	57.90	0.69	135.3	0.1472	61.300
	Serbia.2020		16.54	5.54	4.55	35.99	31.44	0.94	90.38	0.0702	5.204
	Spain.Global	Spain.2019	22.70	9.94	1.25	68.00	66.75	0.74	107.7	4.9100	67.280
		Spain.2020	22.97	10.13	2.50	61.67	59.17			3.1010	
	Global	Spain.2019	20.69	9.46	1.75	63.49	61.75	0.85	71.25	0.0547	52.570
		Spain.2020	20.67	9.84	−0.39	57.23	57.62			0.1383	
		Serbia.2020	20.70	5.55	8.50	40.24	31.74			0.0867	
Seeds_plant (SPL)	Spain.2019		46.45	24.58	7.24	186.46	179.23	0.65	720.8	1.0360	391.2
	Spain.2020		61.08	25.59	3.53	140.77	137.24	0.67	837.3	0.8801	420.5
	Serbia.2020		39.63	14.93	7.07	95.18	88.11	0.85	426.8	0.5247	76.53
	Spain.Global	Spain.2019	46.56	24.62	7.00	186.25	179.25	0.73	578.2	2.6050	457
		Spain.2020	61.17	25.63	3.33	141.00	137.67			3.0300	
	Global	Spain.2019	49.89	24.48	10.19	190.55	180.36	0.82	377.3	0.5867	369.4
		Spain.2020	49.75	25.34	−5.33	129.08	134.41			0.4953	
		Serbia.2020	49.94	14.95	17.09	105.29	88.20			0.3124	
H_Seed_weight (HSW)	Spain.2019		74.97	37.86	7.23	209.34	202.11	0.91	2,764	2.6	258.3
	Spain.2020		70.89	32.34	4.37	205.17	200.80	0.92	1,996	1.166	177.7
	UK.2019		49.09	18.02	5.81	126.61	120.81	0.98	755.2	0.2728	16.5
	Serbia.2020		43.53	16.12	10.18	113.31	103.12	0.97	518.1	0.2353	13.37
	Spain.Global	Spain.2019	74.97	37.97	6.11	210.44	204.33	0.90	1,670	4.361	380
		Spain.2020	71.37	32.77	5.50	209.72	204.22			13.09	
	Global	Spain.2019	61.52	37.45	−4.75	193.16	197.91	0.91	771.8	0.5101	308.2
		Spain.2020	61.71	32.21	−2.76	195.93	198.70			0.5388	
		UK.2019	61.52	18.05	17.66	139.20	121.54			0.5532	
		Serbia.2020	62.05	16.15	28.54	131.70	103.16			0.3986	
Plot_yield (PY)	Spain.2019		1.15	0.58	0.04	2.86	2.82	0.95	0.6082	0.0002	0.0351
	Spain.2020		1.13	0.62	0.02	2.80	2.79	0.93	0.7990	0.0004	0.0612
	UK.2019		0.27	0.19	−0.05	1.22	1.27	0.71	0.0398	0.0000	0.0165
	Serbia.2020		0.42	0.25	0.02	1.59	1.57	0.87	0.1236	0.0002	0.0185
	Spain.Global	Spain.2019	1.18	0.60	0.09	2.84	2.75	0.83	0.3867	0.0140	0.1257
		Spain.2020	1.15	0.63	0.07	2.83	2.76			0.0113	
	Global	Spain.2019	0.79	0.58	−0.37	2.46	2.82	0.78	0.1135	0.0001	0.1373
		Spain.2020	0.79	0.61	−0.42	2.52	2.93			0.0001	
		UK.2019	0.79	0.19	0.50	1.81	1.31			0.0002	
		Serbia.2020	0.79	0.25	0.38	1.96	1.58			0.0001	

**Figure 5:**
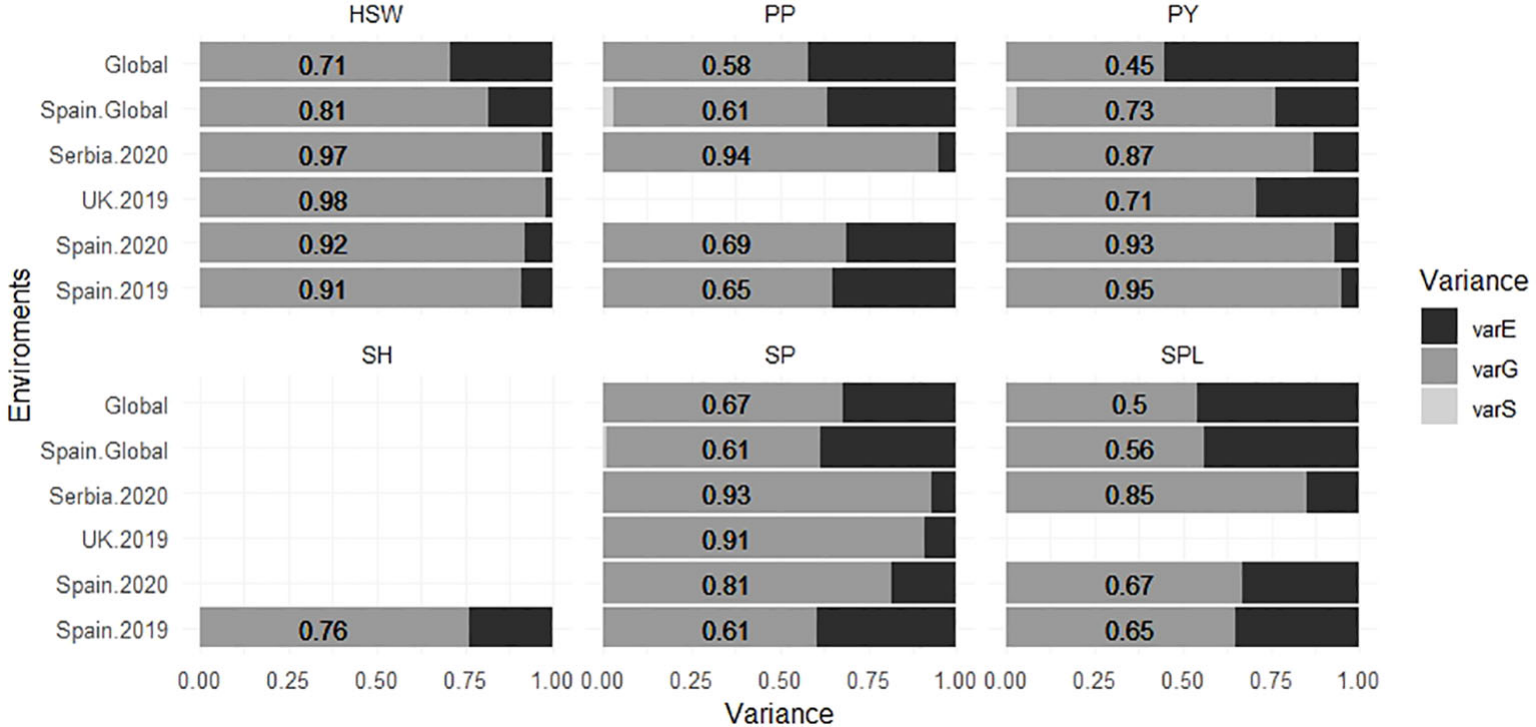
Proportion of the different components of the variance for six faba bean yield-related traits calculated for each environment. The numbers correspond with the value of the *varG*. *varE* is the residual variance component, *varG* is the additive genetic variance component, and *varS* is the spatial variance component for each environment. The traits were SH (shattering), SP (seeds per pod), SPL (seeds per plant), HSW (hundred-seed weight), PP (pods per plant), and PY (plot yield).

The narrow sense heritability (*h* ^2^) values are shown in **Table 2**. On average, the highest heritability values were observed for HSW (0.9 to 0.98), whereas the lowest values were for SPL (0.65 to 0.85). *Serbia.2020* was the location with the highest mean heritability values for SP, PP, and SPL (0.93, 0.94, and 0.85, respectively), while SPL showed the lowest values in *Spain.2019* and *Spain.2020* (0.65 and 0.67). Furthermore, the proportion of the different components of the variance estimated from each environment is represented in **Figure 5**. Overall, the models combining multiple environments (*Global* and *Spain.Global*) displayed higher values for the residual variance component (*varE*).

**Figure 6:**
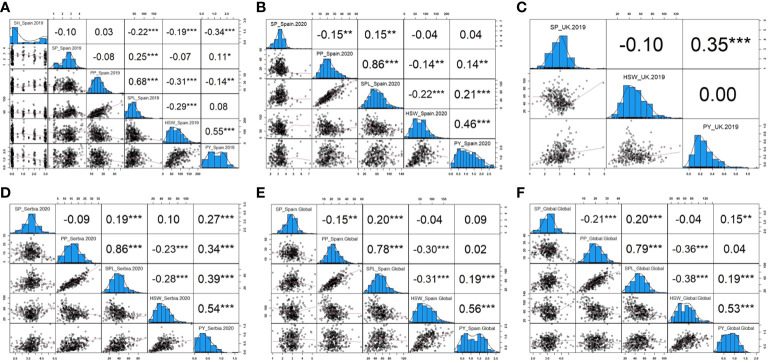
Pearson correlations and histograms showing the distribution of the yield-related traits in each environment. **(A)** Phenotypic frequency and correlation between SH (shattering), SP (seeds per pod), PP (pods per plant), SPL (seeds per plant), HSW (hundred-seed weight), and PY (plot yield) corresponding to Spain in 2019. **(B)** Phenotypic frequency and correlation between SP, PP, SPL, HSW, and PY corresponding to Spain in 2020. **(C)** Phenotypic frequency and correlation between SP, HSW, and PY corresponding to the United Kingdom in 2019. **(D)** Phenotypic frequency and correlation between SP, PP, SPL, HSW, and PY corresponding to Serbia in 2020. **(E)** Phenotypic frequency and correlation between SP, PP, SPL, HSW, and PY corresponding to Spain combined across years. **(F)** Phenotypic frequency and correlation between SP, PP, SPL, HSW, and PY corresponding to the Global analysis combining all locations. *, **, and *** significance at *p<* 0.05, *p<* 0.01, and *p*< 0.001, respectively.

The phenotypic correlation between traits and environments after phenotypic adjustment is shown in **Figure 6**. In general, similar performance patterns were observed in most of the traits across environments. Thus, SH evaluated only in *Spain.2019* revealed a high significance and a negative correlation with SPL, HSW, and PY (−0.22, −0.19, and −0.34, respectively). SP showed a positive correlation with SPL in all environments and a negative correlation with PP in *Spain.2020* (−0.15), *Spain.Global* (−0.15), and *Global* (−0.21). PP displayed a strong positive correlation with SPL and a negative correlation with HSW in all environments, and SPL presented a negative correlation with HSW. Finally, PY revealed a positive correlation with HSW, SPL (except in *Spain.2019*), and PP, although no correlation between PY and PP was detected in *Spain.Global* or *Global* and a negative correlation was observed in *Spain.2019*. Only in *UK.2019*, *Serbia.2020*, and *Global* environments, PY showed a positive correlation with SP (0.35, 0.27, and 0.15, respectively).

The genetic correlation for each trait (**Supplementary Table S3**) was calculated using the data obtained in the different locations, with a genetic correlation close to one indicating a low G × E interaction and a value close to zero indicating a strong G × E interaction. The genetic correlation between traits was estimated between them when all the environmental variation was removed from the phenotype (named “Global”). It was noteworthy that the genetic correlation between SP in *Spain.2019* and in *Serbia.2020* (0.998) was high. Similarly, the correlation of HSW between *Serbia.2019*, *Spain.2019*, and *Spain.2020* varies between 0.928 and 0.950. However, PY was the least correlated trait between the *UK.2019* and the rest of the locations, showing the least (close to zero) correlated trait with *Spain.2020* (−0.049). PP correlations between Serbia and Spain ranged from 0.538 to 0.686, while SPL ranged from 0.571 to 0.776 between those locations. In accordance with the phenotypic correlation (**Figure 6**), PP and SPL showed the highest genetic correlation between them in all environments, and higher values were observed in *Serbia.2020* and *Spain.2020* (0.919 and 0.904, respectively). SH showed a negative genetic correlation with the other traits, while HSW had it with PP, SPL, and SH, as well as PP and SP between them (**Supplementary Table S3**).

### Genome-wide association mapping

Association analyses were performed for the six yield-related traits using individual site data as well as the *Spain.Global* and *Global* values. This identified 112 MTAs in 97 candidate genes, of which 77 had functional annotation (**Table 3**). Of them, 40 harbored markers were significantly associated with the traits [Bonferroni threshold −log10 (*p*) > 5.66]. The Manhattan and their corresponding Q–Q plots are shown in **Supplementary Figure S2**.

**Table 3:** List of candidate genes associated with yield traits in different environments.

Trait	Location	Axiom_Vfaba ID^a^	%R2	Bonf	Chr_Vf	Reference genome Vf	MT_Ortholog	Gene annotation	Mt locations^b^
Shattering (SH)	Spain.2019	AX-416812583	15.4	T	Vf4	4g097400	LOC11444286 - MTR_8g070520	Actin-related protein 8 (ARP)	Chr8: 32,420,418–32,427,339
	Spain.2019	AX-181205104	14.9	T	Vf3	3g118040	LOC11414164 - MTR_1g094730	Protein LYK5	Chr1: 46,228,212–46,230,102
		AX-416731109	12.2	T	Vf4	4g045480	LOC25500825 - MTR_8g028565	WRKY transcription factor 22	Chr8: 10,975,117–10,977,932
		AX-181188996	9.3	F	Vf1L	1g440520	LOC25486300 - MTR_2g029540	30-kDa cleavage and polyadenylation specificity factor 30	Chr2: 11,149,134–11,161,704
		AX-416745732	8.6	F	Vf6	6g023600	LOC11444615 - MTR_4g054920	Cytochrome P450 94C1	Chr4: 27,970,823–27,972,919
		AX-416735297	7	F	Vf3	3g084880	–	–	–
		AX-181148136	6.6	F	Vf2	2g045960	LOC11419536 - MTR_3g055920	Probable cytokinin riboside 5′-monophosphate phosphoribohydrolase LOGL3	Chr3: 25,411,325–25,415,851
		AX-416817919	6.2	F	Vf1L	1g317840	LOC11413965 - MTR_5g038450	Probable serine/threonine-protein kinase PIX7	Chr5: 16,797,826–16,802,888
Seeds_pod (SP)	Spain.2019	AX-416729362	11.2	F	Vf2	2g239680	LOC11421272 - MTR_4g024600	UDP-glucuronate 4-epimerase 3	Chr4: 8,834,452–8,837,002
		AX-181153939	10.4	T	Vf5	5g054520	LOC11408637 - MTR_7g067460	Pentatricopeptide repeat-containing protein At4g28010 (PRP)	Chr7: 31,443,108–31,450,139
		AX-416740100	9.4	T	Vf4	4g124760	LOC120577622	Uncharacterized LOC120577622	Chr8: 36,889,875–36,900,957
		AX-181203726	8.6	T	Vf3	3g080360	LOC11410019 - MTR_1g080340	Histone-lysine N-methyltransferase SUVR4	Chr1: 39,567,264–39,578,478
		AX-416759496	6	F	Vf1L	1g443800	LOC11440205 - MTR_4g108150	14 kDa proline-rich protein DC2.15 (PRP)	Chr4: 52,817,542–52,818,514
		AX-416724190	5.7	F	Vf3	3g038960	LOC120577666	40S ribosomal protein S17-like (RP)	Chr8: 37,485,907–37,486,648
		AX-416790750	4.4	F	Vf1S	1g069440	LOC11416615 - MTR_2g083210	COBRA-like protein 10	Chr2: 41,038,841–41,043,276
	UK.2019	AX-181489146	19.5	T	Vf1S	1g039440	LOC25487826 - MTR_2g094970	Helicase protein MOM1	Chr2: 46,655,993–46,663,142
		AX-181166098	14.3	T	Vf1L	1g227200	LOC11426315 - MTR_5g071550	Probable protein phosphatase 2C 2 (PP2C)	Chr5: 31,700,909–31,703,736
		AX-416778732	13.2	T	Vf1S	1g065120	LOC25487504 - MTR_2g084715	Splicing factor U2af small subunit B	Chr2: 42,015,122–42,019,580
		AX-416816331	7.7	F	Vf3	3g205240	LOC11425698 - MTR_1g012590	UDP-N-acetylglucosamine transporter UGNT1	Chr1: 2,584,219–2,590,273
		AX-181493417	7.3	T	Vf4	4g047120	LOC25500757 - MTR_8g028075	Cytochrome b561 and DOMON domain-containing protein At3g07570	Chr8: 10,468,557–10,475,734
		AX-416732455	6.7	F	Vf1S	1g159880	–	–	–
		AX-416741800	5.7	F	Vf1L	1g387400	LOC11439490 - MTR_5g010000	Endoglucanase	Chr5: 2,586,159–2,589,390
		AX-181438122	4.3	F	Vf2	2g131080	LOC11425191 - MTR_3g091610	Phytolongin Phyl2.2	Chr3: 45,158,382 - 45,160,038
	Spain.Global	AX-181149329	8.1	F	Vf3	3g197400	LOC11423171 - MTR_1g008300	Rhomboid-like protein 11, chloroplastic	Chr1: 881,733–885,768
		AX-416724190	3.5	T	Vf3	3g038960	LOC120577666	40S ribosomal protein S17-like (RP)	Chr8: 37,485,907–37,486,648
	Global	AX-416736976	11.7	F	Vf0	Ung029360	LOC11444270	Rhamnogalacturonate lyase-like (RGL)	Chr8: 30,863,233–30,871,957
		AX-416724190	4.5	T	Vf3	3g038960	LOC120577666	40S ribosomal protein S17-like (RP)	Chr8: 37,485,907–37,486,648
Pods_plant (PP)	Spain.2019	AX-416730393	8.6	F	Vf6	6g200160	LOC11416749 - MTR_8g107380	Protection of telomeres protein 1b (POT1)	Chr8: 49,513,374–49,517,113
		AX-416765420	8	T	Vf4	4g124760	LOC120577622	Uncharacterized LOC120577622	Chr8: 36,889,875–36,900,957
		AX-181163476	7	F	Vf1S	1g090480	LOC11422251 - MTR_6g078300	54S ribosomal protein L37, mitocondrial	Chr6: 36,744,596–36,747,938
		AX-416822980	6.4	F	Vf6	6g138400	LOC11411398 - MTR_8g088150	Carboxyl-terminal-processing peptidase 1, chloroplastic (CTP)	Chr8: 40,577,160–40,583,029
		AX-416807594	5.9	F	Vf3	3g200600	LOC25481877 - MTR_1g009860	Protein ACTIVITY OF BC1 COMPLEX KINASE 1, chloroplastic	Chr1: 1,599,423–1,610,255
		AX-416799595	3.9	F	Vf1L	1g328120	LOC11433776 - MTR_5g043880	Probable WRKY transcription factor 57	Chr5: 19,193,017–19,206,704
	Spain.2020	AX-181460360	10.2	T	Vf1L	1g400320	LOC11437498 - MTR_6g088180	Cleavage stimulation factor subunit 50 (CstF-50)	Chr6: 40,794,459–40,808,637
		AX-416733063	8.8	F	Vf1L	1g346160	LOC11440716 - MTR_4g124030	DNA-directed RNA polymerase I subunit 2	Chr4: 59,293,111–59,319,043
		AX-181439008	7.6	T	Vf1L	1g283160	LOC11423535 - MTR_5g096830	Cytochrome c oxidase assembly protein COX15	Chr5: 43,546,294–43,551,833
		AX-416812479	7.5	T	Vf1S	1g009440	LOC11410541 - MTR_7g010950	Cyclic dof factor 2	Chr7: 3,056,313–3,060,692
		AX-416783339	5.4	F	Vf4	4g181200	LOC11438181 - MTR_4g115970	Pyrophosphate-energized vacuolar membrane proton pump	Chr4: 56,052,505–56,058,129
		AX-416760647	5.4	F	Vf1S	1g123640	LOC25495647 - MTR_6g018920	Floral homeotic protein AGAMOUS	Chr6: 7,501,850–7,503,098
		AX-416775893	4.7	F	Vf1L	1g243680	LOC11408143 - MTR_5g081250	Folylpolyglutamate synthase	Chr5: 36,003,484–36,010,018
	Serbia.2020	AX-416730393	13.2	T	Vf6	6g200160	LOC11416749 - MTR_8g107380	Protection of telomeres protein 1b (POT1)	Chr8: 49,513,374–49,517,113
		AX-416724626	10.6	T	Vf5	5g008520	LOC11436354 - MTR_2g101410	Protein JINGUBANG (JGB)	Chr2: 49,715,905–49,718,673
		AX-416748507	8.5	F	Vf1S	1g078200	LOC25487288 - MTR_2g078540	Uncharacterized LOC25487288	Chr2: 38,871,191–38,875,866
		AX-416739028	7.1	T	Vf5	5g095400	LOC11411488 - MTR_7g088360	Probable protein S-acyltransferase 3	Chr7: 41,196,893–41,199,796
		AX-416763724	6.7	F	Vf1S	1g069400	LOC11415615 - MTR_2g083220	Rab GTPase-activating protein 22	Chr2: 41,042,418–41,049,391
		AX-416754106	5.8	F	Vf3	3g238080	LOC11437395 - MTR_3g114030	Thaumatin-like protein 1b	Chr3: 56,596,596–56,602,725
		AX-416793421	4.4	F	Vf1S	1g017520	LOC25497623 - MTR_7g017900	6-phosphogluconate dehydrogenase, decarboxylating 2	Chr7: 6,009,237–6,013,808
	Spain.Global	AX-416765420	11.9	T	Vf4	4g124760	LOC120577622	Uncharacterized LOC120577622	Chr8: 36,889,875–36,900,957
		AX-416788562	8.3	T	Vf4	4g204800	LOC11418607 - MTR_4g124560	DNA repair protein RAD51 homolog	Chr4: 59,677,804–59,681,527
		AX-416777278	8.1	T	Vf5	5g112120	–	–	–
		AX-416772251	7.8	T	Vf2	2g084120	LOC25489423 - MTR_3g074050	Phosphatidylinositol transfer protein 3	Chr3: 36,901,813–36,904,344
		AX-181163860	6.7	F	Vf5	5g030840	LOC11420563 - MTR_7g058430	E3 ubiquitin-protein ligase PRT1	Chr7: 27,468,773–27,476,588
		AX-181497612	6.3	F	Vf2	2g241320	LOC11423056 - MTR_4g023560	ATP-dependent DNA helicase 2 subunit KU80	Chr4: 8,492,295–8,500,374
		AX-416822980	6.3	F	Vf6	6g138400	LOC11411398 - MTR_8g088150	Carboxyl-terminal-processing peptidase 1, chloroplastic (CTP)	–
		AX-181189847	4.9	F	Vf4	4g231800	LOC11435791 - MTR_4g132340	U3 small nucleolar RNA-associated protein 6 homolog	Chr8: 40,577,160–40,583,029
	Global	AX-181439008	6.2	F	Vf1L	1g283160	LOC11423535 - MTR_5g096830	Cytochrome c oxidase assembly protein COX15	Chr5: 43,546,294–43,551,833
		AX-416765420	6.1	F	Vf4	4g124760	LOC120577622	Uncharacterized LOC120577622	Chr8: 36,889,875–36,900,957
		AX-181163860	5.9	F	Vf5	5g030840	LOC11420563 - MTR_7g058430	E3 ubiquitin-protein ligase PRT1	Chr7: 27,468,773–27,476,588
		AX-416783339	4.8	F	Vf4	4g181200	LOC11438181 - MTR_4g115970	Pyrophosphate-energized vacuolar membrane proton pump	Chr4: 56,052,505–56,058,129
Seeds_plant (SPL)	Spain.2019	AX-416783780	7.8	T	Vf0	–	–	–	–
		AX-416757363	7.7	T	Vf2	–	–	–	–
		AX-416745161	7.4	T	Vf5	5g082040	LOC11429113 - MTR_7g083720	V-type proton ATPase subunit C	Chr7: 38,993,699–39,001,736
		AX-181186344	7.2	F	Vf6	6g117120	LOC25493041 - MTR_4g087905	Nudix hydrolase 15, mitocondrial	Chr4: 42,551,506–42,555,292
		AX-181205104	6.9	T	Vf3	3g118040	LOC11414164 - MTR_1g094730	Protein LYK5	Chr1: 46,228,212–46,230,102
		AX-181148593	6.4	F	Vf2	2g002120	LOC11416313 - MTR_3g010180	GPI-anchored protein LLG1 (GPI-AP)	Chr3: 2,335,419–2,337,669
		AX-416739501	4.6	F	Vf4	4g214600	LOC11440584 - MTR_2g036860	Villin-4	Chr2: 16,084,673–16,101,032
	Spain.2020	AX-181460360	12.6	T	Vf1L	1g400320	LOC11437498 - MTR_6g088180	Cleavage stimulation factor subunit 50 (CstF-50)	Chr6: 40,794,459–40,808,637
		AX-181147789	8.7	T	Vf3	3g220120	LOC11431072 - MTR_1g018320	Strigolactone esterase RMS3	Chr1: 5,255,290–5,256,875
		AX-416761617	8.6	F	Vf4	4g180280	LOC11442878 - MTR_4g115640	Putative chloride channel-like protein CLC-g	Chr4: 55,897,598–55,903,091
		AX-181204824	7	F	Vf1L	1g340840	LOC11407099 - MTR_5g026910	Uncharacterized LOC11407099	Chr5: 11,164,632–11,168,640
		AX-416794410	6.6	F	Vf5	–	–	–	–
		AX-416790652	5.2	F	Vf3	3g184840	LOC11421623 - MTR_1g031780	Putative receptor-like protein kinase At4g00960 (RLK)	Chr1: 11,241,206–11,247,464
		AX-416788562	5	F	Vf4	4g204800	LOC11418607 - MTR_4g124560	DNA repair protein RAD51 homolog	Chr4: 59,677,804–59,681,527
		AX-416781866	4.8	F	Vf6	6g049840	LOC11440200 - MTR_4g071880	Fructose-bisphosphate aldolase 1, chloroplastic (FBA)	Chr4: 35,238,383–35,247,955
	Serbia.2020	AX-416724626	9.2	T	Vf5	5g008520	LOC11436354 - MTR_2g101410	Protein JINGUBANG (JGB)	Chr2: 49,715,905–49,718,673
		AX-181493754	8.6	F	Vf6	6g046000	LOC25492796 - MTR_4g073840	Putative disease resistance RPP13-like protein 3	Chr4: 36,019,614–36,023,252
		AX-416817126	8.2	F	Vf2	2g090040	LOC11413114 - MTR_3g077080	Copper methylamine oxidase	Chr3: 38,099,287–38,107,913
		AX-416730393	8	T	Vf6	6g200160	LOC11416749 - MTR_8g107380	Protection of telomeres protein 1b (POT1)	Chr8: 49,513,374–49,517,113
		AX-416752104	7.3	F	Vf1S	1g198960	LOC11417190 - MTR_1g018030	Serotonin N-acetyltransferase 2, chloroplastic	Chr1: 5,212,095–5,214,935
		AX-181204029	6.8	F	Vf6	6g091000	LOC11437903 - MTR_4g096900	Aspartate carbamoyltransferase 3, chloroplastic (ATCase)	Chr4: 46,486,489–46,491,286
		AX-416778114	5.2	F	Vf3	3g080280	LOC11407475 - MTR_1g080320	Uncharacterized LOC11407475	Chr1: 39,552,506–39,557,764
H_seed_weight (HSW)	Spain.2020	AX-416773275	16.8	F	Vf6	6g042880	LOC25492681 - MTR_4g068457	Protein JINGUBANG (JGB)	Chr4: 33,844,582–33,847,451
		AX-181448659	10.3	F	Vf4	4g151200	LOC11412050 - MTR_4g104350	Protein STICHEL-like 2	Chr4: 51,062,681–51,069,264
		AX-416722650	6.8	F	Vf4	4g114560	LOC11408165 - MTR_8g076150	V-type proton ATPase 16 kDa proteolipid subunit	Chr8: 34,714,373–34,716,329
		AX-416816211	2.7	F	Vf5	5g181520	LOC11430562 - MTR_7g113920	Phosphoglycerate mutase-like protein AT74H	Chr7: 54,000,124–54,003,242
	UK.2019	AX-416789762	16.9	T	Vf1L	–	–	–	–
	Serbia.2020	AX-416792429	15.8	T	Vf5	–	–	–	–
		AX-181497672	14	F	Vf1L	1g225400	LOC11405494-MTR_5g032380	Ubiquitin carboxyl-terminal hydrolase 17	Chr5: 13,805,006–13,815,781
	Global	AX-181197475	20.5	T	Vf1L	–	LOC11436753 - MTR_5g009700	Hydroxyproline O-galactosyltransferase HPGT1	Chr5: 2,415,935–2,423,973
		AX-416789762	15.9	T	Vf1L	–	–	–	–
		AX-181483657	15.2	F	Vf1L	1g441080	LOC11414408-MTR_2g029040	BRISC and BRCA1-A complex member 2	Chr2: 10,878,648–10,887,239
		AX-416813674	12.7	T	Vf1S	–	–	–	–
		AX-181471800	7.3	F	Vf4	4g000600	LOC11435395-MTR_8g005270	Mannan endo-1,4-beta-mannosidase 6	Chr8: 230,980–234,251
		AX-416816053	5.5	F	Vf3	3g171360	LOC25485595-MTR_1g112320	UPF0307 protein PMI3641	Chr1: 54,554,225–54,559,444
		AX-181187483	3.9	F	Vf2	2g269560	–	–	–
		AX-416761691	2.8	F	Vf5	5g039080	LOC25498271-MTR_7g056233	Oleoyl-acyl carrier protein thioesterase 1, chloroplastic	Chr7: 26,103,028–26,112,036
Plot_yield (PY)	Spain.2019	AX-416744497	21.9	T	Vf2	–	–	–	–
		AX-416760651	19.8	T	Vf1S	1g089640	LOC11422248 - MTR_6g077860	DNA-binding protein ROOT HAIRLESS 1 gene (RHL1)	Chr6: 36,585,521–36,596,508
		AX-181494841	14.5	T	Vf1S	1g043400	LOC11417361 - MTR_2g093100	Uncharacterized LOC11417361	Chr2: 45,759,498–45,770,253
		AX-181459267	12.1	F	Vf4	4g161640	LOC11440572 - MTR_4g107830	Hypersensitive-induced response protein 1 (HIR1)	Chr4: 52,667,925–52,682,756
		AX-416814439	12	F	Vf4	4g180440	LOC11446673 - MTR_4g115620	Probable sucrose-phosphate synthase (SPS)	Chr4: 55,872,671–55,880,769
		AX-416824665	10	T	Vf1S	1g119680	LOC11418954 - MTR_6g023340	Basic endochitinase	Chr6: 8,380,403–8,381,786
		AX-416801028	8.3	F	Vf3	3g038240	LOC25483703 - MTR_1g057150	Protein SMAX1-LIKE 3	Chr1: 29,007,041–29,012,213
		AX-416751279	7.9	F	Vf6	6g199840	LOC11415767 - MTR_8g107250	Tubulin beta chain	Chr8: 49,453,661–49,456,244
	UK.2019	AX-416786350	14.5	T	Vf1L	1g079040	LOC11442148 - MTR_5g080260	Uncharacterized LOC11442148	Chr5: 35,537,265–35,538,015
		AX-416803636	10.8	F	Vf2	2g127080	LOC11427826 - MTR_3g090480	LRR receptor-like serine/threonine-protein kinase RCH1	Chr3: 44,517,008–44,521,950
		AX-181440082	10.4	T	Vf5	5g039640	LOC11436813 - MTR_7g046250	E3 ubiquitin-protein ligase BRE1-like 1	Chr7: 22,277,729–22,300,671
		AX-416749659	8.9	F	Vf2	2g274360	LOC25491241 - MTR_4g008150	Metal tolerance protein C4	Chr4: 1,626,201–1,632,456
		AX-416739576	7.7	F	Vf6	6g038240	LOC11427645 - MTR_4g064740	Serine/arginine repetitive matrix protein 1	Chr4: 32,339,258–32,347,116
		AX-416740534	3.6	F	Vf1L	1g306120	LOC11413521 - MTR_5g033800	Protein trichome birefringence-like 2	Chr5: 14,494,614–14,500,404
		AX-181485567	3.2	F	Vf4	4g125640	LOC25493470 - MTR_4g094848	Filament-like plant protein 7	Chr4: 47,043,537–47,050,784
		AX-181456171	2.5	F	Vf3	–	–	–	–

The ID of the SNP markers in the *Vicia faba* Axiom, percentage of phenotypic variation explained (%R2) and Bonferroni threshold (T: true and F: false), location of the SNP in the faba bean chromosomes, contig in the reference genome and orthologous genes, annotation, and location in Medicago truncatula.

a In gray, the common associated loci among traits across environments.

b Gene locations were determined using the Genome Data Viewer (GDV).

A total of 8, 17, 25, 17, 14, and 16 unique MTAs were detected for SH, SP, PP, SPL, HSW, and PY, respectively (**Table 3**; **Supplementary Figure S2**). Among the associated SNP markers, eight were detected and validated in *Global* environments or in at least two environments [AX-181439008 (Chr1L), AX-416789762 (Chr1L), AX-416724190 (Chr3), AX-416783339 (Chr4), AX-416765420 (Chr4), AX-181163860 (Chr5), AX-416822980 (Chr6), AX-416730393 (Chr6)], and five markers were found to be associated with multiple traits (**Supplementary Table S4**). Thus, markers AX-181460360 (Chr1L), AX-416788562 (Chr4), AX-416724626 (Chr5), and AX-416730393 (Chr6) were associated with both PP and SPL, which showed the highest phenotypic correlation in all environments, and AX-181205104 (Chr3) was significant for SPL and SH, which revealed a reasonable high phenotypic correlation as well. No common associated markers were identified for PY, while PP was the trait sharing more markers in different environments or with correlated traits, and all of them were further significant in the global analyses (**Table 3**; **Supplementary Table S4**).

Among environments, *Spain.2019* was the one revealing the highest number of associations for all the traits, except HSW (**Table 3**). Thus, the eight MTAs found for both SH and PY jointly explained 80.2% and 100% of the total phenotypic variation, respectively. In *Spain.2020*, seven, eight, and four MTAs accounted for 49.6%, 58.5%, and 36.6% of the variation for PP, SPL and HSW, respectively. The MTAs detected in *Serbia.2020* explained 56.3%, 53.3%, and 29.8% of the variation in PP, SPL and HSW, respectively. Finally, in *UK.2019*, the markers associated with SP, PY, and HSW accounted for 78.7%, 61.6%, and 16.9% of the respective trait variation. The global analyses *Spain.Global* or the combination of all the environments *Global* identified new candidates and confirmed some of the MTAs previously detected (**Table 3**).

**Figure 7:**
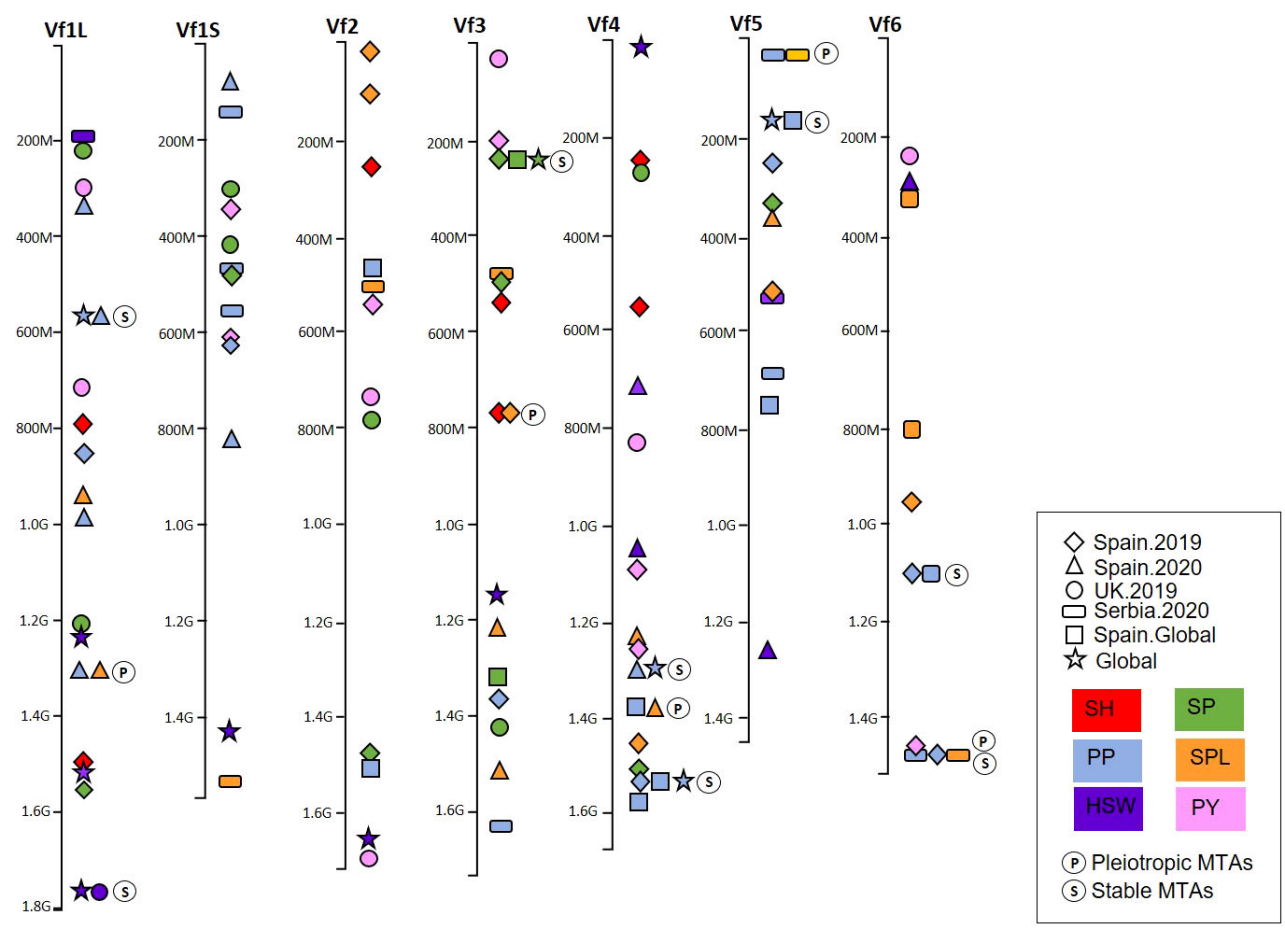
Physical map of the marker–trait associations (MTAs) of six yield-related traits detected by GWAS in a faba bean panel (352 accessions). The traits were represented by colors and the environments by shapes. (P) represents the MTAs with pleiotropic effect and (S) the MTAs stable across different environments. SH, shattering; SP, seeds per pod; PP, pods per pod; SPL, seeds per plant; HSW, hundred-seed weight; PY, plot yield.

The recent availability of the genome sequence (Jayakodi et al., 2023) enabled us to locate the significant MTAs on the faba bean physical map and visualize the genomic regions harboring multiple associations for the different traits and environments (**Figure 7**; **Supplementary Table S5**). The MTAs were distributed across the genome, but a few genomic regions harbored multiple associated SNPs (**Figure 7**). Four pleiotropic MTAs controlling multiple traits co-localized on Chr1L (PP-SPL), Chr3 (SH-SPL), Chr4 (PP-SPL), and Chr5 (PP-SPL). Seven stable MTAs expressed in multiple environments were identified for PP in Chr1L, Chr4, Chr5, and Chr6; for HSW in Chr1L; and SP in Chr3. Finally, we found one MTA showing pleiotropic effects between PP and SPL and stable among environments on Chr6 (**Figure 7**). The nucleotide sequence of each associated SNP is available in **Supplementary Table S6**.

### Postulation of candidate genes

**Figure 8:**
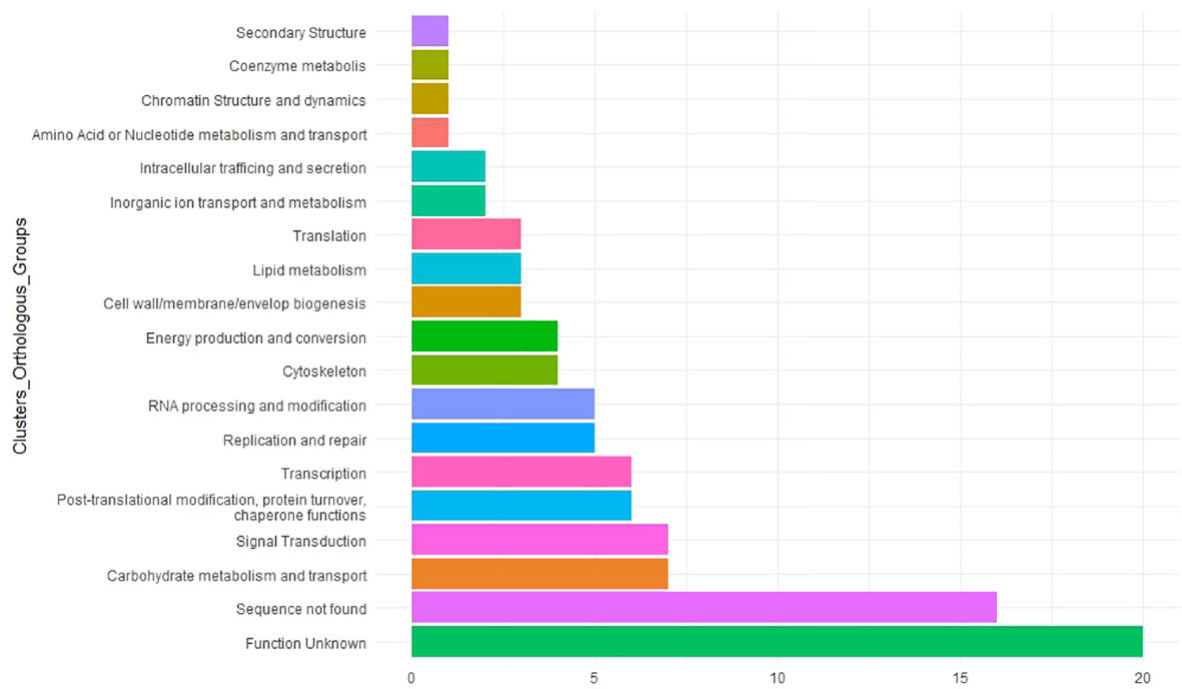
Functional analysis of 97 significant marker–trait associations (MTAs). Candidate genes were classified by the Clusters of Orthologous Groups (COGs). The *X*-axis indicates the number of genes in a category. The *Y*-axis shows the 17 functional COG categories found.

To understand the potential role of MTAs in faba bean yield, we searched for homologs in *M. truncatula* (**Table 3**). Functional annotation of their molecular functions helps to predict candidate genes associated with the traits studied. The COG showed that 20 of them were unknown and 16 of them had no significant similarity with previous annotated sequences (**Figure 8**; **Supplementary Table S7**). The remaining candidates were involved in a wide variety of functions such as carbohydrate metabolism and transport, signal transduction, chaperone functions, transcription, replication and repair process, and cytoskeleton (**Figure 8**).

For shattering (SH), three significant MTAs were identified accounting for 15.4%, 14.9%, and 12.2% of the phenotypic variation, respectively. The candidate genes harboring the significant SNPs were “actin-related protein 8 (ARP),” “protein LYK5,” and “WRKY transcription factor 22” (**Table 3**).

For seeds per pod (SP), the two main candidate genes containing the associated MTAs identified in *Spain.2019* were “UDP-glucuronate 4-epimerase 3” and “pentatricopeptide repeat-containing (PPR) protein” which explained 11.2% and 10.4% of the phenotypic variation, respectively. In *UK.2019*, “helicase protein MOM1,” “protein phosphatase 2C 2 (PP2C),” and “splicing factor U2af small subunit B” harbored significant SNPs that explained 19.5%, 14.3%, and 13.2% of the trait variation, respectively. Two MTAs corresponding to “rhomboid-like protein” and “40S ribosomal protein S17-like (RP)” were also associated with *Spain.Global* and accounted for 8.1% and 3.5% of the phenotypic variation, respectively. Finally, the *Global* analysis identified MTAs corresponding to “rhamnogalacturonate lyase-like gene (RGL)” in addition to “40S ribosomal protein S17-like (RP)” (**Table 3**).

Pods per plant (PP) was the trait sharing the highest number of associated SNPs across environments and yield-related traits (SP), and most of the corresponding candidates were further significant in the global analyses (**Table 3**). Thus, “protection of telomeres protein 1b” (POT1) was associated with PP in *Spain.2019* and with PP and SP in *Serbia.2020* and was the significant marker explaining the highest percentage of variation (13.2%) of the trait. The MTA identified in the uncharacterized LOC120577622 was significant in *Spain.2019*, *Spain.Global*, and *Global*, explaining 8%, 11.9%, and 6.1% of the variation in PP, respectively, a fact supported by the high positive phenotypic correlations between environments (*r* > 0.76) (**Supplementary Figure S3**). Similarly, the significant MTA present in “carboxyl-terminal peptidase (CTP)” was consistently associated with PP in *Spain.2019* and *Spain.Global*. Moreover, “cleavage stimulation factor subunit 50” (CstF-50) contained a significant MTA for PP and SPL that explained 10.2% and 12.6% of the variation, respectively. This outcome was further supported by the high positive phenotypic correlations existing between both traits (*r* = 0.86) (**Figure 6**). “Protein JINGUBANG” (JGB) also contained a significant MTA significant for PP and SPL that explained 10.6% and 9.2% of the variation, respectively. In *Spain.Global*, four associated MTAs uncovered new candidates, an uncharacterized gene was further confirmed in the *Global* analysis, “DNA repair protein RAD51 homolog” was consistently identified in PP and SP, and “E3 ubiquitin-protein ligase PRT1” was further validated in the *Global* analysis. As mentioned above, the pooled dataset analysis (*Global*) validated the genes harboring the associated SNPs (COX15, uncharacterized LOC120577622, E3 ubiquitin-ligase, and vacuolar proton pyrophosphatase) in all the environments.

For seeds per plant (SPL), the five candidate genes harboring significant MTAs consistently identified in different yield-related traits were LYK5 (*Spain.2019* for SPL and SH), CstF-5 and RAD51 (*Spain.2020* for SPL and PP), and JGB and POT1 (*Serbia.2020* for SPL and PP). Except for CstF-50, none of the MATs accounted for more than 10% of the phenotypic variation of this trait (**Table 3**).

In hundred-seed weight (HSW), five of the associated SNP markers corresponded to candidate gene sequences with no significant similarity and accounted for high values of the phenotypic variation (one of them common in the *UK.2019* and *Global* analyses). In *Spain.2020*, the candidate genes were “JGB” protein (different from the one detected in PP and SPL) and “protein STICHEL-like 2” and explained 16.8% and 10.3% of the total variation. In *Serbia.2020*, “ubiquitin carboxyl-terminal hydrolase 17” explained 14% of the trait variation. The associated SNPs in the *Global* analysis revealed “hydroxyproline O-galactosyltransferase HPGT1” accounting for 20.5% of the variation and BRISC and BRCA1-A complex member 2 (explaining 15.2%) (**Table 3**).

Plot yield (PY) was the only trait that did not share candidate genes among environments or with other correlated traits. In *Spain.2019*, six significant MTAs explained more than 90% of the phenotypic variation and corresponded to “DNA-binding protein ROOT HAIRLESS 1 gene (RHL1),” “hypersensitive-induced response protein 1 (HIR1),” “sucrose-phosphate synthase (SPS),” and “basic endochitinase.” In *UK.2019*, the study revealed three main candidates: one of them uncharacterized, “LRR receptor-like serine/threonine-protein kinases RCH1,” and “E3 ubiquitin-protein ligase BRE1-like 1,” and the associated SNPs explained 14.5%, 10.8%, and 10.4% of the variation (**Table 3**). The remaining candidates have not yet been functionally characterized.

## Discussion

Faba bean is an important crop for global food security, ecosystem resilience, and sustainable agriculture. To turn this crop into an economically attractive proposition for farmers and to increase the area under cultivation, major advances in yield and yield stability need to be achieved. Grain yield, however, is a complex trait controlled by many genes and strongly affected by the environment. Genetic improvement can be achieved, but the difficulty is in knowing which trait combinations should be selected to produce stable high-yielding genotypes. This study is a comprehensive effort to exploit a wide collection of important yield-related traits. We performed a GWAS analysis on a world collection of 400 faba bean accessions to assess its genetic diversity and population structure and to identify MTAs associated with yield using the Vfaba_v2 Axiom 60K SNP array. The panel was phenotyped for six yield-related traits (SH, SP, PP, SPL, HSW, and PY) in different locations from Spain, Serbia, and the United Kingdom. For phenotypic adjustment, we considered six models (*Spain.2019*, *Spain.2020*, *Spain.Global*, *UK.2019*, *Serbia.2020*, and *Global*). Overall, there was better performance of the genotypes in Spain than in Serbia or the UK, with better phenological development and greater range for all traits.

The high and positive phenotypic correlations detected between most of the traits in the different locations suggest that it is possible to improve multiple yield-related traits through genomics-assisted breeding. The exception was HSW, which was positively correlated with PY and negatively correlated with PP and SPL in all the environments. This outcome could be the result of genetic and/or environmental interactions with the availability and remobilization of photoassimilates during seed filling (Mangini et al., 2018). However, this explanation does not exclude the hypothesis of clusters of tightly linked genes with opposite effects and/or single genes with pleiotropic effects. On the other hand, the relatively low genetic correlation observed in PY among environments highlights a high G × E interaction with a significant effect on the final yield which is not shared with the other yield components studied. Thus, the bimodal distribution of PY in Spain (**Supplementary Figure S1**) suggests that the MTAs detected might differentiate between Mediterranean-adapted and not-adapted genotypes and could be related to other adaptive loci that respond to environmental factors. These outcomes highlight the fact that identifying MTAs for yield components offers more scope for future breeding than those associated with PY itself.

A high heritability was observed for all the traits in all environments, suggesting a strong genetic control and low microenvironmental effects, a fact desired for rapid progress in breeding. HSW was the least affected trait across environmental conditions, followed by PY and SP. Different studies have reported high narrow sense heritability for SP, PP, HSW, and PY (Toker, 2004; Zhou et al., 2021; Khan et al., 2022; Singh et al., 2022). Traits with high heritability will help breeders shorten the breeding cycles and can result in faster and higher genetic gain (Singh et al., 2022).

Based on the results of the population structure, the delta-*K* peak suggested the presence of three faba bean groups with clear differences in their geographic origin which was supported by the DAPC and the phylogenetic analysis. Three subpopulations differentiated by geographical origin were also found recently in a genetic analysis of a worldwide collection of 2,678 faba bean genotypes, including the EUCLEG panel (Skovbjerg et al., 2023) which also identified the EUCLEG collection being the most diverse among the panels analyzed. As indicated by the diagonal linear shape in the Q–Q plots (**Supplementary Figure S2**), the approach used controlled the population stratification, thus supporting the reliability of the GWAS detected. This collection with diverse phenotypic and molecular parameters constitutes a valuable resource for future breeding and high-resolution gene mapping, including candidate gene discovery for a wide range of traits (Govindaraj et al., 2015).

The adequacy of association studies for complex traits depends critically on the existence of LD between functional alleles and the surrounding SNP markers. LD values dropped from 0.140 to 0.125, with the increase of physical distance from 126.6 kbp to 151.8 kbp. Low LD in faba bean was previously reported (Skovbjerg et al., 2023; Zhang et al., 2023) when comparing different faba bean diversity panels. Thus, the EUCLEG collection was the one showing lower LD blocks (higher recombination), an expected outcome in outbreeding species with high genetic diversity (Pégard et al., 2023; Skovbjerg et al., 2023; Zanotto et al., 2023; Zhang et al., 2023). The rapid LD decay observed in this study (within roughly 150 kbp) revealed the variation present in a highly allogamous panmictic population and suggested that the MTAs identified (or the closely linked genes on either flanking side of the significant SNPs) were nearly or completely independent from each other and, hence, were sufficient for association mapping in faba bean.

Concerning the candidate genes harboring the significant SNPs, we will mainly discuss the ones detected consistently in different environments and traits (stable and pleiotropic MTAs), explaining more than 10% of the trait variation (**Table 3**). Three main candidates were associated with shattering (SH): “actin-related protein 8 (ARP),” “protein LYK5,” and “WRKY transcription factor 22.” No single specific role has been defined for nuclear ARPs that are involved in many cellular physiological processes including plant growth and development (Szymanski and Staiger, 2018). Of particular interest is the role of actin as a sensor mechanism for chemical and physical perturbations in the intracellular and extracellular environment (Porter and Day, 2016), as what may happen in mature faba bean pods. The protein LYK5 has been reported to be involved in cell wall integrity maintenance mechanisms (Baez et al., 2022). In the signaling response of *Arabidopsis* to fungal chitin, the LYK5 plays a direct role in chitin signaling and plant innate immunity (Bacete and Hamann, 2020). Interestingly, LYK5 was pleiotropic with the correlated trait SPL. A WRKY transcription factor has been reported to modulate floral and seed development, lignin deposition, and shattering process in sorghum (Tang et al., 2013; Ogutcen et al., 2018). In our study, the protein LYK5 showed a pleiotropic effect with SH and SPL in *Spain.2019*, supported by the significant negative correlation between SH and SPL in this environment (*r* = −0.22), thus suggesting the importance of this candidate for yield improvement through marker-assisted selection (MAS).

The seeds per pod (SP) main candidates bearing the associated MTAs were “UDP-glucuronate 4-epimerase 3” and “pentatricopeptide repeat-containing (PPR) protein.” Several authors (Duan et al., 2022; Li et al., 2022) reported that gene seed thickness 1 (ST1), encoding UDP-d-glucuronate 4-epimerase 6, influences soybean seed morphology via the pectin biosynthesis pathway. In addition, genes encoding PPR proteins have been shown to play prominent roles in seed development in different crops. Thus, a PPR protein was partly responsible for the increased seed size and weight during domestication in peanut (Li et al., 2021), and it affected photosynthesis and grain filling in maize (Liu et al., 2013; Huang et al., 2020) and regulated pod number in chickpea (Das et al., 2016). Other significant MTAs, although explaining less percentage of variation, corresponded to “histone-lysine N-methyltransferase SUVR4” with functions in plant growth and development, including pollen and female gametophyte development, flowering, and responses to stresses. The other three significant MTAs for SP corresponded to “helicase protein MOM1,” “protein phosphatase 2C 2 (PP2C),” and “splicing factor U2af small subunit B.” The *in-vivo* role of many helicases has not been well investigated in plants; however, through indirect evidence, it has been suggested that they play critical roles in biological pathways encompassing all aspects of cell biology, organismal physiology, development, and stress physiology (Sami et al., 2021). Plant PP2Cs have emerged as major players in stress signaling. A PP2C is a major signaling component in the ABA-dependent signaling cascade that regulates seed germination in rice (Bhatnagar et al., 2017) or seed dormancy abscisic and abscisic acid-activated protein kinases in *Arabidopsi*s (Kim et al., 2013). The regulation of MAPK activities by PP2Cs in plants indicates that protein phosphatases may act as specificity determinants in MAPK signaling (Umezawa et al., 2009). The next candidate is the splicing factor U2af responsible for removing introns from precursor mRNAs (pre-mRNAs) in all eukaryotes. Park et al. (2019) showed that normal plant development, including floral transition, and male gametophyte development in *Arabidopsis* require two U2af isoforms. However, the specific molecular mechanisms related to the regulation of splicing for the control of plant growth, reproduction, and stress response are still unknown. The next significant SNP corresponded to “40S ribosomal protein S17-like (RP)” and was the only candidate validated in the different environments (*Spain.2019*, *Spain.Global*, and *Global*). RPs are indispensable in ribosome biogenesis and protein synthesis and play a crucial role in diverse developmental processes (Ban et al., 2000; Klein et al., 2004). In rice, RPs have been demonstrated to be involved in inflorescence development and grain filling (Saha et al., 2017), while in foxtail millet, ribosomal proteins were identified to be significantly increased during drought (Pan et al., 2018). Finally, the analysis combining all the environments also identified “rhamnogalacturonate lyase-like gene (RGL).” RGLs have been shown to have a key role during pollen tube growth and defense against pathogens in tomato (Ochoa-Jiménez et al., 2018), cell expansion and growth and plant development in cotton (Naran et al., 2007), and involvement in fruit softening and ripening (Vicente et al., 2007).

Pods per plant (PP) was the trait showing a higher number of MTAs stable among environments and/or pleiotropic with the highly correlated SPL trait. Stable candidates were “protection of telomeres protein 1b (POT1),” “carboxyl-terminal peptidase (CTP),” “cytochrome c oxidase assembly protein COX15,” “pyrophosphate-energized vacuolar membrane proton pump,” and “E3 ubiquitin-protein ligase PRT1.” Telomeres are nucleoprotein complexes that physically cap the ends of chromosomes preventing them from rapid degradation. “Carboxyl-terminal peptidase (CTP)” represents an unusual and poorly understood class of serine proteases found in a broad range of organisms, controlling multiple cellular processes (binding, posttranslational modifications, and trafficking), through posttranslational modification of proteins (Carroll et al., 2014). COX is the last enzyme of the mitochondrial respiratory chain, playing a key role in the regulation of aerobic production of energy. The lack of mutant plants in COX components, due to embryonic lethality, highlights the importance of COX activity in plants (Mansilla et al., 2018). Next, vacuolar proton pyrophosphatases facilitate auxin biosynthesis, transport, signaling, conjugation, and catabolism during seed development (Cao et al., 2020). Auxin is a regulator of yield contributing to ovule and seed growth, morphogenesis, and progression through different reproductive stages in *Arabidopsis*, rice, or maize among other examples (Shirley et al., 2019). Finally, E3 ubiquitin-ligases are involved in the regulation of plant innate immunity (Duplan and Rivas, 2014) and also affect the induction of flowering in angiosperms, ensuring their formation when conditions are optimal for pollination and that seeds or subsequent seedlings have time to develop. Pleiotropic candidates were “cleavage stimulation factor subunit 50 (CstF-50),” “protein JINGUBANG (JGB),” “DNA repair protein RAD51 homolog,” and “protection of telomeres protein 1b (POT1)” already mentioned above. CstF-50 has a regulatory role that is indispensable for the biogenesis of mRNA and participates in the control of gene expression under DNA-damaging conditions by regulating polyadenylation/deadenylation (Cevher et al., 2010). JGB is a pollen-specific protein containing seven WD40 repeats that regulate pollen germination and tube growth ensuring pollination in moist environments (Ju et al., 2016). Further analysis of the group revealed that JGB interacts with the transcription factor TCP4 to control pollen jasmonic acid synthesis. Interestingly, the consistent and pleiotropic effect of JGB with three yield components (PP, SP, and HSW) may be crucial for molecular breeding of yield-related traits in faba bean. Finally, RAD51 proteins contribute to genome stability by repairing DNA damage after replication, transcription, or cellular metabolic activities and play a direct role in the control of immune responses (Wang et al., 2010). Moreover, the phenotypic characterization of RAD51 in *Arabidopsis* revealed that this gene has an essential function in male and female meiosis (Li et al., 2004).

Seeds per plant (SPL) was the trait revealing more unknown or uncharacterized proteins. Five candidates were pleiotropic with other yield-related traits: LYK5 (with SPL and SH) and CstF-5, JGB, POT1, and RAD51 (with PP and SPL) explained above.

For hundred-seed weight (HSW), apart from JINGUBANG (JGB), the four SNPs with corresponding candidate genes, explaining a relevant percentage of the variation, were “protein STICHEL-like 2,” whose role in seed size modulation is still not known; “ubiquitin carboxyl-terminal hydrolase 1”; “hydroxyproline O-galactosyltransferase HPGT1”; and “BRISC and BRCA1-A complex member 2.” Several components of the ubiquitin pathway have been found to play critical roles in the regulation of seed and organ size in *Arabidopsis* and rice (Li and Li, 2014) and in cacao (Bekele et al., 2022). “Hydroxyproline O-galactosyltransferase HPGT1” has a functional role in various aspects of plant growth, development, and fertility (e.g., germination, seed set, seed size and morphology, and silique length) in *Arabidopsis* (Kaur et al., 2021). Finally, “BRISC and BRCA1-A complex member 2” has been shown to be involved in DNA repair (Block-Schmidt et al., 2011). Other candidates explaining a lower percentage but putatively involved in seed development were “mannan endo-1,4-beta-mannosidase 6”, “UPF0307 protein PMI3641,” and “oleoyl-acyl carrier protein thioesterase 1.”

Finally, in the case of plot yield (PY), nine of the candidates harboring the significant SNPs explained more than 10% of the variation. “DNA-binding protein RHL1” (ROOT HAIRLESS 1 gene) encodes a nuclear protein required for root hair initiation in *Arabidopsis* (Schneider et al., 1998) and for ploidy-dependent cell growth (Sugimoto-Shirasu et al., 2005). The next candidate was “hypersensitive-induced response protein 1” (HIR1). A highly conserved interaction between receptor-like protein kinase (LRR1) and “hypersensitive-induced response protein 1” (HIR1) homologs is a common mechanism in the defense response of both monocots and dicots (Zhou et al., 2009). “Sucrose-phosphate synthase (SPS)” is a key enzyme in the plant sugar metabolic pathways with functions on growth, development, and yield. Several authors have reported that enhancement of SPS activity provides a higher carbon partitioning that increases growth and grain yield in rice (Sharkey et al., 2000; Mulyatama et al., 2022) or potato (Ishimaru et al., 2008). The next candidate is “basic endochitinase” reported to enhance the defense system of plants (as they act on chitin, the major component of the cell wall) and also to improve plant growth and yield in different crops (Gongora and Broadway, 2002; Jeong et al., 2010). Next, “LRR receptor-like serine/threonine-protein kinases RCH1” has been reported to play vital roles in plant growth and development and the responses to environmental stress (Liu et al., 2017), while “E3 ubiquitin-protein ligase BRE1-like 1” and the ubiquitin system in general affect plant health, reproduction, and responses to the environment, processes that impact important agronomic traits such as the induction of flowering, yield, and pathogen responses (Linden and Callis, 2020).

The relatively large amounts of MTAs associated with yield components identified in this study are promising candidates for follow-up studies on the validation of genes controlling faba bean production. Although most of the MTAs were locally relevant in response to the conditions of a particular location or year, 12 of them (**Supplementary Table S4**) were stable across different environments and/or were associated with multiple traits. The MTAs identified or the closely linked genes on either flanking side of the significant SNPs are likely to represent significant candidates for the molecular breeding of faba bean yield-related traits.

## Conclusion

Genetic dissection of the genomic regions controlling faba bean yield is of great interest for the development of highly productive varieties. By phenotyping a worldwide collection of 400 faba bean accessions in different environments and using a GWAS analysis, this study provides a comprehensive genomic resource for the genetic dissection of yield components in this crop. A wide variability, a high heritability, and a high positive correlation were observed for most of the traits (except for the final PY) in all the environments studied. Overall, the panel revealed 112 associated MTAs linked with six traits. Several clusters of associated markers were distributed along the genome and highlighted important genomic associations. The identification of consistent MTAs that are stable across different environments is of great value to MAS in breeding genotypes adapted to diverse ecological environments. Five of these candidates were also pleiotropic and co-localized with different highly phenotypic correlated traits (i.e., SH, SP, and SPL). Pleiotropic effects are also beneficial in the breeding process as they allow breeders to simultaneously select for multiple traits. Gene annotation showed that the highest percentages of candidates identified have unknown functions or were not found in the current version of the faba bean genome. The detection of new gene sequences in very large and highly repetitive genomes such as that of faba bean remains a significant challenge due to the presence of significant gaps, highlighting the need for a high-quality genome assembly. Even though these candidates or the closely linked genes flanking the significant SNPs remain to be validated in different genetic backgrounds, the identified markers provide a valuable genetic resource for future marker-assisted selection and fine mapping of the genes underlying yield improvement in this crop.

## Data availability statement

The original contributions presented in the study are included in the article/**Supplementary Materials**, further inquiries can be directed to the corresponding author/s.

## Author contributions

NG: Conceptualization, Formal Analysis, Resources, Writing – original draft, Methodology. MP: Formal Analysis, Software, Supervision, Writing – review & editing. IS: Data curation, Visualization, Writing – review & editing. DS: Data curation, Visualization, Writing – review & editing. DL: Data curation, Visualization, Writing – review & editing. CH: Data curation, Visualization, Writing – review & editing. AT: Conceptualization, Funding acquisition, Project administration, Resources, Supervision, Writing – original draft.
