# Supplementary material for: Genome-wide association study for yield-related traits in faba bean (Vicia faba L.)
Source: Front Plant Sci. 2024 Mar 13;15:1328690. doi: 10.3389/fpls.2024.1328690 (PMC10965552; doi:10.3389/fpls.2024.1328690)

Supplementary Figure S1. Density distribution of the five yield related traits measured in 352 faba bean accessions across different environments.

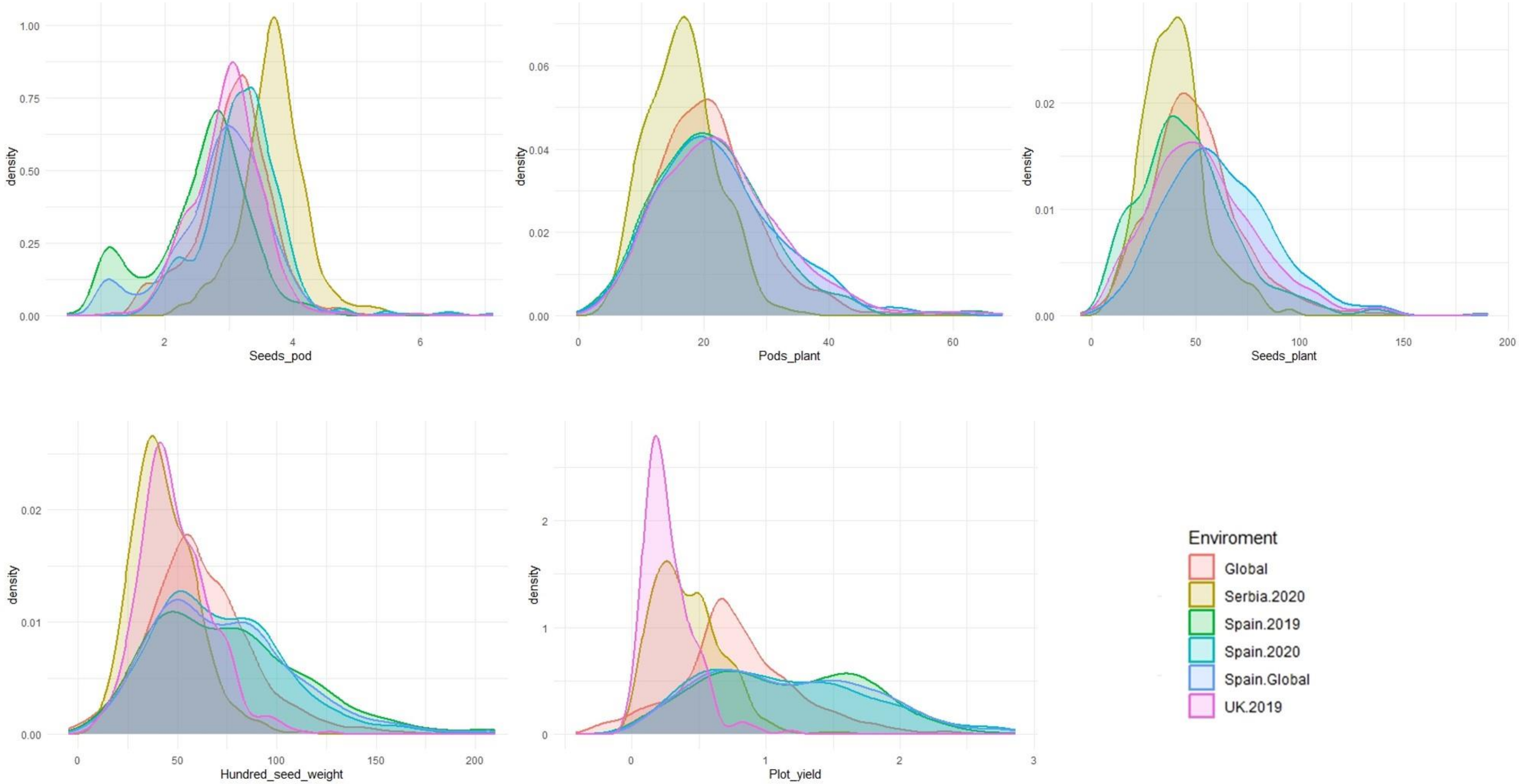

Supplement: Supplementary file 8 [file Image_1.pdf]
