# Supplementary material for: Genome-wide association study for yield-related traits in faba bean (Vicia faba L.)
Source: Front Plant Sci. 2024 Mar 13;15:1328690. doi: 10.3389/fpls.2024.1328690 (PMC10965552; doi:10.3389/fpls.2024.1328690)
Supplement: Supplementary file 9 [file Image_2.pdf]

Supplementary Figure S2. Manhattan plots (left) and quantile-quantile (QQ) plots (right) for six yield related traits across different environments.The manhattan plots show the p-values association between a phenotypical trait and each tested marker (expressed as the negative decimal logarithm of the p, y-axis) plotted against their respective positions on each chromosome (X-axis). Significant markers above the cutoff value (Bonferroni threshold -log10 (p) > 5.66) were associated with the respective trait. The QQ plots show the observed p-values for association between a phenotypical trait and each marker, expressed as –log10 of p (Y-axis) plotted against –log10 of the expected p-values (x-axis) under the null hypothesis of no association for the analysis. The plots are named by the corresponding trait followed byf the environment. SP: seeds per pod; PP: pods per plant; SPL: seeds per plant; HSW: hundred seed weight; PY: plot yield.

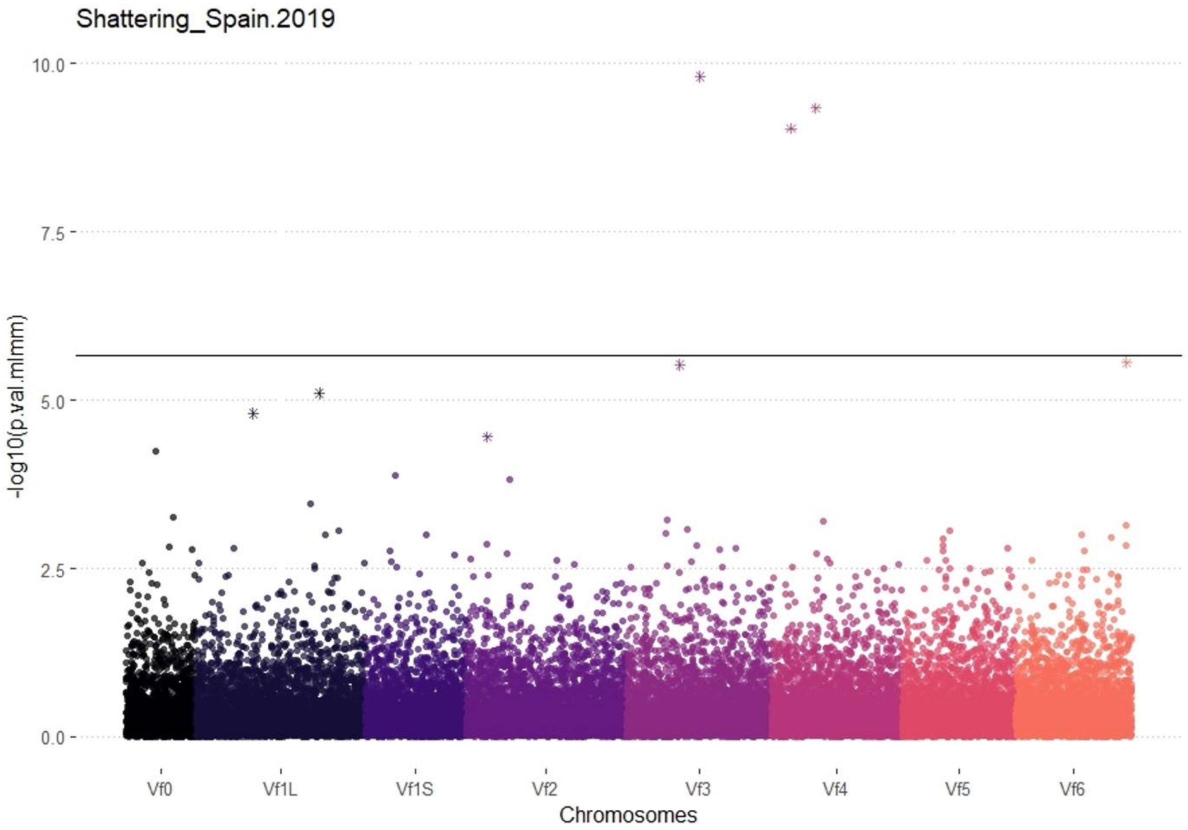

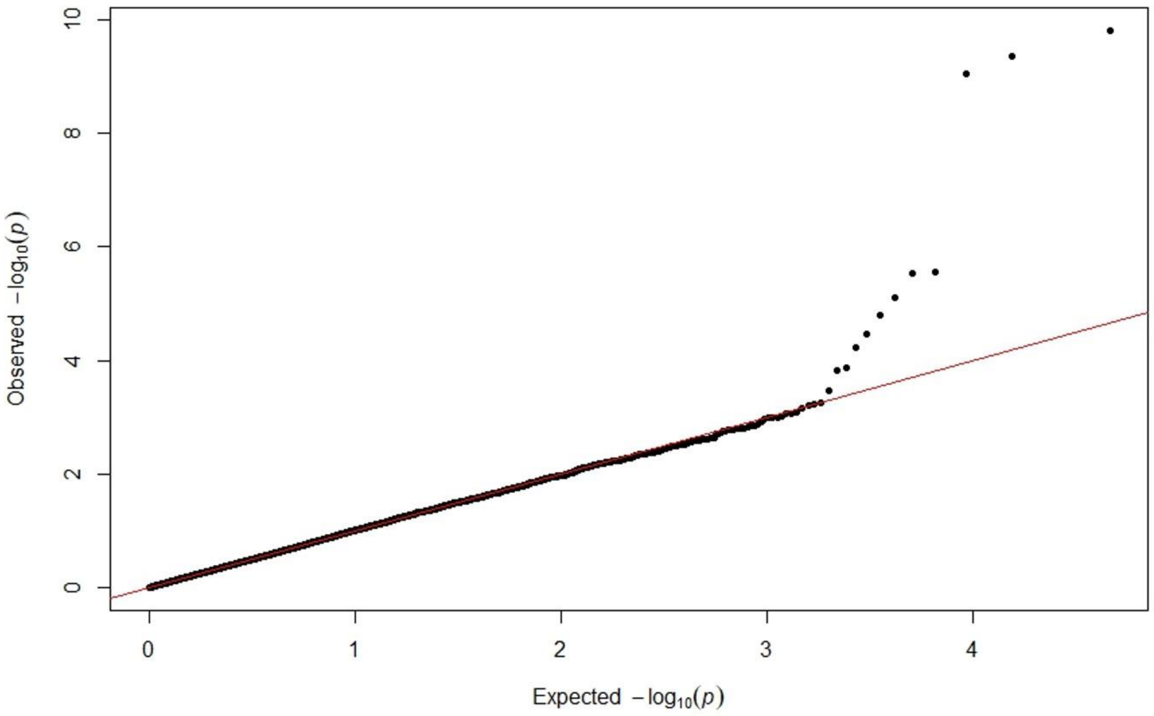

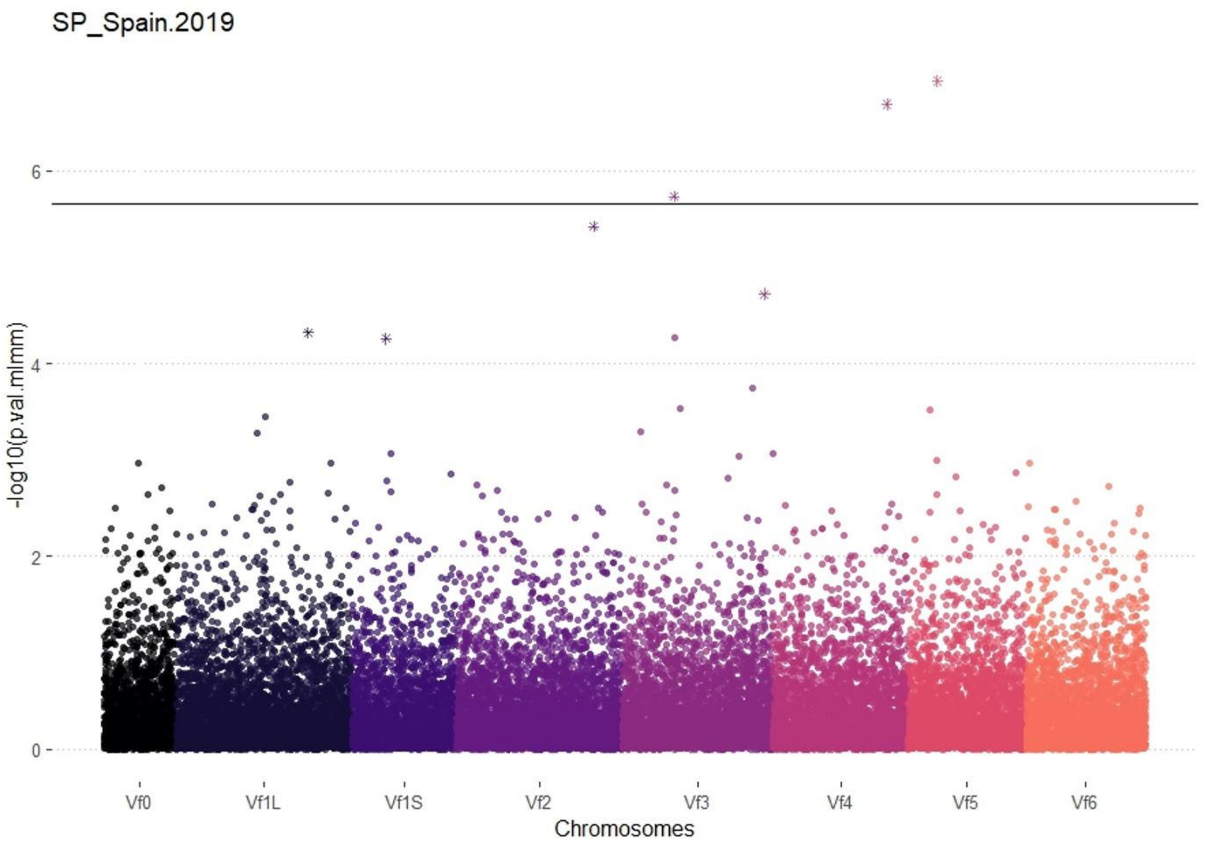
SP_Spain.2019
-log10(p.val.mlmm)
0
2
4
6
Vf0
Vf1L
Vf1S
Vf2
Vf3
Vf4
Vf5
Vf6
Chromosomes
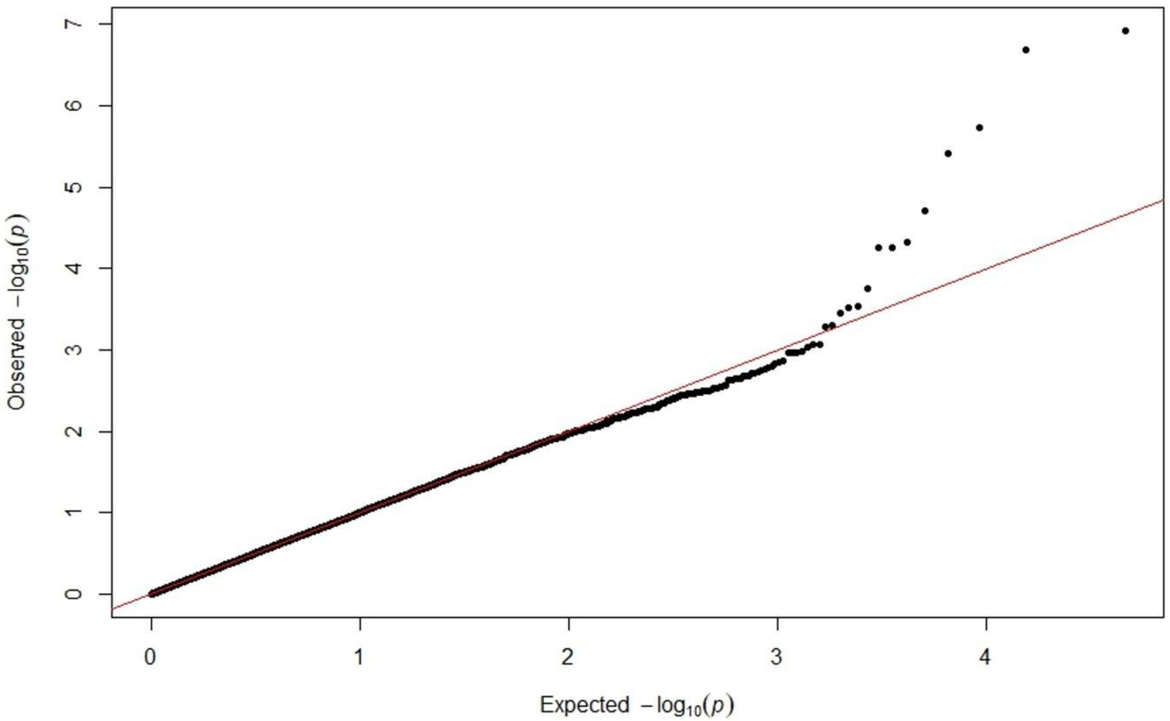
Observed $-\log_{10}(p)$
Expected $-\log_{10}(p)$
0
1
2
3
4
5
6
7

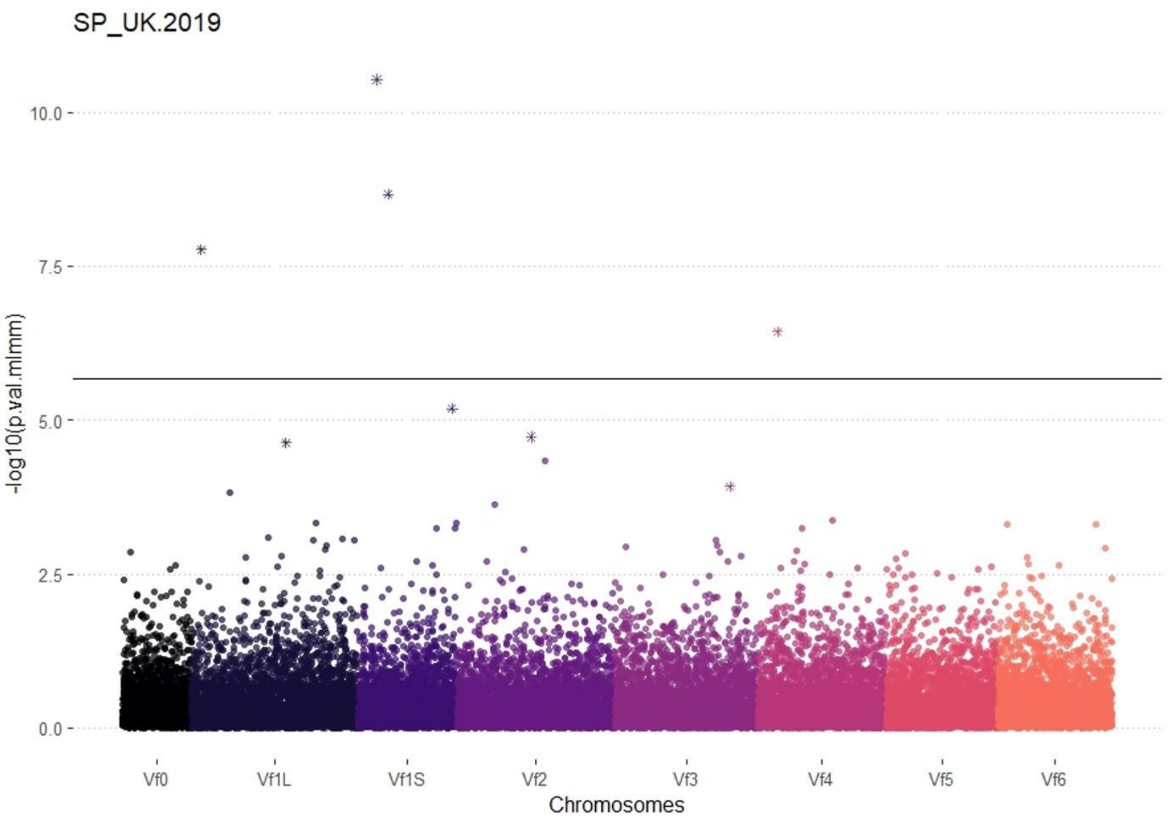
SP_UK.2019
-log10(p.val.mlmm)
10.0
7.5
5.0
2.5
0.0
Vf0
Vf1L
Vf1S
Vf2
Vf3
Vf4
Vf5
Vf6
Chromosomes

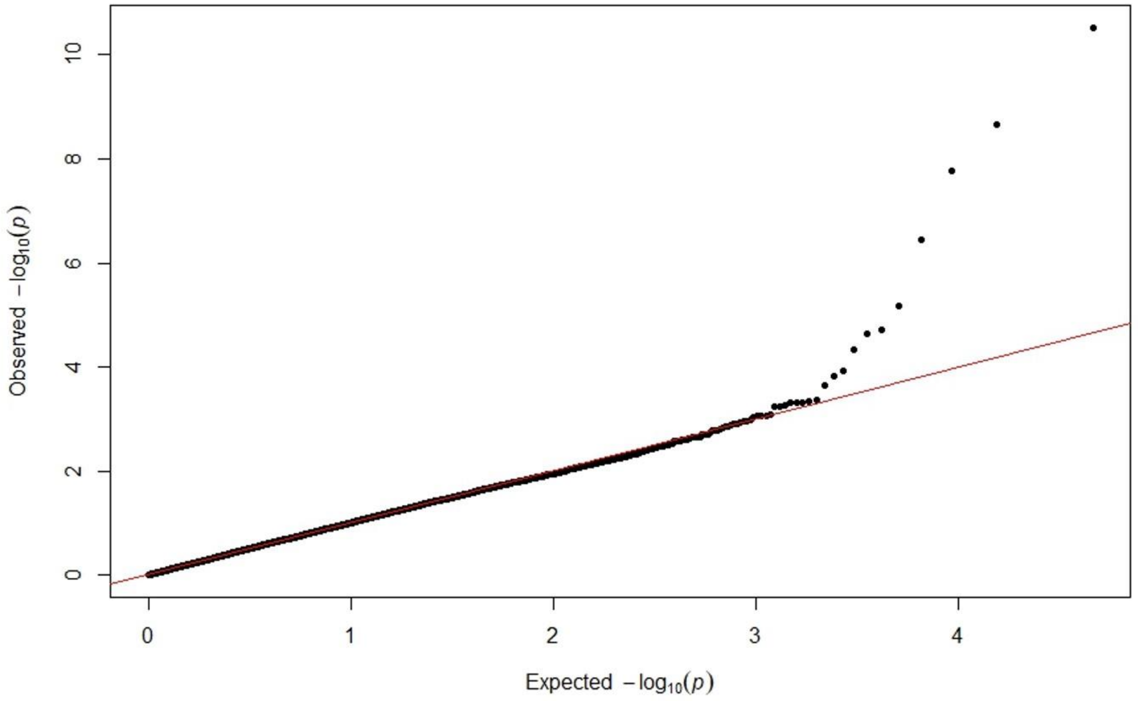
Observed −log10(p)
Expected −log10(p)
0
2
4
6
8
10
0
1
2
3
4

SP_Spain.Global

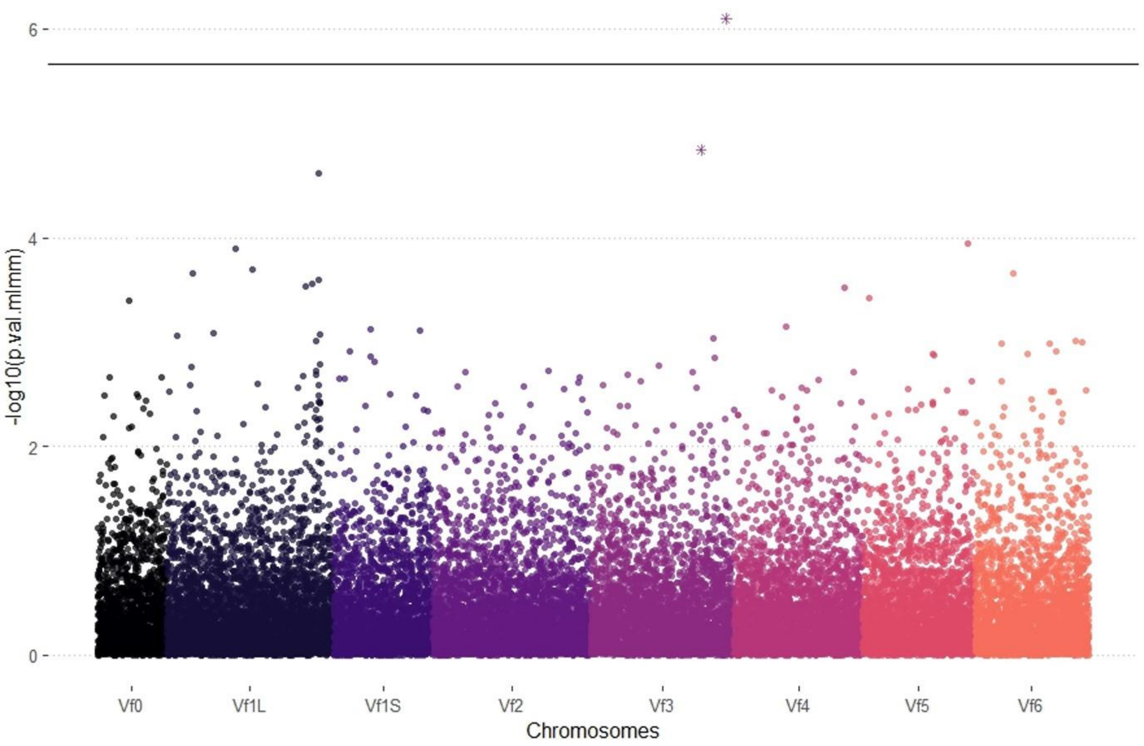

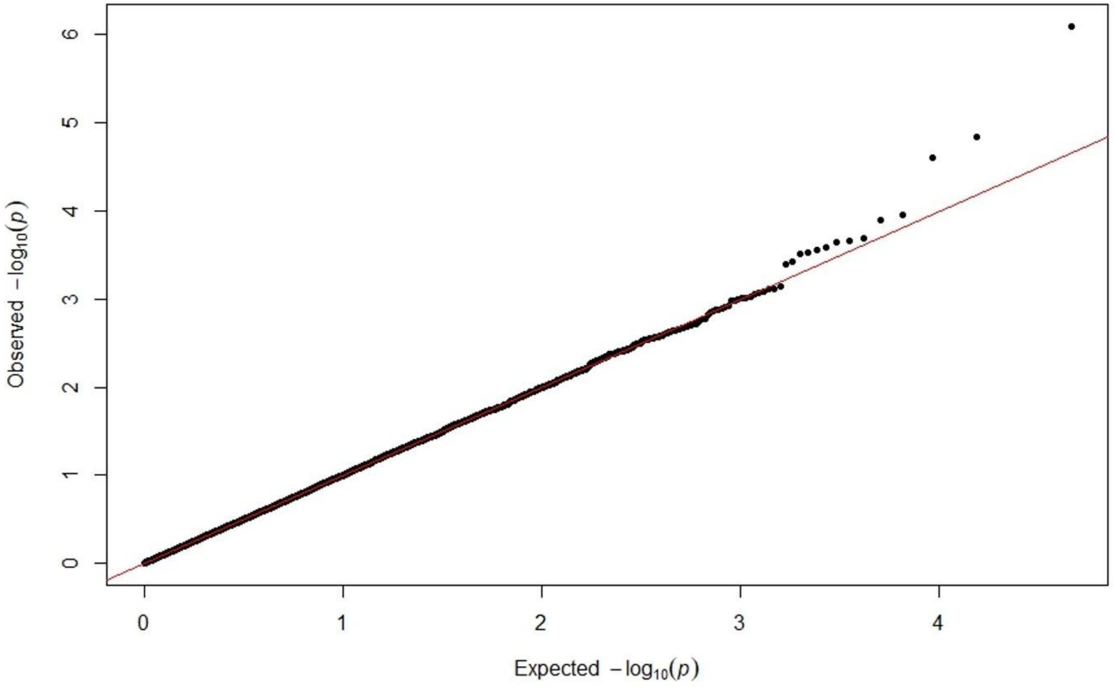

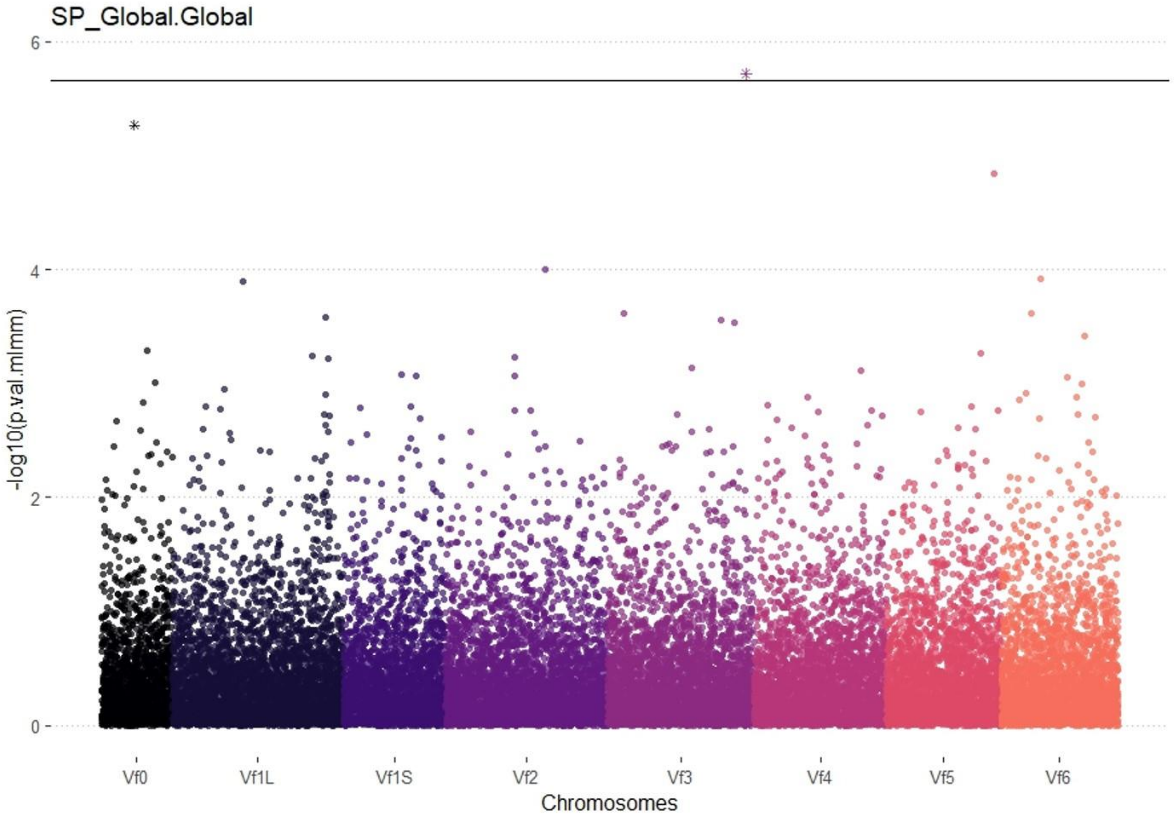

SP_Global.Global
-log10(p.val.mlmm)
6
4
2
0
Vf0
Vf1L
Vf1S
Vf2
Vf3
Vf4
Vf5
Vf6
Chromosomes

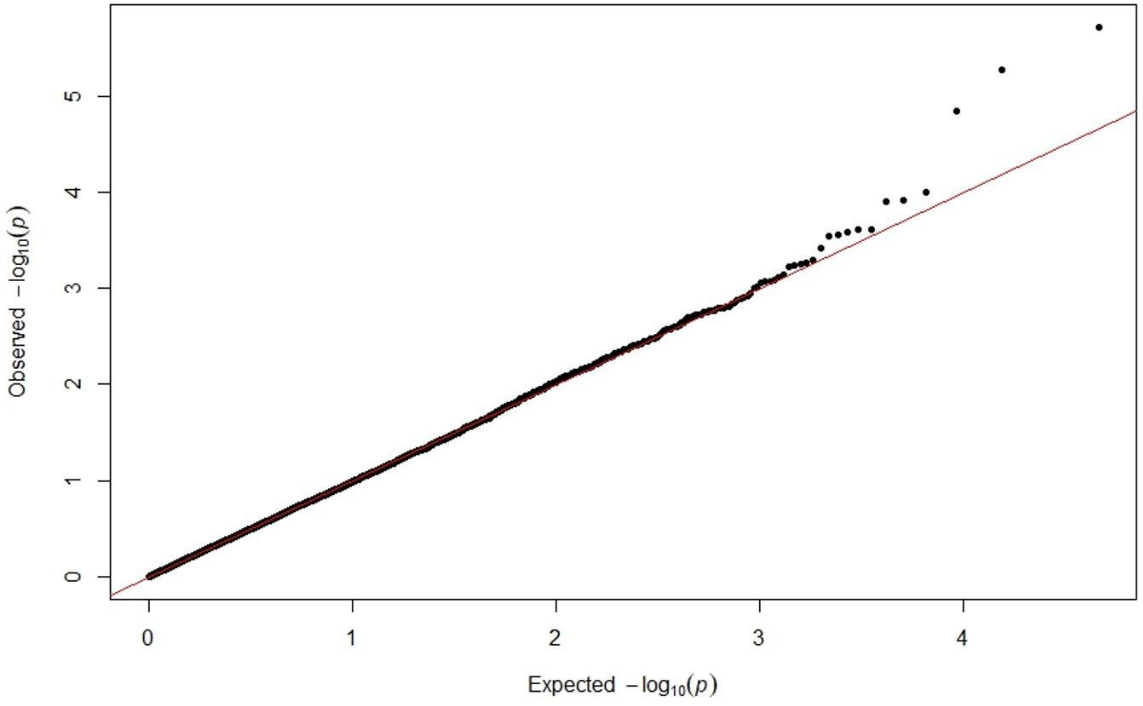

Observed $-\log_{10}(p)$
Expected $-\log_{10}(p)$
0
1
2
3
4
5

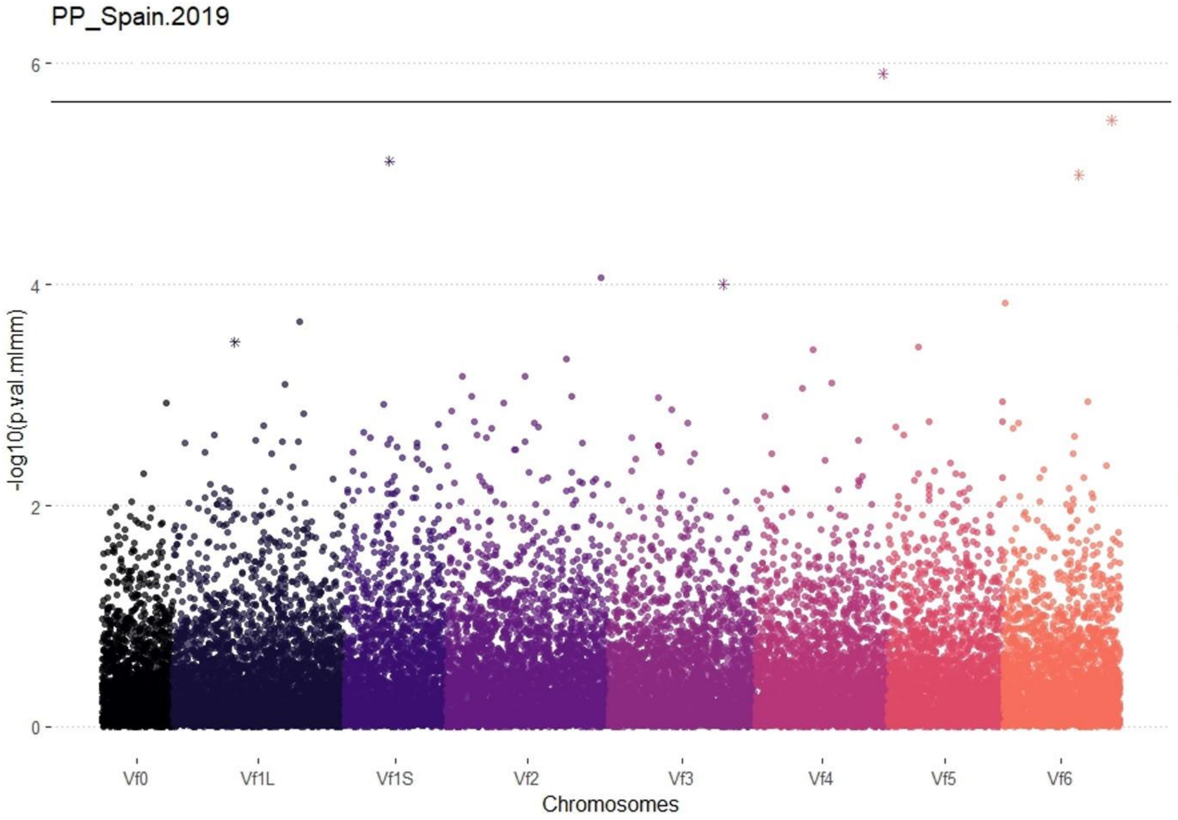
PP_Spain.2019
-log10(p.val.mlmm)
Chromosomes
Vf0
Vf1L
Vf1S
Vf2
Vf3
Vf4
Vf5
Vf6

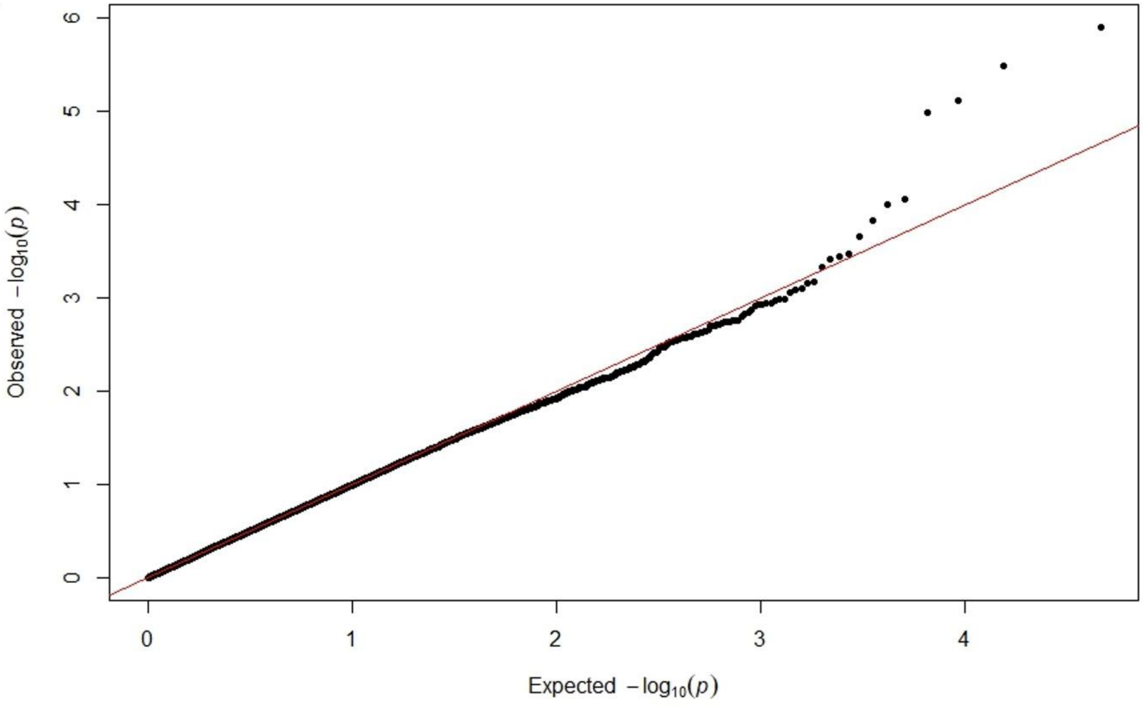
Observed $-\log_{10}(p)$
Expected $-\log_{10}(p)$

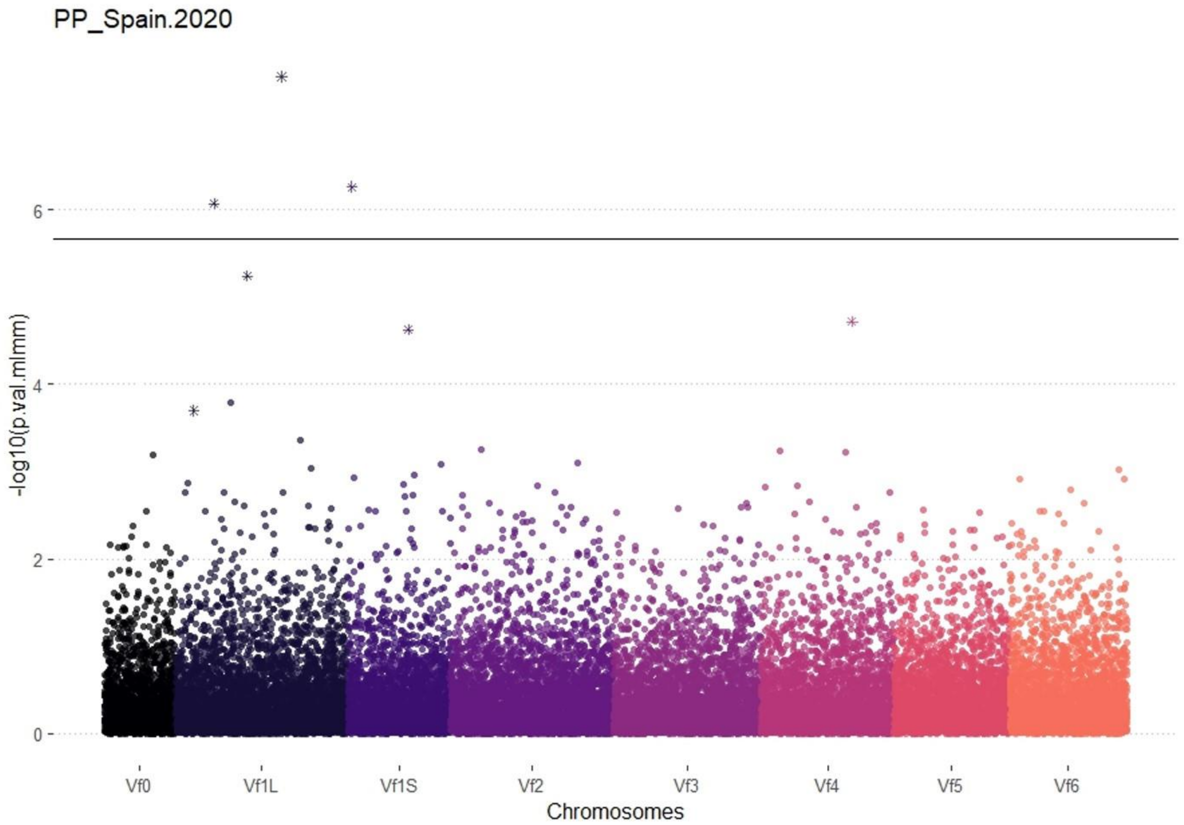
PP_Spain.2020
-log10(p.val.mlmm)
0
2
4
6
Vf0
Vf1L
Vf1S
Vf2
Vf3
Vf4
Vf5
Vf6
Chromosomes

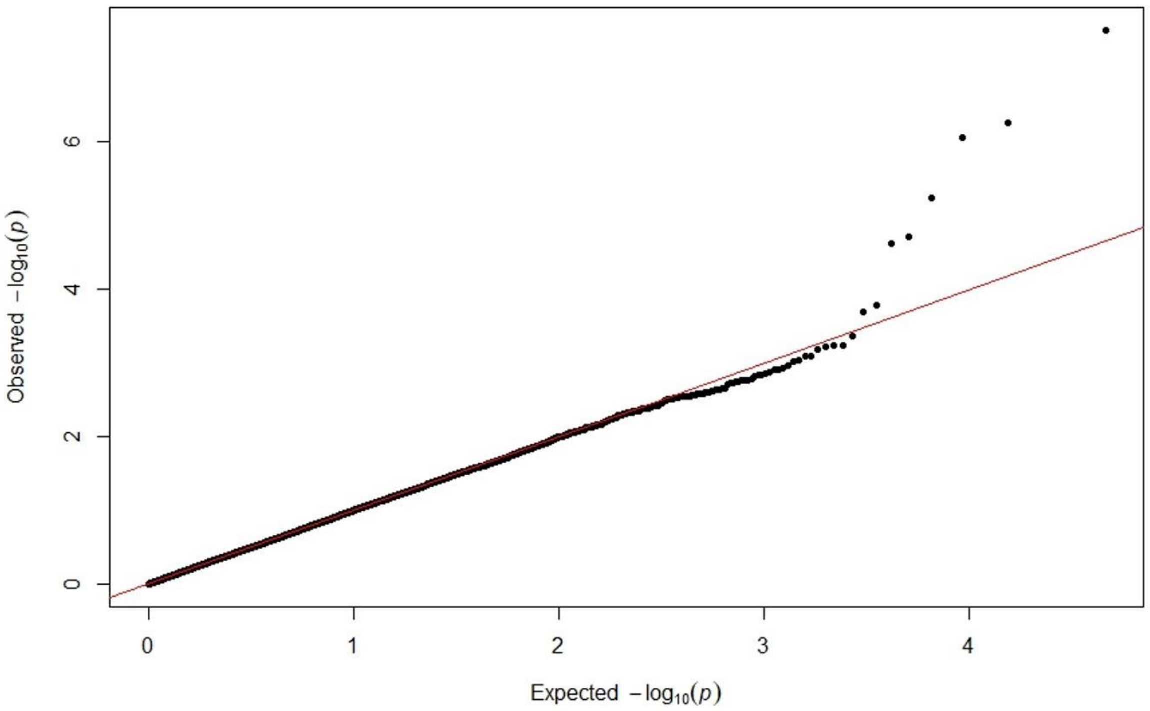
Observed $-\log_{10}(p)$
0
2
4
6
0
1
2
3
4
Expected $-\log_{10}(p)$

PP_Serbia.2020

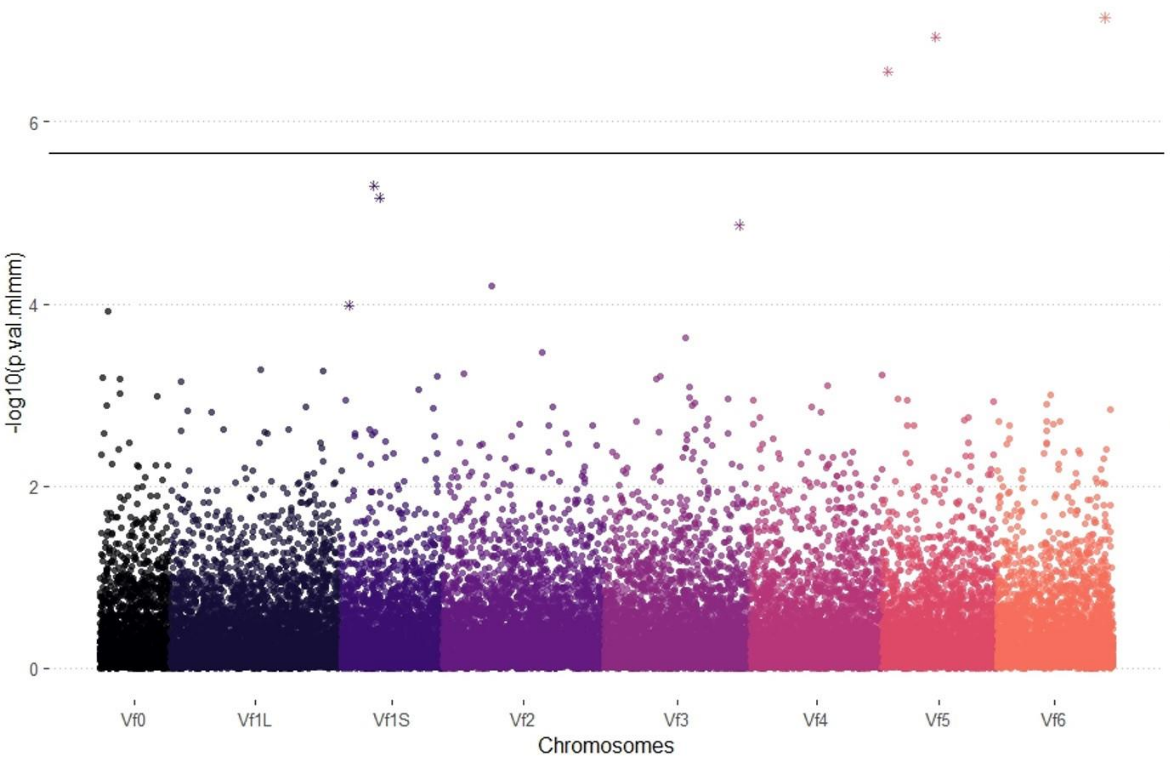

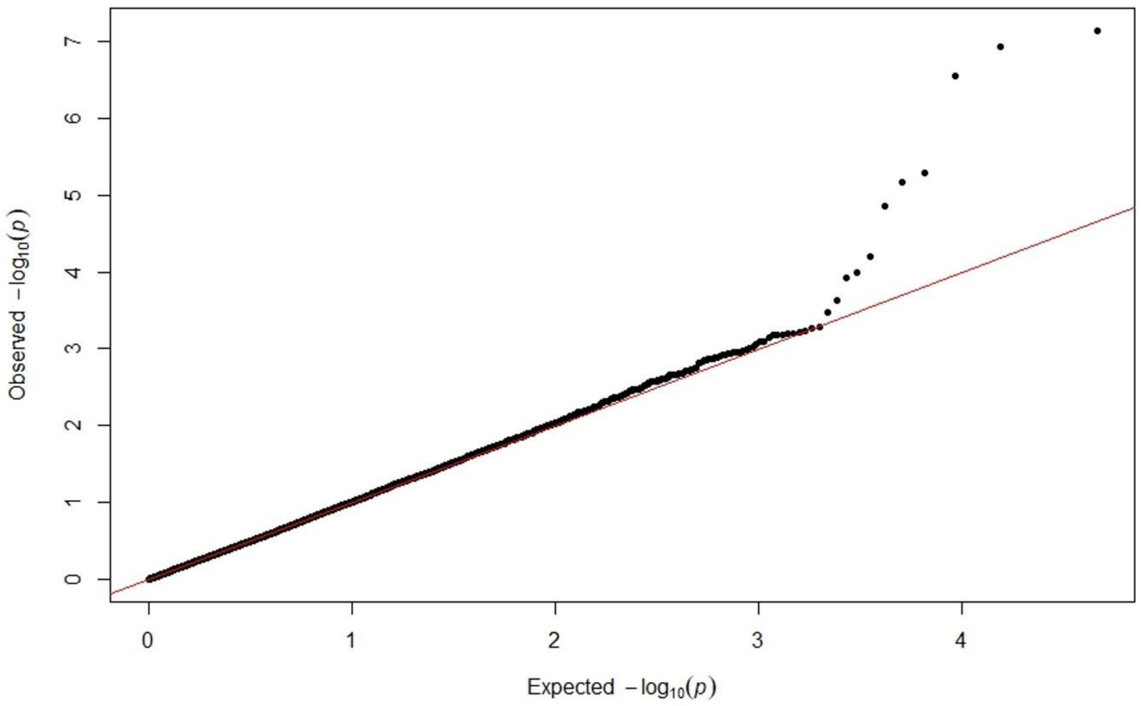

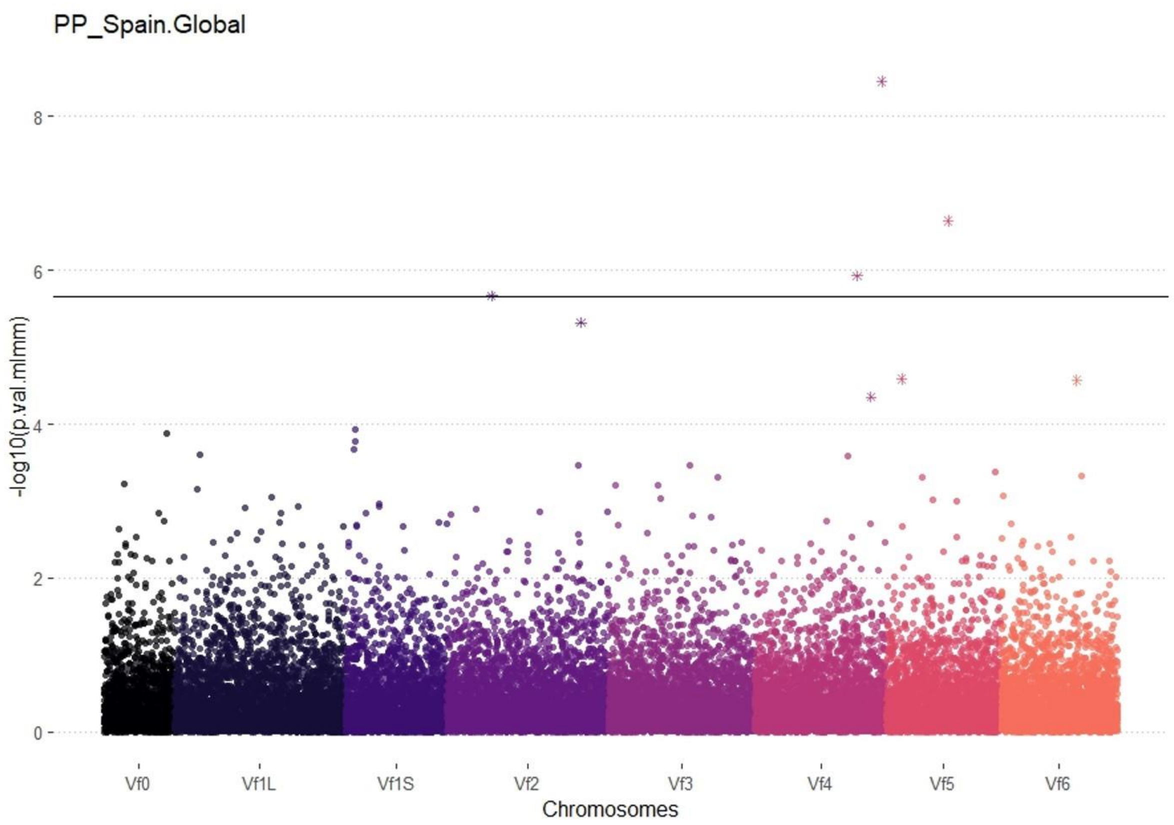

PP_Spain.Global
-log10(p.val.mlmm)
0
2
4
6
8
Vf0
Vf1L
Vf1S
Vf2
Vf3
Vf4
Vf5
Vf6
Chromosomes

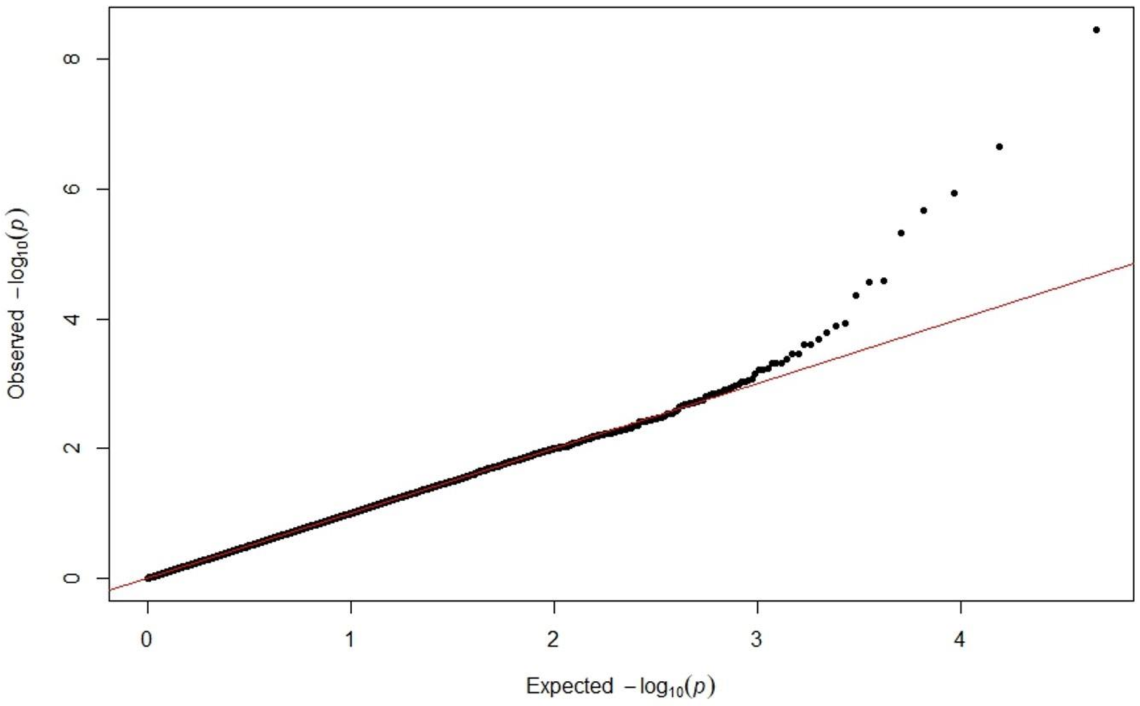

Observed $-\log_{10}(p)$
Expected $-\log_{10}(p)$
0
1
2
3
4
0
2
4
6
8

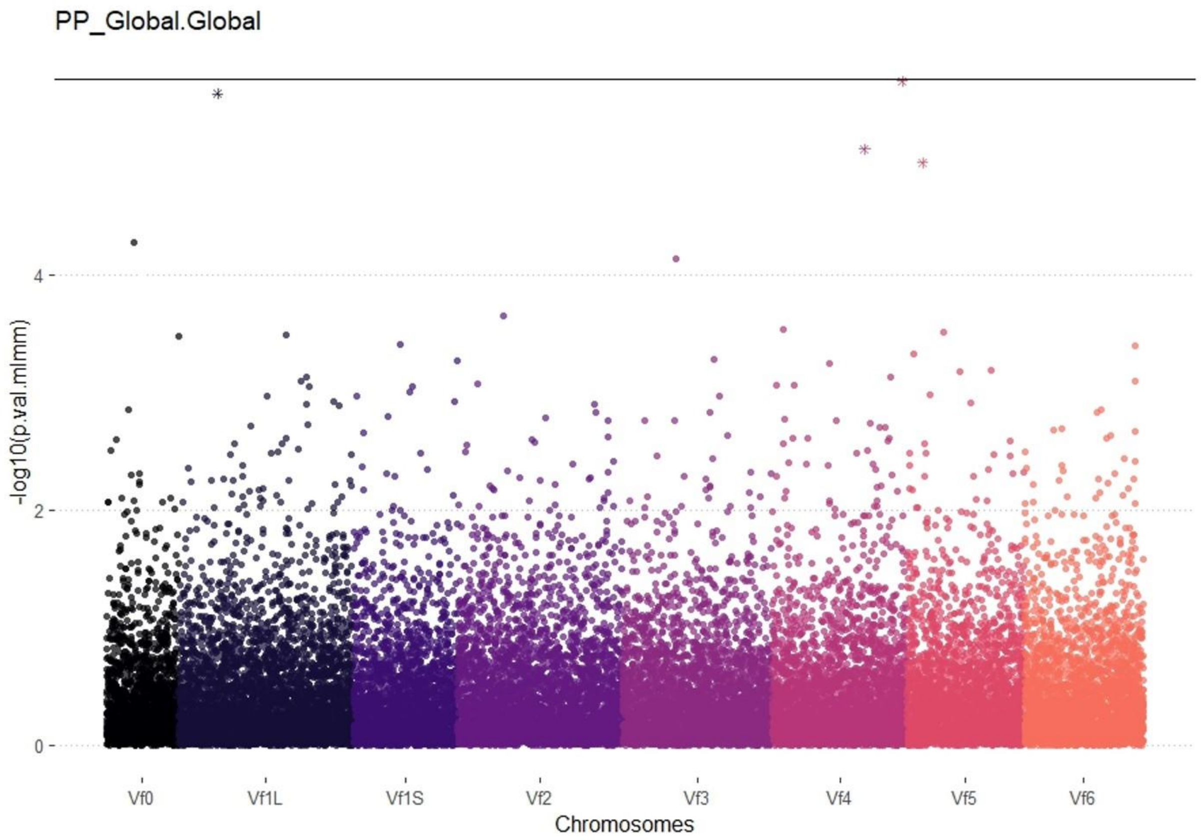
PP_Global.Global
-log10(p.val.mlmm)
0
2
4
Vf0
Vf1L
Vf1S
Vf2
Vf3
Vf4
Vf5
Vf6
Chromosomes

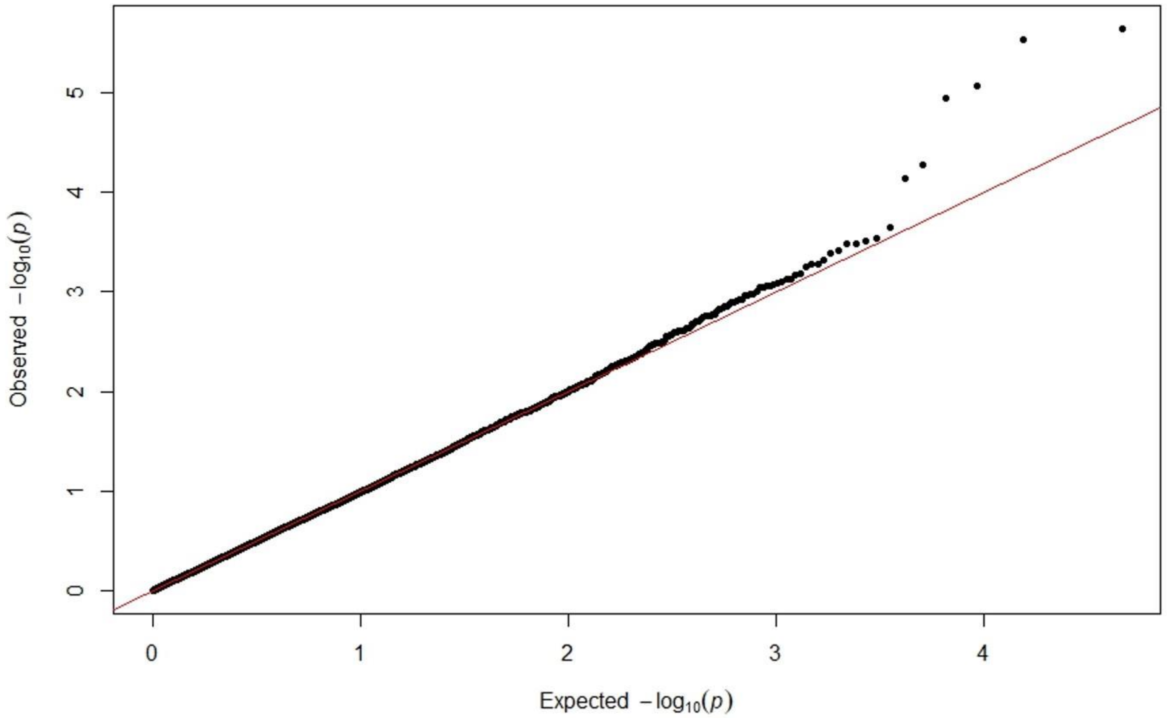
Observed $-\log_{10}(p)$
0
1
2
3
4
5
Expected $-\log_{10}(p)$

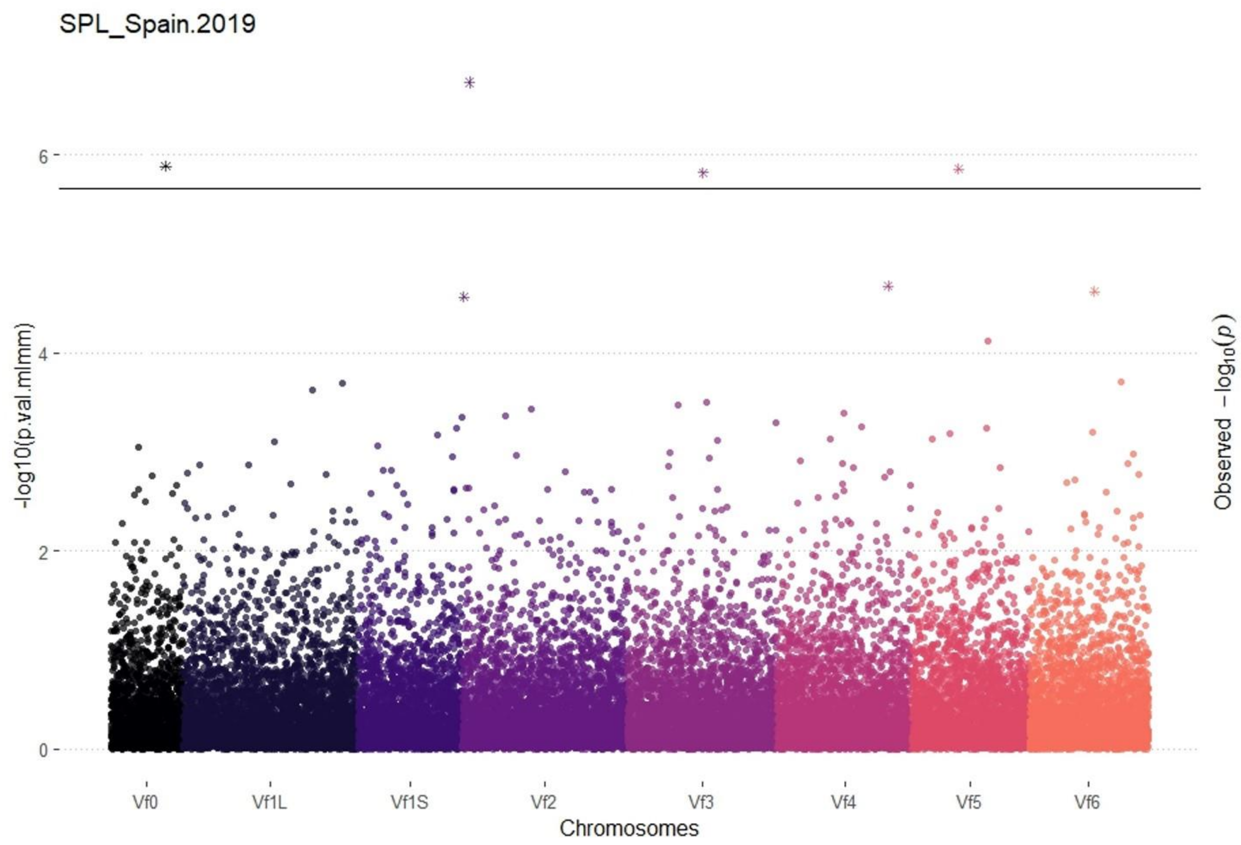

SPL_Spain.2019
-log10(p.val.mlmm)
6
4
2
0
Vf0
Vf1L
Vf1S
Vf2
Vf3
Vf4
Vf5
Vf6
Chromosomes

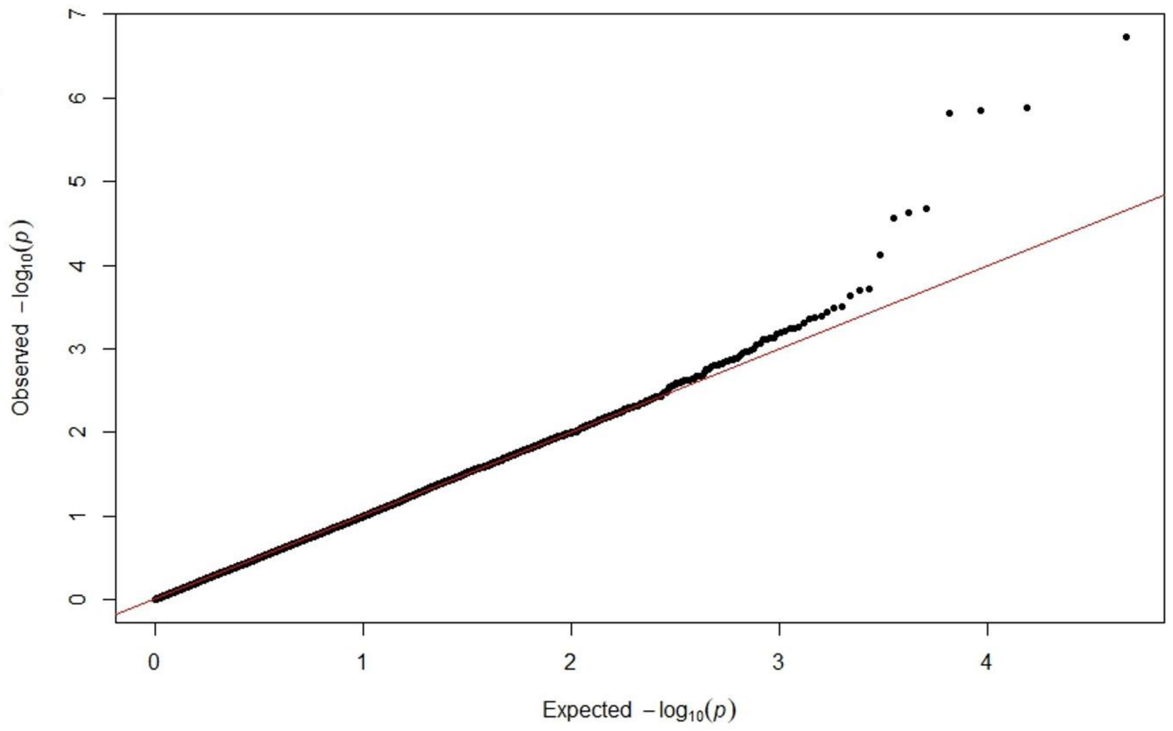

Observed $-\log_{10}(p)$
Expected $-\log_{10}(p)$
0
1
2
3
4
5
6
7

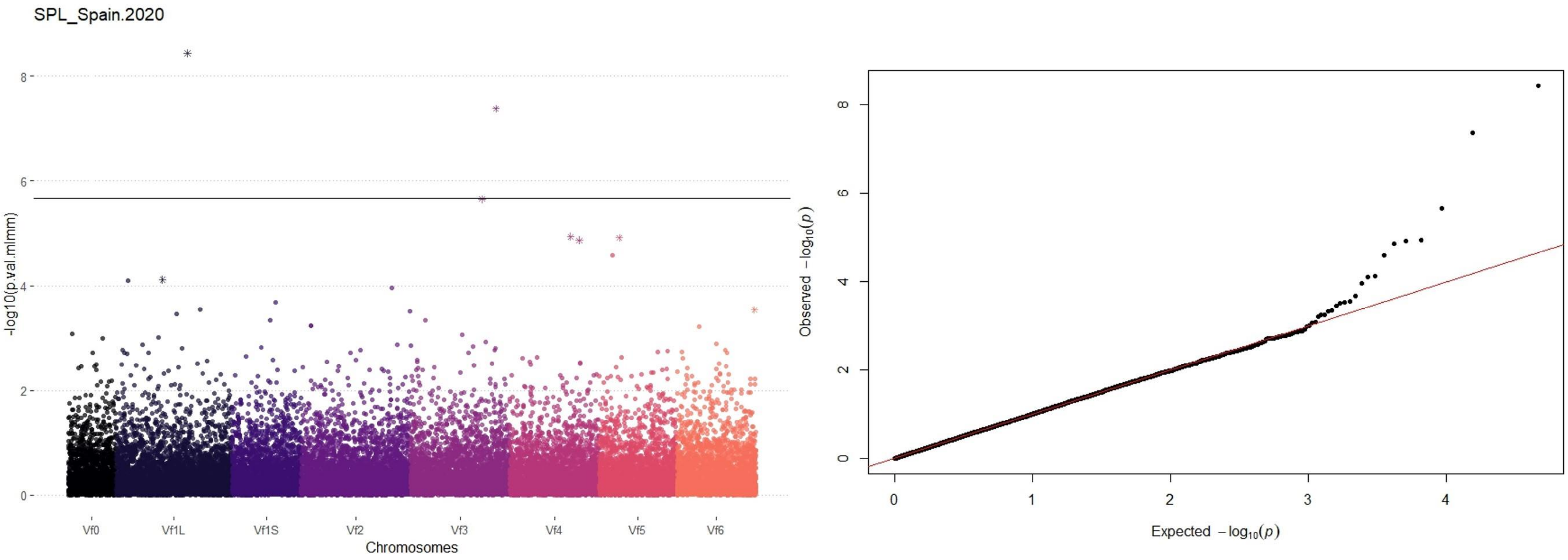

SPL_Spain.2020
-log10(p.val.mlmm)
Chromosomes
Vf0
Vf1L
Vf1S
Vf2
Vf3
Vf4
Vf5
Vf6
Observed $-\log_{10}(p)$
Expected $-\log_{10}(p)$

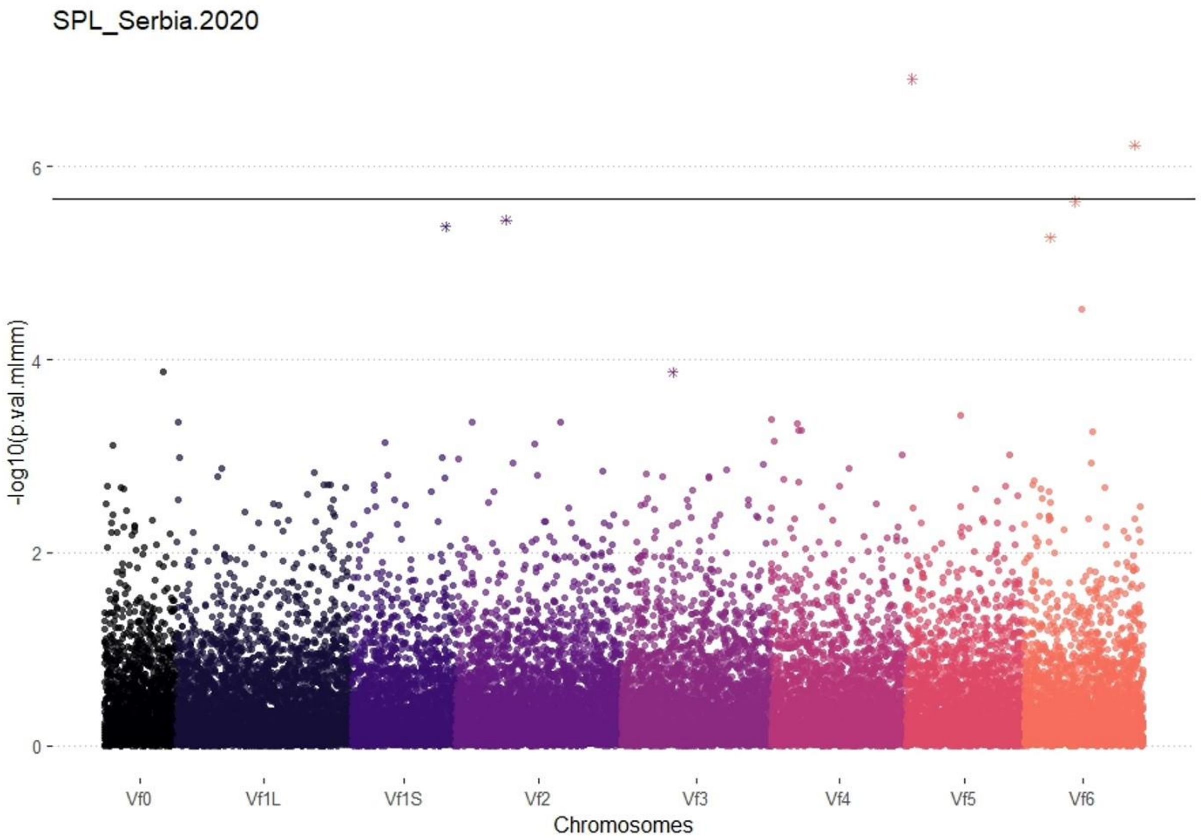
SPL_Serbia.2020
-log10(p.val.mlmm)
0
2
4
6
Vf0
Vf1L
Vf1S
Vf2
Vf3
Vf4
Vf5
Vf6
Chromosomes

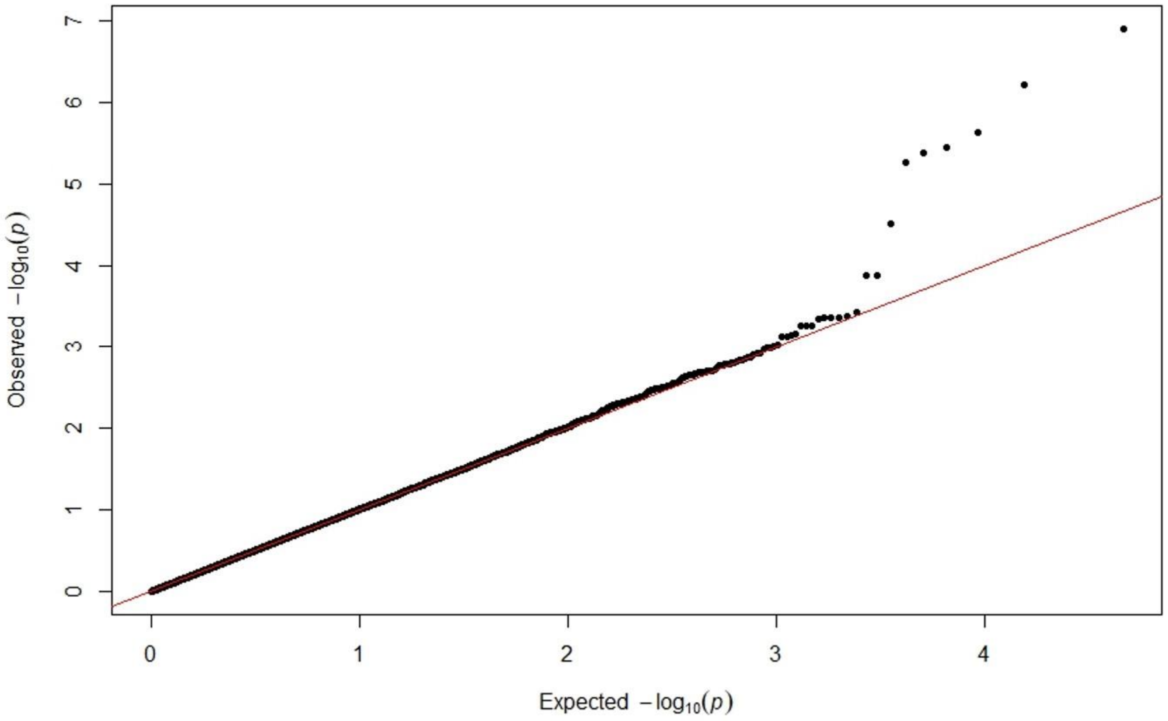
Observed $-\log_{10}(p)$
0
1
2
3
4
5
6
7
Expected $-\log_{10}(p)$

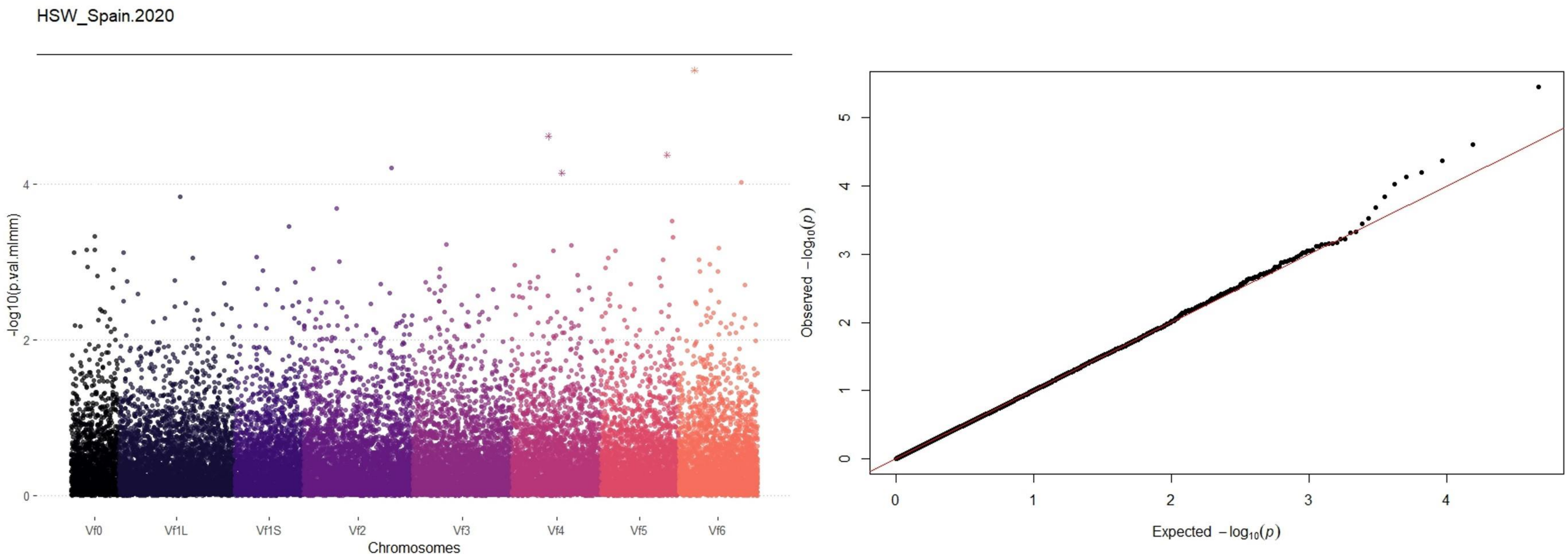
HSW_Spain.2020
-log10(p.val.mlmm)
0
2
4
Vf0
Vf1L
Vf1S
Vf2
Vf3
Vf4
Vf5
Vf6
Chromosomes
Observed −log10(p)
Expected −log10(p)
0
1
2
3
4
5

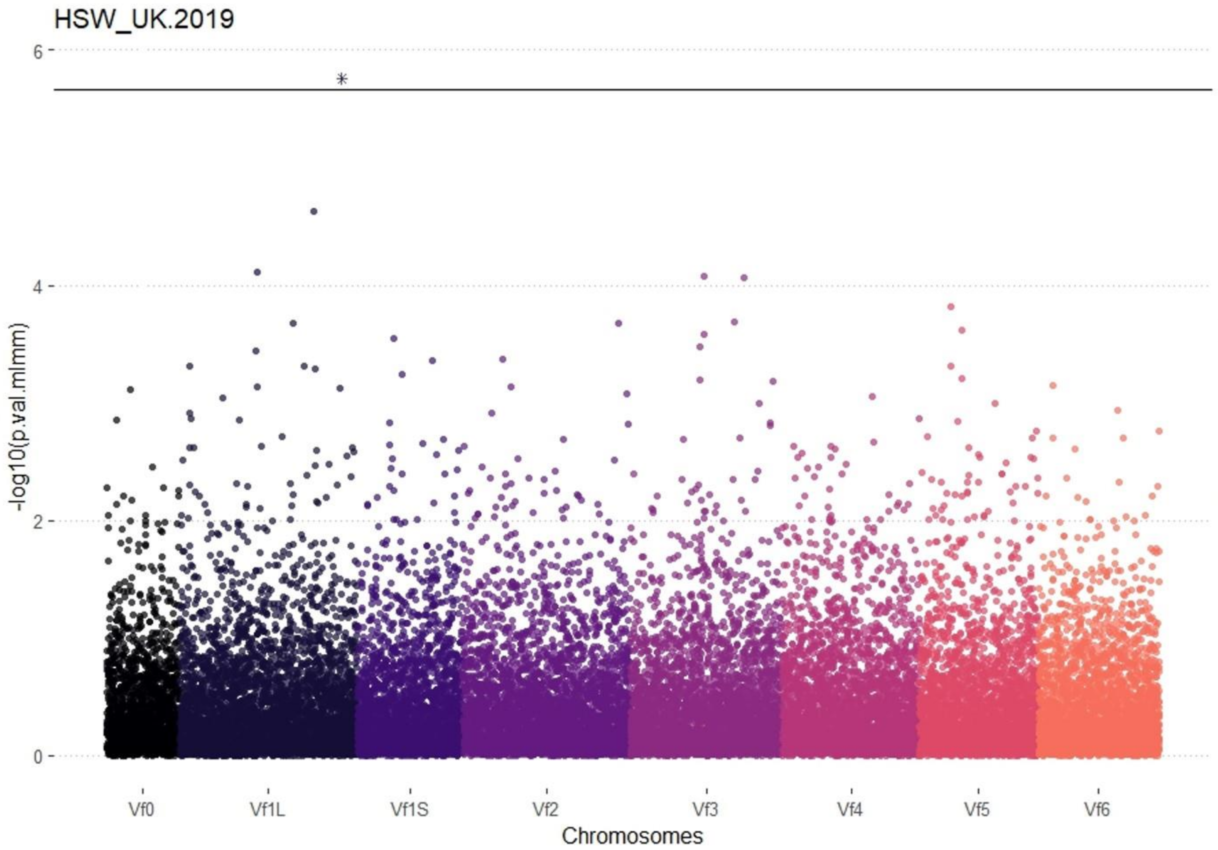

HSW_UK.2019
-log10(p.val.mlmm)
6
4
2
0
Vf0
Vf1L
Vf1S
Vf2
Vf3
Vf4
Vf5
Vf6
Chromosomes

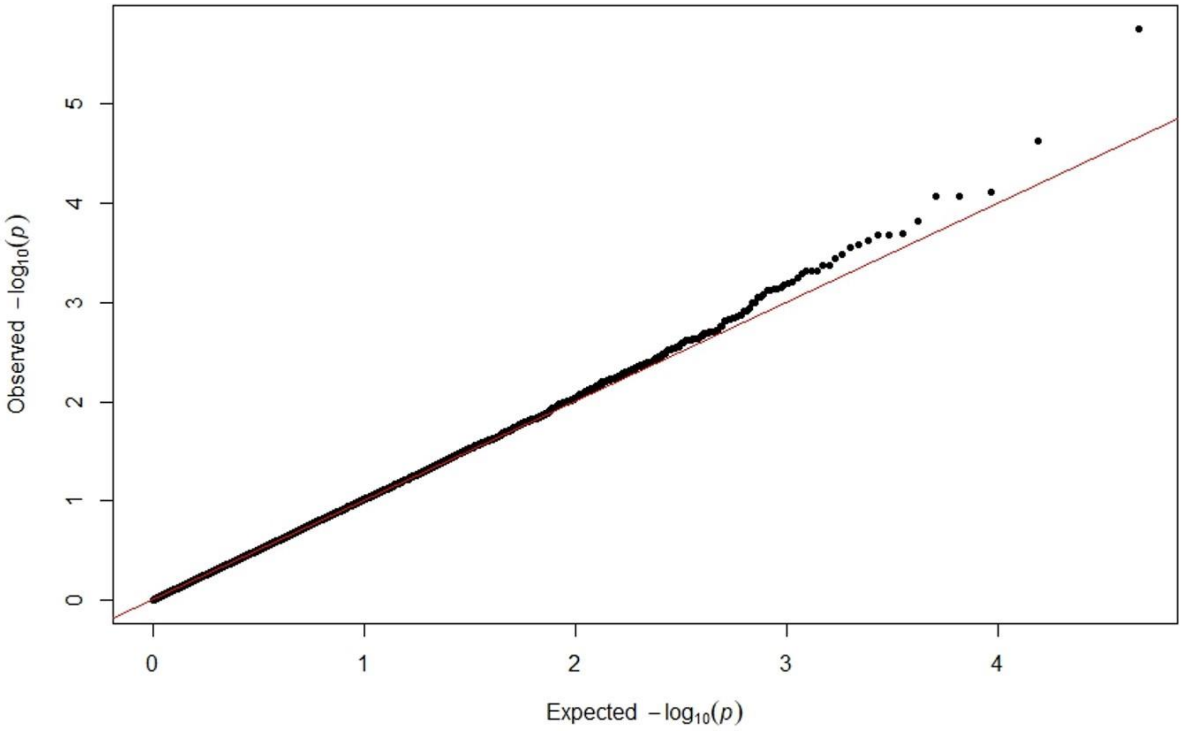

Observed $-\log_{10}(p)$
Expected $-\log_{10}(p)$
0
1
2
3
4
5

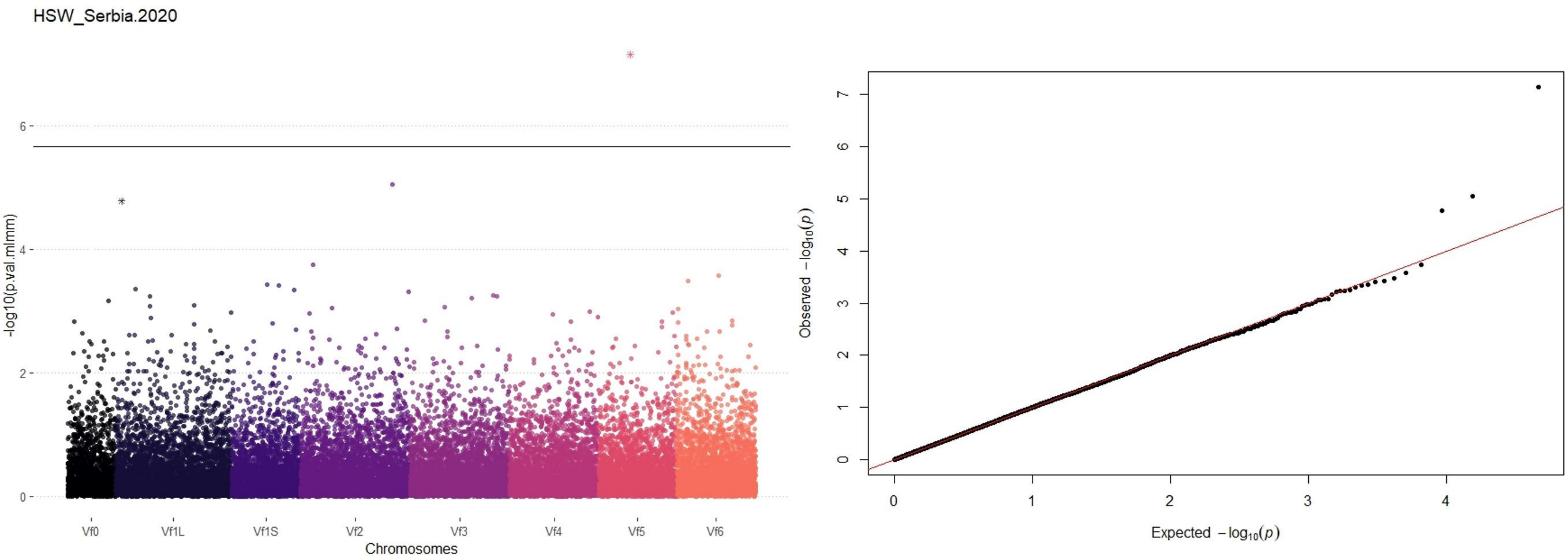

HSW_Serbia.2020
-log10(p.val.mlmm)
0
2
4
6
Vf0
Vf1L
Vf1S
Vf2
Vf3
Vf4
Vf5
Vf6
Chromosomes
Observed $-\log_{10}(p)$
Expected $-\log_{10}(p)$
0
1
2
3
4
5
6
7

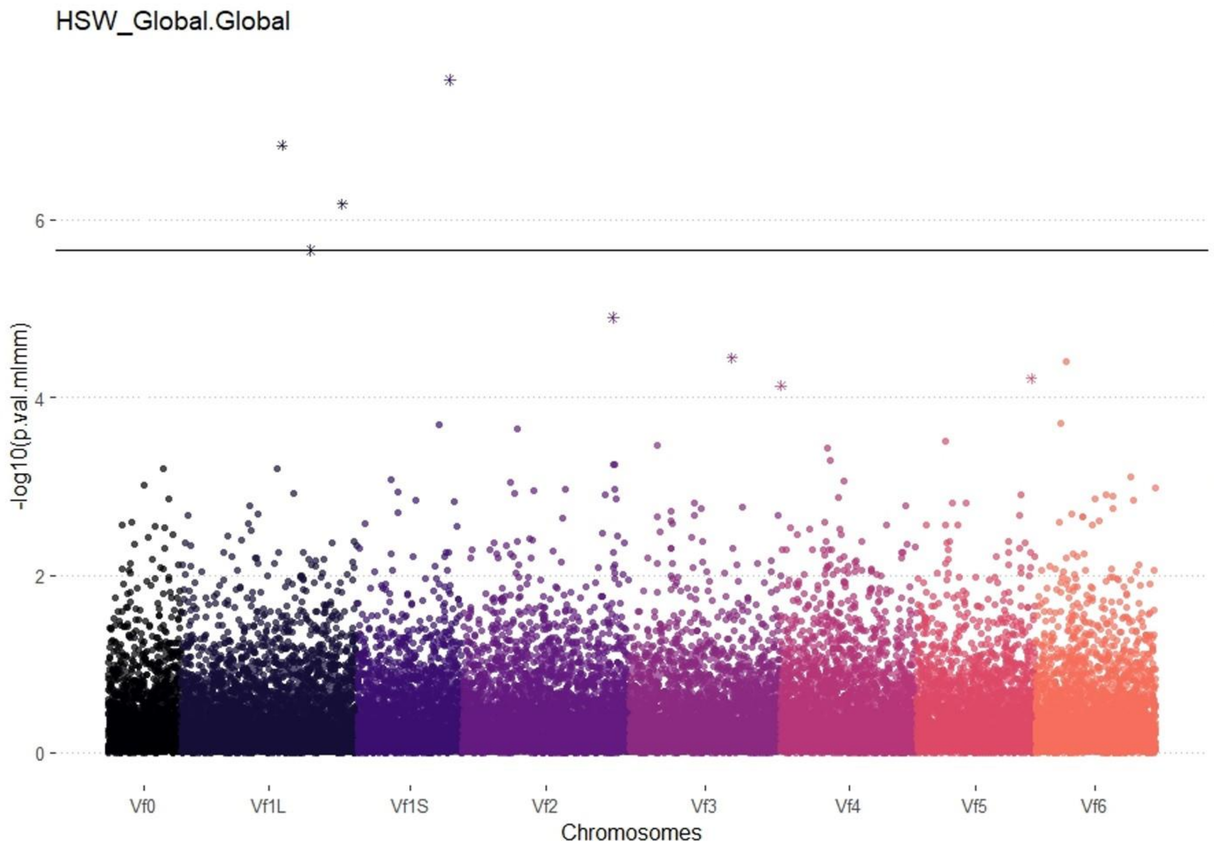
HSW_Global.Global
-log10(p.val.mlmm)
0
2
4
6
Vf0
Vf1L
Vf1S
Vf2
Vf3
Vf4
Vf5
Vf6
Chromosomes

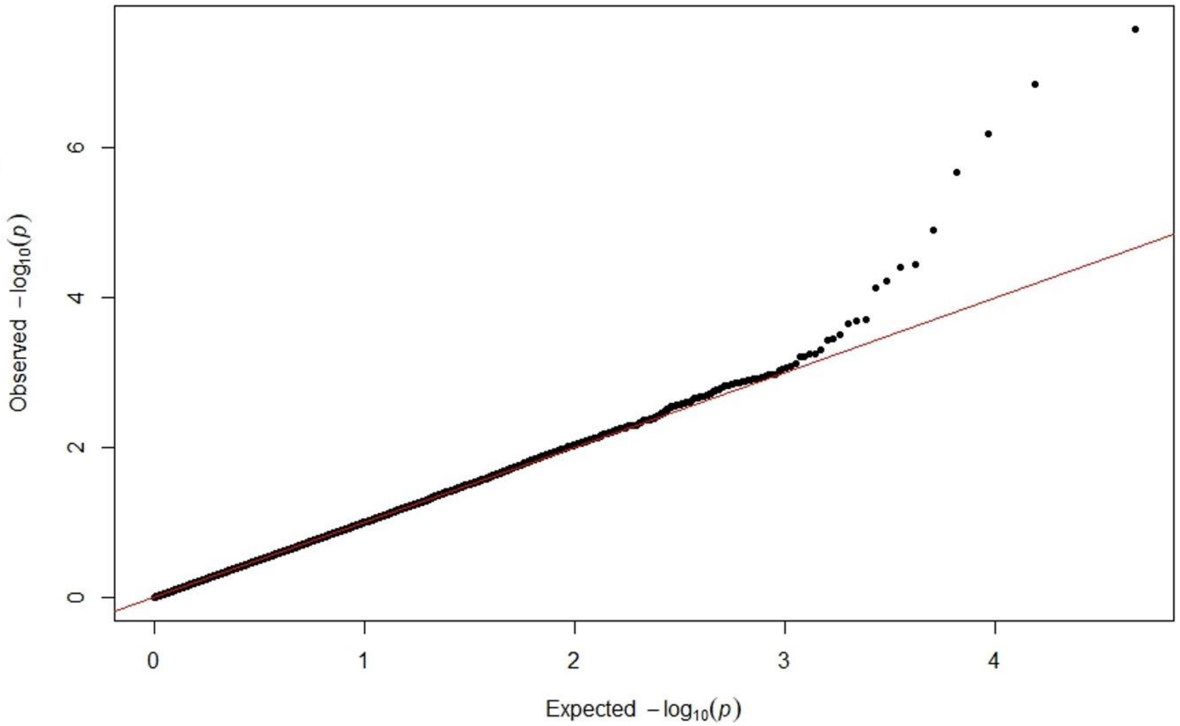
Observed $-\log_{10}(p)$
Expected $-\log_{10}(p)$
0
1
2
3
4
6

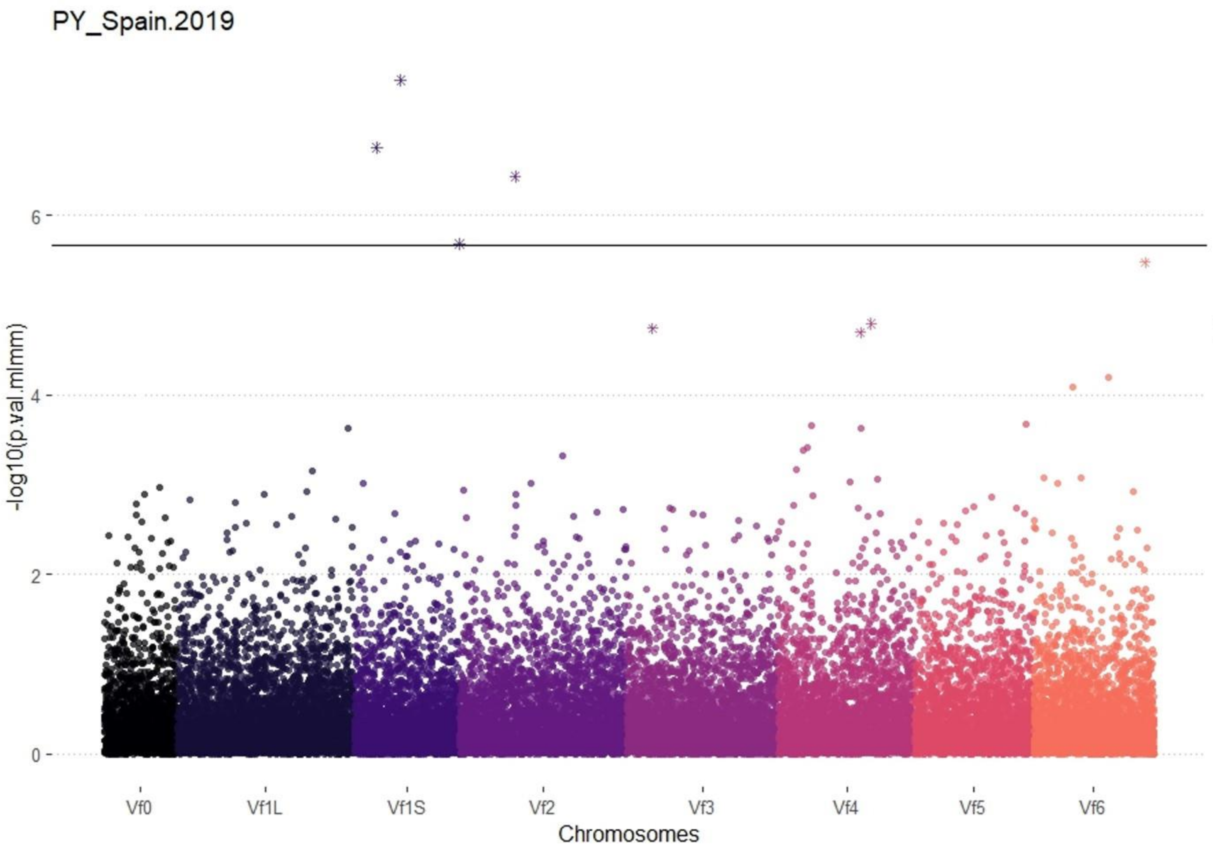

PY_Spain.2019
-log10(p.val.mlmm)
0
2
4
6
Vf0
Vf1L
Vf1S
Vf2
Vf3
Vf4
Vf5
Vf6
Chromosomes

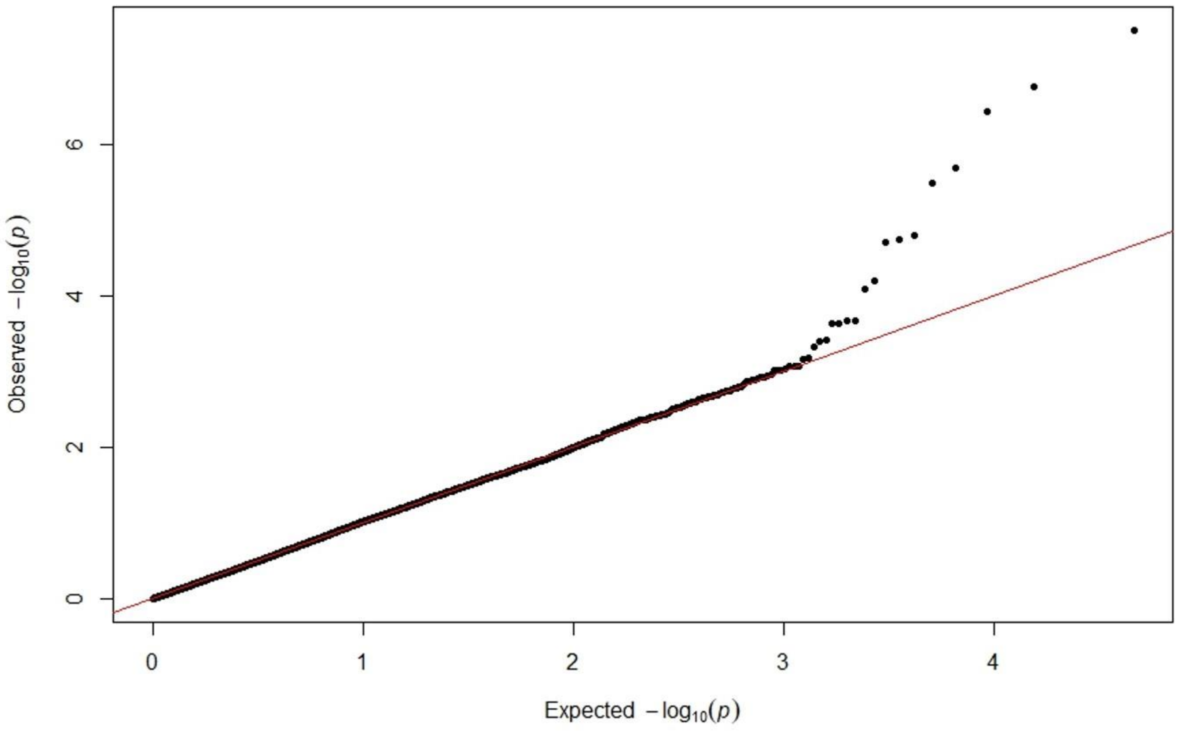

Observed $-\log_{10}(p)$
Expected $-\log_{10}(p)$
0
1
2
3
4
6

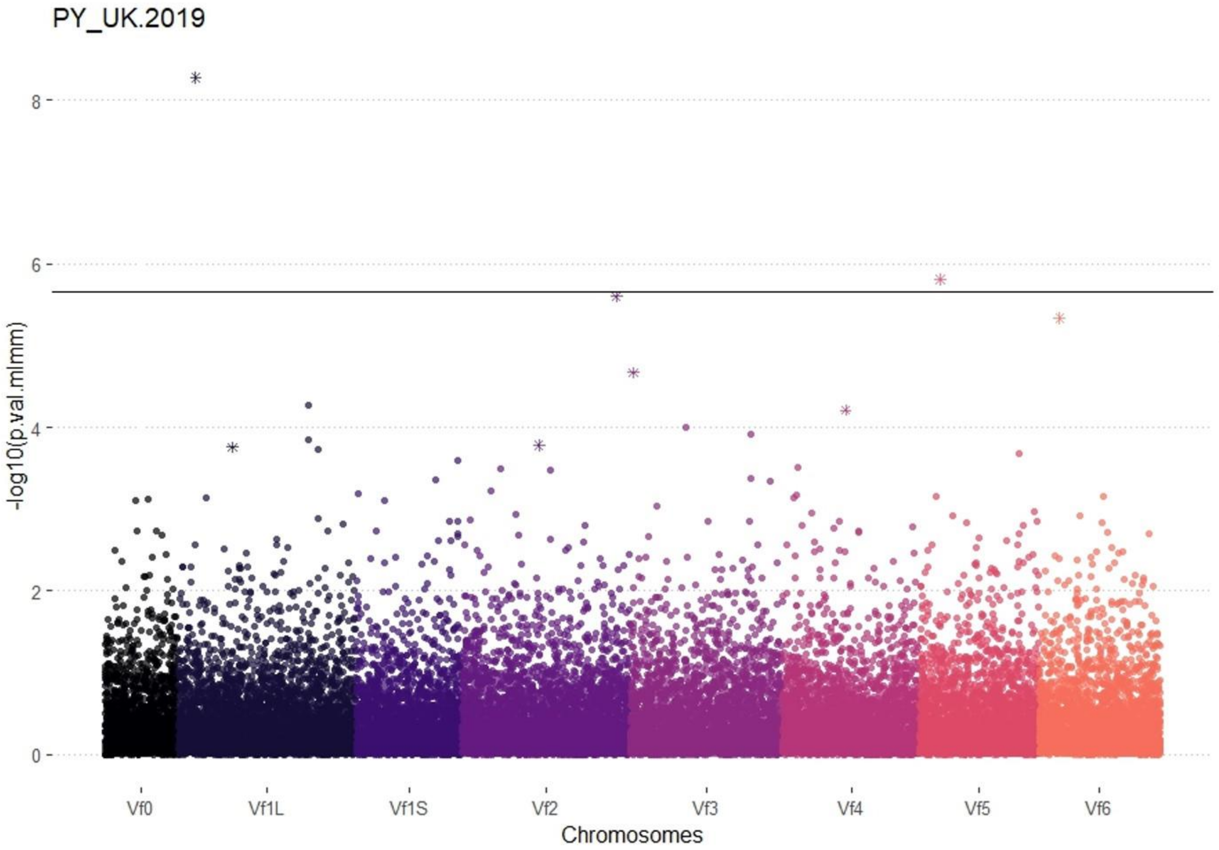

PY_UK.2019
-log10(p.val.mlmm)
0
2
4
6
8
Vf0
Vf1L
Vf1S
Vf2
Vf3
Vf4
Vf5
Vf6
Chromosomes

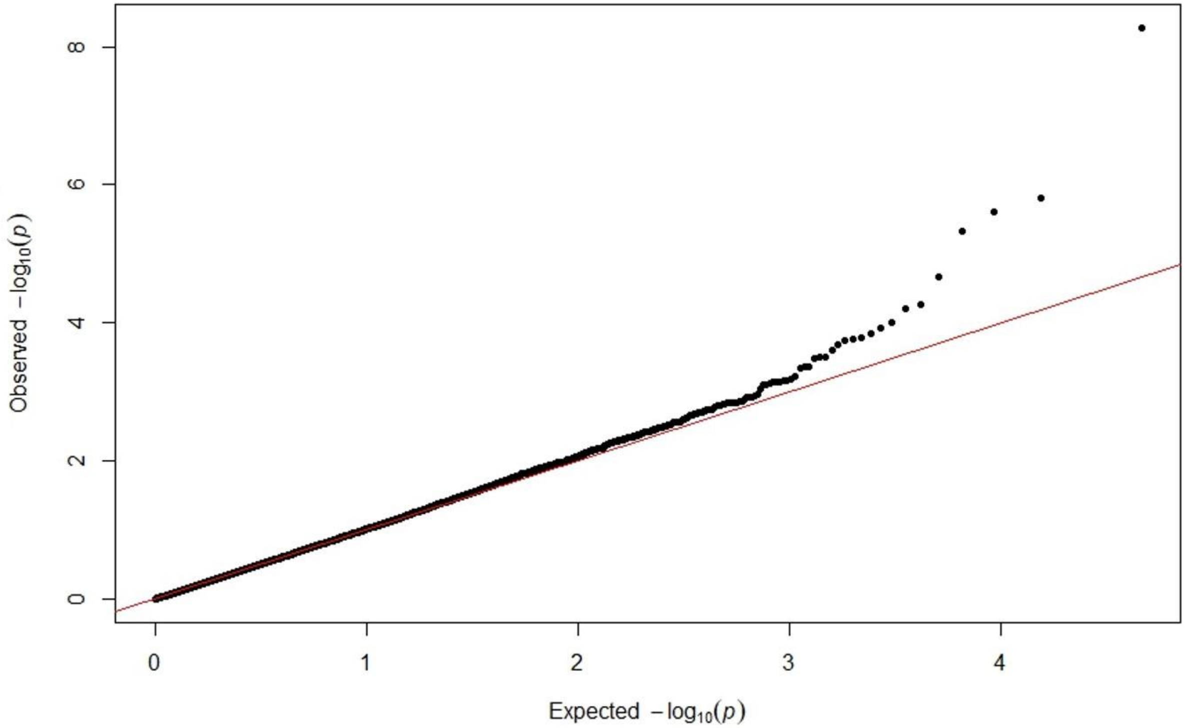

Observed $-\log_{10}(p)$
Expected $-\log_{10}(p)$
0
1
2
3
4
0
2
4
6
8
