# Supplementary material for: Genome-wide association study for yield-related traits in faba bean (Vicia faba L.)
Source: Front Plant Sci. 2024 Mar 13;15:1328690. doi: 10.3389/fpls.2024.1328690 (PMC10965552; doi:10.3389/fpls.2024.1328690)

Supplementary Figure S3. Distributions and correlations of each yield related trait in the different environments. The frequency distribution of each trait is shown on a central diagonal in the form of a histogram. Scatter plots between every pair of traits are shown in the areas below the diagonal, and numerical Pearson's correlation coefficients (r), between every pair of traits are shown in the areas above the diagonal. The red line represents the slope of the correlations. The x- and y- axes are the values of the measurements (HSW in grams and PY in kg). *, ** and *** indicate significance at $P < 0.05$, $P < 0.01$ and $P < 0.001$, respectively.

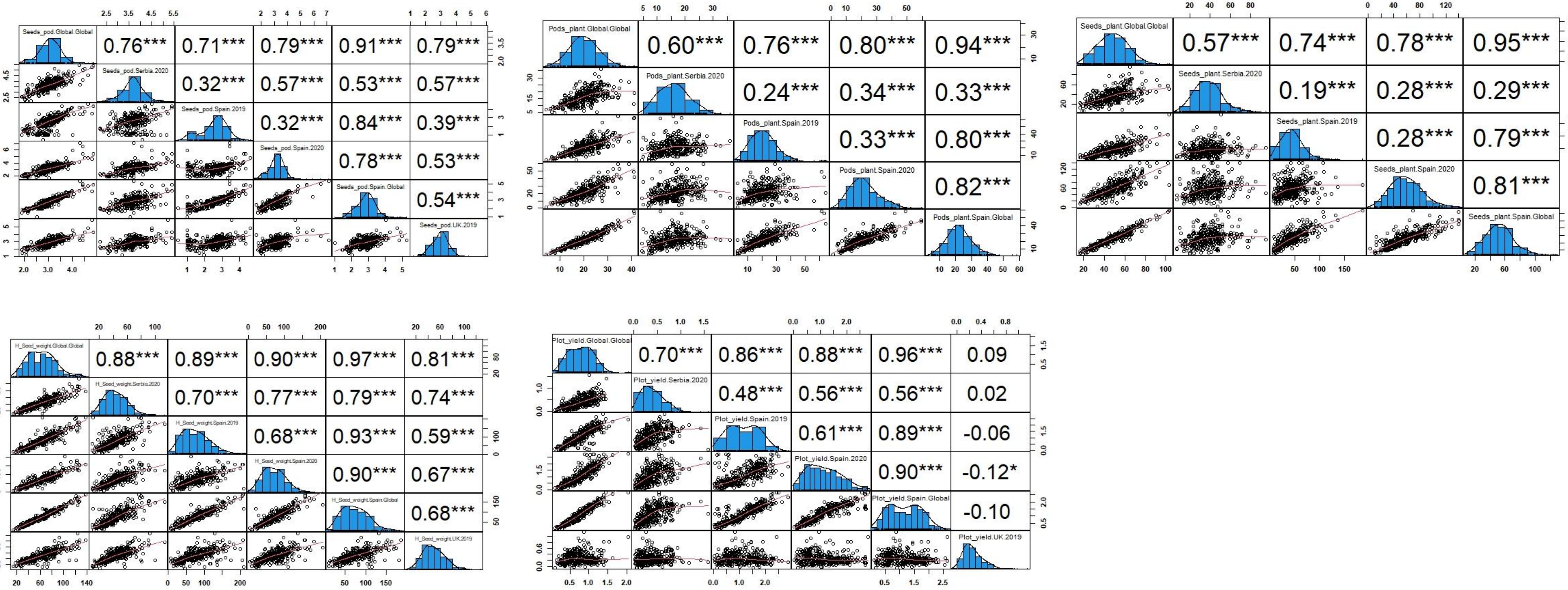

Supplement: Supplementary file 10 [file Image_3.pdf]
